# Availability of health facilities and utilization of maternal and newborn postnatal care in rural Malawi

**DOI:** 10.1186/s12884-019-2534-x

**Published:** 2019-12-17

**Authors:** Eunsoo Timothy Kim, Kavita Singh, Ilene S. Speizer, Gustavo Angeles, William Weiss

**Affiliations:** 1 Department of Maternal and Child Health, Gillings School of Global Public Health, University of North Carolina at Chapel Hill, Chapel Hill, NC USA; 2 MEASURE Evaluation/Carolina Population Center, University of North Carolina at Chapel Hill, Chapel Hill, NC USA; 3 Carolina Population Center, University of North Carolina at Chapel Hill, Chapel Hill, NC USA; 4 Department of International Health (Health Systems Program), Johns Hopkins Bloomberg School of Public Health, Baltimore, MD USA

**Keywords:** Postnatal care, Quality of care, Rural health, Maternal health, Newborn health, Malawi

## Abstract

**Background:**

This study explored the role of health facility availability as it relates to maternal and newborn PNC use in rural Malawi.

**Methods:**

Malawi Demographic and Health Survey (MDHS) 2015–16 data, MDHS 2015–16 household cluster GPS data, Malawi Service Provision Assessment (MSPA) 2013–14 data and MSPA 2013–14 facility GPS data were used. Household clusters were spatially linked with facilities using buffers. Descriptive analyses were performed and generalized estimating equations (GEE) were used to determine the effects of having different types of facilities at varying distances from household clusters on receipt of maternal and newborn PNC in rural Malawi.

**Results:**

In rural Malawi, around 96% of women had facilities providing PNC within 10 km of where they live. Among women who have clinic-level facilities within 5 km of where they live, around 25% had clinic-level facilities that provide PNC. For rural women who gave birth in the past 5 years preceding the survey, only about 3% received maternal PNC within 24 h and about 16% received maternal PNC within the first week. As for newborn PNC, 3% of newborns had PNC within 24 h and about 26% had newborn PNC within the first week. PNC mostly took place at facilities (94% for women and 95% for newborns). For women who delivered at home, having a health center providing PNC within 5 km was positively associated with maternal and newborn PNC. For women who delivered at facilities, having a health center providing PNC within 5 km was positively associated with maternal PNC and having a health center providing PNC between 5 km and 10 km was positively associated with both maternal and newborn PNC. Regardless of the place of delivery and distance band, having a clinic-level facility providing PNC did not have significant positive effects on maternal and newborn PNC.

**Conclusions:**

Providers should be trained to perform quality PNC at all facilities. It would also be important to address concerns related to health workers. Lastly, it would be key to increase community awareness about the importance of seeking timely PNC and about the utility of lower-level facilities for receiving preventative PNC.

## Background

Two of the targets for Sustainable Development Goal (SDG) 3 are to universally reduce the maternal mortality ratio to less than 70 maternal deaths per 100,000 live births and neonatal mortality to at least as low as 12 neonatal deaths per 1000 live births by 2030 [1]. The maternal mortality ratio in Malawi was 634 maternal deaths per 100,000 live births in 2015 [2]. This is higher than the sub-Saharan average of 546 maternal deaths per 100,000 live births in 2015 [3]. The neonatal mortality rate in Malawi was 22 deaths per 1000 live births in 2015 which was actually lower than the sub-Saharan average of 29 deaths per 1000 live births in 2015 [4] but still much higher than the SDG 3 target [1]. These statistics indicate that there is a need for further reduction in maternal and newborn mortality in Malawi and sub-Saharan Africa, more broadly. Malawi is one of the poorest countries in sub-Saharan Africa [5] and remains among the worst off on these key indicators despite the receipt of extensive amounts of overseas development funding [6]. In 2016, Malawi was the fifth largest recipient of overseas development assistance to the health-related sector [6]. The country received 718 million US dollars in 2016 from bilateral and multilateral donor organizations [6].

### Focus on postnatal care (PNC)

The postnatal period, typically defined as the first 42 days after birth, is particularly a vulnerable time for both the mothers and their newborns because they are at a high risk of mortality during this period [7]. Common causes of maternal mortality in this period are postpartum hemorrhage, sepsis and infection [7]. For newborns, common causes of mortality include intrapartum related birth asphyxia, infection and prematurity among others [7]. Timely and proper PNC can offer a critical opportunity to potentially reduce preventable maternal and newborn deaths [7]. In terms of supporting evidence, however, no prior study has rigorously examined the association between receipt of maternal PNC and the reduction of maternal mortality [8]. Studies linking newborn PNC and the reduction of newborn mortality, particularly for facility deliveries, are also difficult to find. One study by Singh, Brodish and Haney examined the associations between newborn PNC by provider type and neonatal mortality in 10 sub-Saharan African countries [9]. This study found that PNC within the first week by both skilled and unskilled health providers were associated with reduction of newborn deaths between days 2 and 7 and also between days 2 and 28 [9]. Despite the apparent lack of high quality evidence, the WHO nevertheless strongly recommends PNC for both the mothers and the newborns to reduce morbidity and mortality [8].

During PNC, the WHO recommends that mothers are assessed for vaginal bleeding, uterine contraction, fundal height, temperature, heart rate and blood pressure within the first 24 h and continually monitored for danger signs afterwards [8]. For newborns, it is recommended that they are assessed for clinical danger signs such as poor feeding, convulsions, fast breathing, severe chest in-drawing, lack of spontaneous movement, very high or low body temperature and jaundice [8].

It is important that the recommended number, timing and content of maternal and newborn PNC services are provided equitably across the geographic and socioeconomic spectrums. According to a recent systematic review and meta-analysis of PNC services in low- and middle-income countries, women living in urban areas had significantly higher odds of using PNC services compared to women living in rural areas (OR: 1.36; 95% CI: 1.01–1.81) [10]. This study concluded that within low- and middle-income settings, inequities exist in PNC use between rural and urban areas and also by education levels and socioeconomic status [10].

### The rural context

In Malawi, over 80% of men and women live in rural areas [11]. Rural areas have different sociodemographic, geographic, and health service characteristics compared to their urban counterparts across the world [12]. Some of these differences pertain to natural geography, local climate, tradition, culture, poverty level, resource availability, road infrastructure and transportation availability [12]. When it comes to the provision of quality healthcare services, ensuring availability of transport, medicines, skilled health workforce and even health facilities is crucial for remote areas [12]. However, because such resources are scarce in rural areas, policy decision-makers need to consider prioritizing the most pressing needs of the communities under tight budgetary constraints [12].

One example of this predicament is whether to strengthen existing primary healthcare facilities or invest in the construction of new primary healthcare facilities to create greater availability in remote areas [13]. In either case, adequate funding support, community engagement, and health workforce competency and retention are integral elements in order for primary healthcare to gain trust in the communities even after the issue of availability is resolved [14]. This is especially true since the density of physicians, nurses and midwives in Malawi was merely 3 per 10,000 population in 2010, which is critically lower than the World Health Organization (WHO) threshold of 23 physicians, nurses and midwives per 10,000 required to maintain essential levels of health services for mothers and children [15].

### Study aims

Among the diversity of multiple competing rural health needs, this study examines the role of health facility availability as it relates to maternal and newborn PNC use in rural Malawi. Three questions are addressed: (1) What is the availability of health facilities providing PNC in rural Malawi? (2) Where and when do mothers and newborns receive PNC in rural Malawi? and (3) What are the effects of having different types of health facilities at varying distances from household clusters on receipt of maternal and newborn PNC in rural Malawi? These are important policy questions that are expected to contribute to health services research in the context of Malawi and sub-Saharan Africa at large.

## Methods

### Data sources

Several datasets from the Malawi Demographic and Health Survey (MDHS) program were used to create a master analysis dataset. First, woman’s questionnaire data from the 2015–16 MDHS were retrieved [11]. The 2015–16 MDHS was implemented using a two-stage cluster sampling design. In the first stage, all 28 administrative districts in Malawi were stratified into 56 urban and rural strata [11]. For each stratum, a sample of standard enumeration areas (SEA) was selected based on the complete list of SEAs derived from the 2008 sampling frame of the Malawi Population and Housing Census [11]. Selection of the SEAs also occurred in two stages [11]. One hundred seventy-three urban SEAs and 677 rural SEAs were independently selected using probability proportional to the size of the SEA [11]. Then, 30 households from urban clusters and 33 households from rural clusters were selected using an equal probability systematic selection from the complete list of households in selected SEAs [11]. Selected SEAs with more than 250 households were segmented due to their large size and only one segment of households with probability proportional to the segment size was used for household listing [11]. The woman’s questionnaire collected various health and demographic data from all women in the reproductive age range between 15 and 49 years living in the selected households or who were found as visitors in the selected households on the day of the survey [11].

Second, the GPS coordinates of the centroids of the study clusters from the 2015–16 MDHS were linked with the woman’s data through unique identifiers. The published GPS coordinates are not the exact locations of the study clusters because they have been systematically displaced using the “random direction, random distance” method [16]. This was done in order to protect the respondents from the threat of identity disclosure [16]. Each GPS coordinate was displaced a distance of up to two kilometers if it was an urban cluster. The majority of rural clusters (99%) were displaced a distance of up to five kilometers [17]. A randomly selected 1 % of the rural clusters were displaced a distance of up to ten kilometers [17].

The third data source was the 2013–14 Malawi Service Provision Assessment (MSPA). These data were collected from a census of public and private facilities in all 28 districts including facilities run by the government, Christian Health Association of Malawi, other faith-based organizations, non-governmental organizations, private for-profit organizations and others [18]. The 2013–14 MSPA includes a total of four different questionnaires – Facility Inventory, Health Provider Interview, Observation Protocols and Exit Interview questionnaires with select clients [18]. The information about whether health facilities provide PNC services was obtained from the health provider interview. At each facility, the goal was to interview an average of eight health providers. For facilities that had less than eight providers, every provider was interviewed. For larger facilities, providers who were deemed most knowledgeable about their facility were selected for interview. If any health provider mentioned that he or she provides PNC services, the corresponding health facility was labeled as one providing PNC services.

Then, the GPS coordinates of the health facilities in 2013–14 MSPA were spatially linked with the woman’s questionnaire data from the 2015–16 MDHS. Three distance bands around household clusters were considered for the spatial linkage. Health facilities located between 0 km and 5 km (≤ 5 km) were grouped as the closest distance band. Health facilities located between 5 km and 10 km (> 5 km and ≤ 10 km) were grouped as the mid-range distance band. Health facilities located between 10 km and 15 km (> 10 km and ≤ 15 km) were grouped as the farthest distance band. Unlike the GPS coordinates of the 2015–16 MDHS household clusters, the GPS coordinates of the facilities were not displaced and reflect the true location [17]. Skiles, Burgert, Curtis and Spencer compared three data scenarios where methodological considerations of geographically linking DHS household clusters with health facilities were explored [19]. The study used the 2007 Rwanda Service Provision Assessment and the 2007–2008 Rwanda Interim Demographic and Health Survey [19]. In the study, the most ideal data scenario was having a census of all health facilities and undisplaced geographic locations of household clusters [19]. Other less ideal scenarios were either having a census of all health facilities and displaced household cluster locations or having a sample of health facilities and displaced household cluster locations [19]. The current study fits in with the second scenario where data were collected from a census of all health facilities in Malawi but the household cluster locations were randomly displaced. Skiles et al. reported that in the second scenario, using a Euclidean buffer of 5 km resulted in 5.9 to 9.2% of hospitals being misclassified, 7.0 to 12.4% of health centers being misclassified and 4.9 to 7.6% of health posts being misclassified [19]. The degree of misclassification error due to random displacement of household clusters in Malawi is expected to be similar to that reported in Skiles et al. but the possibility that there could be greater error in Malawian context cannot be ruled out completely.

The Woman’s Questionnaire data from the 2015–16 MDHS, the GPS coordinates of the 2015–16 MDHS household clusters, the 2013–14 MSPA data and the GPS coordinates of the 2013–14 MSPA facilities are all publicly available on the Demographic and Health Surveys Program website [20] upon request.

### Variables

All analyses were stratified by place of delivery. This is because types of health facilities and the proximity of these health facilities from household clusters are presumed to influence receipt of PNC differently based on where women delivered. Women who delivered at home may seek PNC at a health facility at the time of their choosing or receive a home visit by a health worker. In either circumstance, the proximity and the types of health facilities nearby women’s homes can potentially influence their receipt of PNC. However, women who delivered at health facilities face a slightly different set of options. After delivery at the facility, women may receive PNC on site before returning home for the first time, return home first then seek PNC at a later time at a facility or return home first then receive a postnatal home visit by a health worker. Due to these differences in care-seeking options based on place of delivery, there were several outcome variables used for analyses (in separate models). For women who delivered at home, the main outcomes were maternal PNC within 1 day of birth, newborn PNC within 1 day of birth, maternal PNC within 7 days of birth and newborn PNC within 7 days of birth. For women who delivered at health facilities, the main outcomes were maternal PNC between day 1 and day 7 and newborn PNC between day 1 and day 7. PNC between day 1 and day 7 was considered because women who received PNC right after delivery but before leaving the facility (to return home for the first time) will most likely do so in the first 24 h. Looking at this time interval can potentially capture the effects for women who were discharged and came back to a facility for a first or second postnatal check. As a supplementary analysis (see Additional file 1), PNC within the first day was still considered for women who delivered at health facilities to check for the assumption that some women receive PNC before discharge. In this case, the types of health facilities and their proximity should not have any significant positive influence on PNC seeking decisions because women are already at the facilities. All of the outcomes are binary with “1” indicating PNC in the specified time period and “0” otherwise. There were no women who responded “don’t know” for maternal PNC. For newborn PNC, less than 1% of the women responded “don’t know.” Among all rural women who delivered in the 5 years prior to the survey, less than 1% of the women had missing data for maternal and newborn PNC.

**Fig. 1:**
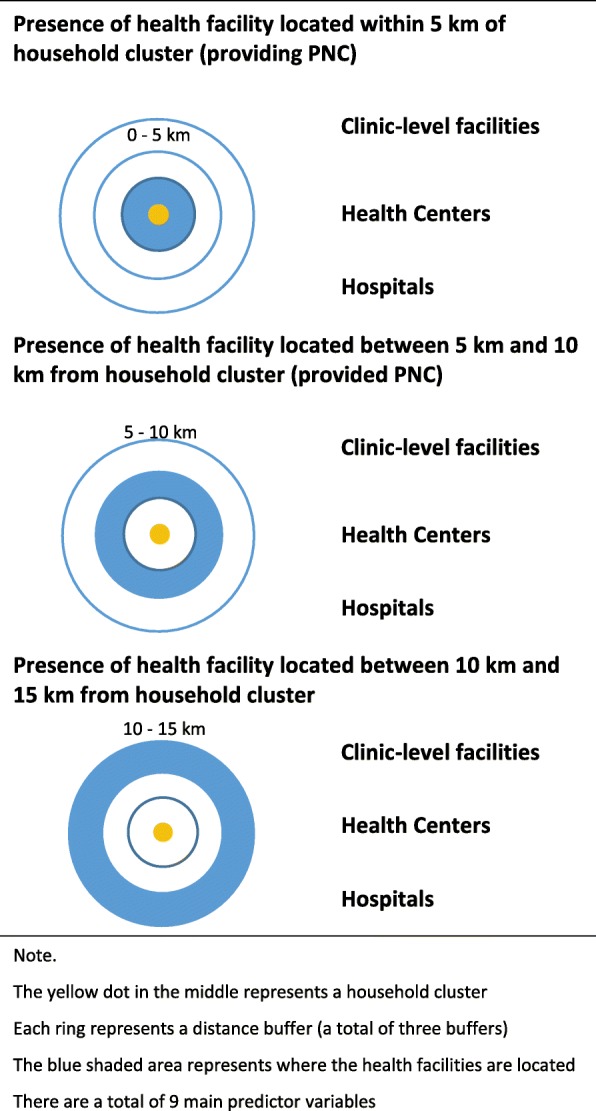
Main Predictors of the Analysis

There were three main types of binary indicators for health facilities: clinic-level facilities providing PNC, health centers providing PNC and hospitals providing PNC. Clinic-level facilities included maternities, dispensaries, clinics and health posts. Health centers only included facilities designated as health centers. Hospitals included central hospitals, district hospitals, rural/community hospitals and other hospitals. Health centers were set apart from other lower-level facilities because they comprise the largest number among all health facilities in Malawi [18]. In addition, compared to other lower-level facilities, health centers are much more likely to offer basic client services and delivery-related services in Malawi [18]. Types of facilities are meant to serve as indicators of the level of quality that can be provided at the facilities while three separate rings of buffers (0–5 km, 5–10 km and 10–15 km) indicate different levels of proximity or distance from the household clusters. See Fig. 1 for a visual illustration.

Covariates in the models included season in which women gave birth, ownership of TV or a radio, whether cost of treatment is a perceived problem, women’s age at the time of the survey, women’s education, women’s employment, household wealth, number of total births, newborn size, newborn sex, religion and region. For women who delivered at health facilities, cesarean section, whether or not women were checked before facility discharge and whether or not the newborns were checked before facility discharge were also included in the models. Number of antenatal visits was not included in the models due to potential endogeneity. Types of facilities and their proximity to household clusters could influence decisions regarding antenatal visits. Antenatal visits could also mediate the effects of facilities on PNC use, which is a classic case of endogeneity [21] where it is correlated with the error term when left in the models. Hence, the reported effects of health facilities are total effects, rather than direct effects, which account for the omitted mediated pathways (in the model) through antenatal visits.

Season in which women gave birth was coded as “warm-wet season (November to April)”, “winter-dry season (May to August)” or “hot-dry season (September and October)”. It was meant to proxy varying road conditions due to seasonal rainfalls. Ownership of TV or a radio was a binary variable meant to proxy potential exposure to health messages in the media. Obtaining money for treatment of any sickness being a big perceived problem was a binary variable meant to proxy financial barriers to accessing care. Women’s age at the time of the survey was coded as “15 – 24”, “25 – 34” and “35 – 49”. Women’s education was coded as having “no education”, “primary education” and “secondary education or higher”. Employment was a binary variable with “1” indicating currently working in either formal or non-formal sectors (including but not limited to agricultural, fishery and sales) and “0” otherwise. Household wealth was a rural-specific quintile variable constructed by DHS using principal components analysis [11]. It was coded as “poorest”, “poorer”, “middle”, “richer” or “richest”. Number of total births was coded as “1”, “2 – 3” and “4 or more”. Newborn size was subjectively reported by the respondents as either “very large”, “larger than average”, “average”, “smaller than average” or “very small”. This variable was meant to proxy potential maternal and/or newborn complications. Newborn sex was coded as “male” or “female”. Religion was coded as “Catholic”, “Other Christian” or “Muslim, no religion or other unspecified religion”. Those with no religion or other religion were less than 1% and for this reason, they were grouped together with those of Muslim faith, the second smallest group. Region was coded as “Northern”, “Central” and “Southern” which are three administrative regions in Malawi. Cesarean section was a binary variable meant to proxy maternal complications. Whether or not mothers received a check before facility discharge and whether or not newborns received a check before facility discharge were both coded as binary variables. These two variables and cesarean section were only included for women who delivered at health facilities.

### Analysis

A series of descriptive analyses were conducted. Then, generalized estimating equations (GEE) were used in STATA version 15.1 for all of the binary outcomes, with each in separate models. Clustering of households was accounted for by specifying the error correlation structure to be “exchangeable” which means that the variance-covariance matrix for each household cluster has an identical structure [22].

In equation form, the GEE models including the aforementioned outcomes, main predictors and covariates can be summed up below. For simplicity, the meaning of each unfamiliar notation is explained in the corresponding subscript.

$$ {Y}_{outcomes}={\alpha}_{intercept}+{\beta}_1{Clinics}_{0-5 km}+{\beta}_2{Health\ Centers}_{0-5 km}+{\beta}_3{Hospitals}_{0-5 km}+{\beta}_4{Clinics}_{5-10 km}+{\beta}_5{Health\ Centers}_{5-10 km}+{\beta}_6{Hospitals}_{5-10 km}+{\beta}_7{Clinics}_{10-15 km}+{\beta}_8{Health\ Centers}_{10-15 km}+{\beta}_9{Hospitals}_{10-15 km}+{\beta}_X{X}_{covariates}+{\varepsilon}_{error\ term} $$

The coefficients, denoted by ’s, were converted into differential effects in STATA using the “margins” command [23]. Differential effects were derived because they are more intuitive to understand than interpreting odds ratios. Differential effects can be obtained by first calculating the predicted probability of the referent category and the predicted probability of the alternative category and then taking the difference between the two. A general interpretation would be percentage point changes in the probability of the outcome given the alternative condition compared to being in the referent category. More specifically, a full interpretation for *β*_1_, for example, would be the percentage point changes in the average probability of receiving PNC associated with having a clinic-level facility providing PNC within 5 km compared to not having a clinic-level facility providing PNC within 5 km (averaged across all household clusters). This is controlling for the distribution of other health facilities providing PNC within 5 km, between 5 km and 10 km and between 10 km and 15 km as well as aforementioned covariates included in the models. In order to avoid repetition, however, a shortened version of the interpretation is presented in the results section.

Differential effects were only calculated and reported for the main predictors, as they are the focus of the analyses and covariates were carefully selected in order to obtain estimates that are as unbiased as possible for the main predictors. Lastly, all analyses, except those that are only focused on type of health facility and distance were weighted by individual women’s sampling probabilities.

## Results

### Background characteristics

In rural Malawi, a weighted total of about 11,576 women gave birth in the past 5 years preceding the survey (See Table 1). Close to two-fifths of the women were between ages 15 and 24 (37.7%) and more than two-fifths had four or more births (41.4%). Most women had no formal education (74.8%) and were currently working in either formal or informal work (67.9%). In addition, about 66% of women belonged in the poorest, poorer or middle wealth quintile and 62% did not own a TV or a radio. Most women were either Catholic or Christian (of other denominations). About 16% of women were either Muslim, had other unspecified religion or no religion at all. Those with no religion or other religion were less than 1% (not shown). In terms of perceived barriers in accessing general health care services, over half of the women considered cost of treatment (of any sickness) to be a problem (57.0%). Close to 90% of the women lived in Central and Southern regions of Malawi. For their most recent childbirth, the majority of rural women delivered at a health facility (93.0%) and only about 5% had a cesarean section. About half gave birth during the warm and wet season (48.4%) and reported having an average-sized newborn (49.3%). Among those who delivered at a health facility, about 46% reported receiving a maternal health check before discharge and 66% reported receiving a newborn health check before discharge. The sex of the newborns were close to even.

**Table 1:** Background characteristics of rural women who gave birth in the past 5 years preceding the survey in Malawi, MDHS 2015–16

	Total	
	n	%
Age		
15–24	4368	37.7%
25–34	4879	42.2%
35–49	2329	20.1%
Education		
None	8663	74.8%
Primary	2453	21.2%
Secondary or higher	460	4.0%
Employment		
Yes	7861	67.9%
No	3715	32.1%
Rural Wealth Quintile		
Poorest	2770	23.9%
Poorer	2510	21.7%
Middle	2307	19.9%
Richer	2134	18.4%
Richest	1855	16.0%
Religion		
Catholic	1877	16.2%
Other Christian	7884	68.1%
Muslim/other/no religion	1814	15.7%
Number of births		
1	2655	22.9%
2–3	4126	35.6%
4+	4795	41.4%
TV/Radio Ownership		
Yes	4370	38.1%
No	7111	61.9%
Cost of treatment (of any sickness) being a perceived problem		
Yes	6602	57.0%
No	4974	43.0%
Region of residence		
Northern	1360	11.8%
Central	4865	42.0%
Southern	5351	46.2%
Place of delivery		
Home	798	7.0%
Health Facility	10,599	93.0%
C-section		
Yes	624	5.4%
No	10,911	94.6%
Maternal health check before discharge from the health facility		
Yes	4847	45.9%
No	5711	54.1%
Newborn health check before discharge from the health facility		
Yes	6940	65.7%
No	3618	34.3%
Newborn size		
Very large	1112	9.7%
Larger than average	2831	24.7%
Average	5659	49.3%
Smaller than average	1354	11.8%
Very small	519	4.5%
Newborn sex		
Male	5805	50.2%
Female	5771	49.9%
Season in which women gave birth		
Warm-wet	5604	48.4%
Winer-dry	3733	32.3%
Hot-dry	2239	19.3%

There were 11,576 rural women who gave birth in the past 5 years preceding the survey (weighted)

Columns within a categorical variable sum to 100%

Observations are weighted counts

### Availability of health facility types

In terms of health facility availability, nearly 32% of rural women had no health facilities within 5 km of where they lived (See Table 2), with the Central and Northern regions having significantly higher percentages with no facilities (37.2 and 32.5% respectively). Very few women lived in areas where there were no health facilities within 10 km of where they lived (2.4%). However, the Northern region had a significantly higher percentage than the rest of the regions (7.2%). Within 15 km, nearly all women had a health facility.

**Table 2:** Availability of health facilities providing PNC within three distance bands of rural household clusters in Malawi, Malawi SPA 2013–14 & Malawi DHS 2015–16

	Total		Regions					
			Northern		Central		Southern	
	n	%	n	%	n	%	n	%
Women who have no health facilities								
within 5 km***	6088	31.5%	1205	32.5%	2450	37.2%	2433	27.0%
within 10 km***	463	2.4%	267	7.2%	113	1.7%	83	0.9%
within 15 km***	83	0.4%	54	1.5%	0	0.0%	29	0.3%
Women who have health facilities providing PNC								
within 5 km***	11,719	60.7%	2289	61.8%	3395	51.6%	6035	66.9%
between 5 km and 10 km (but none within 5 km)***	6743	34.9%	1061	28.6%	2882	43.8%	2800	31.0%
within 10 km***	18,462	95.6%	3350	90.4%	6277	95.4%	8835	97.9%
Type of facility providing PNC within 5 km								
Clinic-level facilities***	1064	5.5%	179	4.8%	275	4.2%	610	6.8%
Health centers***	8955	46.4%	1261	34.0%	2675	40.6%	5019	55.6%
Hospitals***	3334	17.3%	1005	27.1%	809	12.3%	1520	16.8%
Type of facility providing PNC between 5 km and 10 km								
Clinic-level facilities***	3364	17.4%	298	8.0%	786	11.9%	2280	25.3%
Health centers***	13,510	70.0%	1611	43.5%	4491	68.2%	7408	82.1%
Hospitals***	4662	24.1%	668	18.0%	1454	22.1%	2540	28.1%
Type of facility providing PNC between 10 km and 15 km								
Clinic-level facilities***	4016	20.8%	543	14.7%	859	13.1%	2614	29.0%
Health centers***	15,707	81.3%	2344	63.3%	5591	84.9%	7772	86.1%
Hospitals***	6758	35.0%	992	26.8%	2098	31.9%	3668	40.6%

Total number of observations is 19,315

Column percentages were reported corresponding to the category

Observations belonging in different categories of facilities within each buffer are not mutually exclusive (i.e. one observation could be counted multiple times if it has a clinic-level facility, a health center and a hospital all within 5 km of where it is)

***p < 0.001; bivariate chi-square tests were performed (each level of facility vs. regions)

Among women living within 5 km of any clinic-level facility, the percentage of those living within 5 km of clinic-level facilities that provide PNC services was 25% (See Table 3). For women living within 5 km of any health center or hospital, over 90% were living within 5 km of health centers and hospitals that provide PNC services. There was a similar pattern for other distance bands as well.

**Table 3:** Availability of PNC services among health facilities, Malawi DHS 2015–16

	Total	Facilities providing PNC	
	N	N	%
Within 5 km of household cluster			
Clinic-level	4226	1064	25.2%
Health center	9536	8955	93.9%
Hospital	3357	3334	99.3%
Between 5 km and 10 km of household cluster			
Clinic-level	8200	3364	41.0%
Health center	14,193	13,510	95.2%
Hospital	4817	4662	96.8%
Between 10 km and 15 km of household cluster			
Clinic-level	10,363	4016	38.8%
Health center	16,253	15,707	96.6%
Hospital	6813	6758	99.2%

Total number of observations is 19,315

Regarding availability of health facilities that provide PNC (See Table 2), very few women were living in areas where there were clinic-level health facilities providing PNC within 5 km (5.5%). In the same distance band (within 5 km), the percentage living in areas where there were health centers providing PNC was much higher at around 46%. In the Northern region, a significantly lower percentage of women lived in areas where there were health centers providing PNC within 5 km (34.0%). About 17% of women had hospitals providing PNC within 5 km of where they lived. Notably, the percentage having hospitals providing PNC within 5 km was highest for those living in the Northern region at 27%.

In the distance band between 5 km and 10 km, about 17% of women had clinic-level facilities providing PNC. In the Northern region, this percentage was significantly lower at around 8%. With regards to health centers providing PNC in the same distance band (between 5 km and 10 km), the percentage was much higher at around 70%. However, the Northern region had the lowest percentage yet again (43.5%). About 24% of women had hospitals providing PNC in this distance band (between 5 km and 10 km).

Between 10 km and 15 km, the percentage having clinic-level facilities providing PNC was about 21% and the percentage having health centers providing PNC was about 81%. The percentage having hospitals providing PNC was about 35%.

Taken together, nearly 61% of women had some type of health facility (clinic, health center, or hospital) providing PNC within 5 km of where they lived and of women who had no facility providing PNC within 5 km, about 35% had some type of facility PNC availability between 5 km and 10 km. This indicates that close to 96% of women had some degree of access to facility PNC within 10 km of where they lived. The Central region had the lowest percentage of women having a facility providing PNC within 5 km (51.6%) and the Northern region had the lowest percentage of women having a facility providing PNC within 10 km (90.4%).

### Place and timing of PNC

Among rural women who delivered in the past 5 years preceding the survey, about 3% reported receiving maternal PNC within the first day and about 16% reported receiving maternal PNC within the first week (See Table 4). For newborns, nearly 3% had PNC within the first day and about 26% had PNC within the first week.

**Table 4:** Percentages of rural women who gave birth in the past 5 years preceding the survey with maternal/newborn PNC, MDHS 2015–16

	Total	
	n	%
Maternal PNC within 24 h	367	3.2%
Maternal PNC within the first week	1877	16.2%
Newborn PNC within 24 h	340	2.9%
Newborn PNC within the first week	2992	25.9%

There were 11,576 rural women who gave birth in the past 5 years preceding the survey (weighted)

Most maternal and newborn PNC was provided at health facilities, 94 and 95% respectively (See Table 5). For those who delivered at home and received PNC, about 29% had their maternal PNC and about 16% had their newborn PNC also at home. Among the same group of women, 71% had their maternal PNC and 31% had their newborn PNC within the first 24 h. For those who delivered at health facilities and received PNC, about 10% had maternal PNC and about 4% had newborn PNC within the first 24 h.

**Table 5:** Place and timing of maternal/newborn PNC among rural women receiving PNC in Malawi, Malawi DHS 2015–16

			Place of Delivery			
	Total		Home		Health Facility	
	n	%	n	%	n	%
Place of Maternal PNC						***
Home	151	6.4%	46	29.5%	105	4.7%
Health Facility	2229	93.7%	110	70.5%	2119	95.3%
Timing of Maternal PNC						***
Within 24 h	329	14.0%	106	70.6%	223	10.1%
24 h to Day 3	142	6.0%	13	8.4%	129	5.9%
Day 3 to Week 1	1361	57.9%	14	9.4%	1347	61.2%
After Week 1	520	22.1%	17	11.6%	502	22.8%
Place of Newborn PNC						***
Home	221	4.7%	54	15.6%	167	3.8%
Health Facility	4505	95.3%	294	84.4%	4211	96.2%
Timing of Newborn PNC						***
Within 24 h	301	6.4%	108	31.1%	192	4.4%
24 h to Day 3	162	3.5%	26	7.6%	136	3.1%
Day 3 to Week 1	2462	52.3%	67	19.4%	2395	55.0%
After Week 1	1779	37.8%	146	41.9%	1634	37.5%

Note.

*p <0.05 **p <0.01 ***p <0.001

Total number of women who delivered at the health facility is 10,266; Number of observations for maternal outcomes is 10,083; Number of observations for newborn outcomes is 10,029

The outcomes were Maternal PNC between day 1 and day 7 or Newborn PNC between day 1 and day 7 in two separate GEE models

The main predictors (separate binary indicators) in the GEE models were whether or not there was: a clinic-level facility providing PNC within 5 km; a health center providing PNC within 5 km; a hospital providing PNC within 5 km; a clinic-level facility providing PNC between 5 km and 10 km; a health center providing PNC between 5 km and 10 km; a hospital providing PNC between 5 km and 10 km; a clinic-level facility providing PNC between 10 km and 15 km; a health center providing PNC between 10 km and 15 km; a hospital providing PNC between 10 km and 15 km

Covariates included in the GEE models were season in which women gave birth, ownership of TV or radio, whether cost of treatment is a perceived problem, women's age, women's education, women's employment, household wealth, number of births, newborn size, newborn sex, religion, region, cesarean section and whether or not the mother or the newborn (depending on the outcome) was checked before discharge from facility

### Interpretation of the reported GEE effects

All reported effects in the following sections were interpreted as positive or negative associations with maternal/newborn PNC (averaged across all household clusters) controlling for the existing distribution of health facilities within 5 km, between 5 km and 10 km and between 10 km and 15 km and also controlling for other covariates aforementioned. The referent group for these effects (of the health facilities) is not having the corresponding type of health facility in the same distance band. For example, the effect of having a health center within 5 km on maternal/newborn PNC would be in comparison to not having a health center within 5 km (averaged across all household clusters), controlling for the distribution of other health facilities within 5 km, between 5 km and 10 km and between 10 km and 15 km and also controlling for other covariates. It is important to keep in mind that these are population-average estimates.

### Effects of health facilities on PNC for women delivering at home

Among women who delivered at home, having a health center providing PNC within 5 km was positively associated with maternal PNC within the first day and within 7 days (See Table 6). Having a hospital providing PNC farther out (between 5 km and 10 km) was positively associated with maternal PNC within 7 days.

**Table 6:** The effects of different types and proximities of health facilities on maternal/newborn PNC among rural women who gave birth at home in Malawi, MDHS 2015–16

	Maternal				Newborn			
	PNC within 1 day		PNC within 7 days		PNC within 1 day		PNC within 7 days	
	DE	[95% CI]	DE	[95% CI]	DE	[95% CI]	DE	[95% CI]
Type and Proximity of Health Facilities								
Within 5 km of household cluster								
Clinic-level								
No facility (ref)	–	–	–	–	–	–	–	–
Facility	0.046	[−0.091, 0.184]	0.124	[−0.012, 0.259]	− 0.035	[− 0.179, 0.108]	0.042	[− 0.137, 0.221]
Health center								
No facility (ref)	–	–	–	–	–	–	–	–
Facility	0.079**	[0.020, 0.138]	0.073*	[0.012, 0.135]	0.068*	[0.003, 0.132]	0.063	[−0.020, 0.146]
Hospital								
No facility (ref)	–	–	–	–	–	–	–	–
Facility	0.028	[−0.053, 0.108]	−0.002	[− 0.087, 0.082]	0.137*	[0.012, 0.263]	0.117	[−0.053, 0.287]
Between 5 km and 10 km of household cluster								
Clinic-level								
No facility (ref)	–	–	–	–	–	–	–	–
Facility	−0.032	[−0.110, 0.046]	− 0.003	[− 0.076, 0.069]	0.030	[− 0.034, 0.094]	0.037	[− 0.045, 0.118]
Health center								
No facility (ref)	–	–	–	–	–	–	–	–
Facility	0.039	[−0.028, 0.105]	0.028	[−0.037, 0.092]	0.028	[−0.043, 0.099]	0.041	[−0.039, 0.121]
Hospital								
No facility (ref)	–	–	–	–	–	–	–	–
Facility	0.062	[−0.0004, 0.125]	0.073*	[0.012, 0.134]	0.030	[−0.029, 0.089]	0.066	[−0.015, 0.148]
Between 10 km and 15 km of household cluster								
Clinic-level								
No facility (ref)	–	–	–	–	–	–	–	–
Facility	− 0.062	[− 0.134, 0.010]	− 0.074	[− 0.150, 0.003]	−0.009	[− 0.077, 0.060]	0.006	[− 0.082, 0.093]
Health center								
No facility (ref)	–	–	–	–	–	–	–	–
Facility	−0.012	[−0.093, 0.070]	− 0.035	[− 0.119, 0.049]	0.050	[− 0.038, 0.138]	− 0.020	[− 0.123, 0.082]
Hospital								
No facility (ref)	–	–	–	–	–	–	–	–
Facility	0.027	[−0.034, 0.087]	0.025	[−0.042, 0.092]	− 0.000	[− 0.059, 0.059]	0.009	[− 0.072, 0.089]

Total number of women who delivered at home is 691; Number of observations for maternal outcomes is 665; Number of observations for newborn outcomes is 664

The outcomes were Maternal PNC within 1 day, Maternal PNC within 7 days, Newborn PNC within 1 day or Newborn PNC within 7 days in four separate GEE models

The main predictors (separate binary indicators) in the GEE models were whether or not there was: a clinic-level facility providing PNC within 5 km; a health center providing PNC within 5 km; a hospital providing PNC within 5 km; a clinic-level facility providing PNC between 5 km and 10 km; a health center providing PNC between 5 km and 10 km; a hospital providing PNC between 5 km and 10 km; a clinic-level facility providing PNC between 10 km and 15 km; a health center providing PNC between 10 km and 15 km; a hospital providing PNC between 10 km and 15 km

Covariates included in the GEE models were season in which women gave birth, ownership of TV or radio, whether cost of treatment is a perceived problem, women’s age, women’s education, women’s employment, household wealth, number of births, newborn size, newborn sex, religion and region

*p < 0.05 **p < 0.01

The effects of health facilities on newborn PNC showed slightly different patterns. Having a health center or a hospital providing PNC within 5 km was positively associated with newborn PNC within the first day. There were no other significant facility effects on newborn PNC.

### Effects of health facilities on PNC for women delivering at health facilities

Among women delivering in health facilities, having a health center providing PNC within 5 km was positively associated with maternal PNC between day 1 and day 7 (See Table 7). Having a health center providing PNC farther out (between 5 km and 10 km) was also positively associated with maternal PNC between day 1 and day 7. However, having a hospital providing PNC in this distance band (between 5 km and 10 km) was negatively associated with maternal PNC between day 1 and day 7.

**Table 7:** The effects of different types and proximities of health facilities on maternal/newborn PNC among rural women who gave birth at health facilities in Malawi, MDHS 2015–16

	Maternal		Newborn	
	PNC between Day 1 and Day 7		PNC between Day 1 and Day 7	
	DE	[95% CI]	DE	[95% CI]
Type and Proximity of Health Facilities				
Within 5 km of household cluster				
Clinic-level				
No facility (ref)	–	–	–	–
Facility	−0.023	[− 0.077, 0.032]	− 0.134**	[− 0.210, − 0.057]
Health center				
No facility (ref)	–	–	–	–
Facility	0.032*	[0.004, 0.059]	0.009	[−0.023, 0.041]
Hospital				
No facility (ref)	–	–	–	–
Facility	0.034	[−0.005, 0.074]	0.020	[−0.024, 0.065]
Between 5 km and 10 km of household cluster				
Clinic-level				
No facility (ref)	–	–	–	–
Facility	0.002	[−0.031, 0.034]	−0.047*	[− 0.090, − 0.004]
Health center				
No facility (ref)	–	–	–	–
Facility	0.044**	[0.014, 0.074]	0.069***	[0.034, 0.105]
Hospital				
No facility (ref)	–	–	–	–
Facility	−0.042**	[−0.072, − 0.011]	−0.010	[− 0.043, 0.023]
Between 10 km and 15 km of household cluster				
Clinic-level				
No facility (ref)	–	–	–	–
Facility	−0.004	[−0.036, 0.028]	0.030	[−0.010, 0.070]
Health center				
No facility (ref)	–	–	–	–
Facility	0.007	[−0.027, 0.041]	0.030	[−0.012, 0.072]
Hospital				
No facility (ref)	–	–	–	–
Facility	−0.002	[−0.031, 0.027]	− 0.013	[− 0.045, 0.019]

Total number of women who delivered at the health facility is 10,266; Number of observations for maternal outcomes is 10,083; Number of observations for newborn outcomes is 10,029

The outcomes were Maternal PNC between day 1 and day 7 or Newborn PNC between day 1 and day 7 in two separate GEE models

The main predictors (separate binary indicators) in the GEE models were whether or not there was: a clinic-level facility providing PNC within 5 km; a health center providing PNC within 5 km; a hospital providing PNC within 5 km; a clinic-level facility providing PNC between 5 km and 10 km; a health center providing PNC between 5 km and 10 km; a hospital providing PNC between 5 km and 10 km; a clinic-level facility providing PNC between 10 km and 15 km; a health center providing PNC between 10 km and 15 km; a hospital providing PNC between 10 km and 15 km

Covariates included in the GEE models were season in which women gave birth, ownership of TV or radio, whether cost of treatment is a perceived problem, women’s age, women’s education, women’s employment, household wealth, number of births, newborn size, newborn sex, religion, region, cesarean section and whether or not the mother or the newborn (depending on the outcome) was checked before discharge from facility

*p < 0.05 **p < 0.01 ***p < 0.001

With regards to newborn PNC, having a clinic-level facility providing PNC within 5 km was negatively associated with newborn PNC between day 1 and day 7. Having a clinic-level facility providing PNC farther out (between 5 km and 10 km) was also negatively associated with newborn PNC between day 1 and day 7 but having a health center providing PNC in this distance band (between 5 km and 10 km) was positively associated with newborn PNC between day 1 and day 7. For the effects of the control variables, see Additional file 2.

## Discussion

This study offered a unique opportunity to examine availability of different types of health facilities around rural household clusters in Malawi and how these facility types at varying distances influence maternal and newborn PNC. This study also highlighted that use of timely maternal and newborn PNC were very low among rural women who gave birth in the past 5 years preceding the survey. Only about 3% of all delivering mothers and 3% of newborns received PNC within the first 24 h of birth. Receipt of PNC within the first week of birth was also low at about 16% for mothers and 26% for newborns. This suggests that increasing timely maternal and newborn PNC in general warrants more programmatic focus and attention going forward. The discussion below offers additional insight as to how we can diversify strategies based on women’s place of delivery.

### Implications for PNC after home delivery

In terms of women delivering at home, having a health center providing PNC within 5 km was positively associated with maternal PNC. Having a health center providing PNC within 5 km was also positively associated with newborn PNC within the first day. The positive effects of health centers may generally be due to their offering of a wider range of basic client services and delivery-related services compared to other lower-level facilities [18] and therefore, they might have higher recognition to women living close by. Clinic-level facilities, on the other hand, may not have the same level of recognition or familiarity to women because many of these facilities do not provide the range of maternity services that the health centers can [18].

Based on these findings, a few strategies can be considered to increase utilization of PNC after home delivery. First, training health providers based in clinic-level facilities to provide both maternal and newborn PNC could lead to more sites where PNC is available. Among women who lived within 5 km of a clinic-level facility, only about 25% had clinic-level facilities providing PNC. Second, and more importantly, emphasizing quality by ensuring that the recommended content of PNC is provided in these lower-level facilities can be an effective intervention strategy. This is because clinic-level facilities already providing PNC were not shown to be positively associated with maternal or newborn PNC. It would be important to support these lower-level facilities to provide quality PNC and to inform local communities of such changes in order to encourage use of these existing resources. Third, community health workers could also receive regular training and work closely with clinic-level facilities and health centers to provide PNC services in women’s homes.

However, lack of provider training may not be the only issue. A study specifically looking at provision of PNC and uptake in four African countries reported that structures of organizational support and a system of accountability for health workers were not properly in place in Malawi (one of the four countries), leaving workers demotivated to deliver quality PNC [24]. Another study looking at health workers’ perspectives on worker retention and motivation in Malawi also found that major demotivating factors for health workers were generally low salary, unclear job descriptions, unequal opportunities for training, lack of an appropriate performance appraisal system and lack of supervision and feedback from the management [25]. These general issues would also be a hindrance to health workers providing quality PNC as well. Hence, coupled with provider training, there may need to be a wider health sector reform at the district management and health facility levels to establish a working accountability, supervision and feedback mechanism for providing quality PNC [24, 26].

Fourth, raising community-awareness about the importance of timely PNC and the utility of clinic-level facilities for preventative PNC may also increase demand for service utilization, as there already seems to be high availability of health facilities providing PNC both within 5 km and within 10 km and also high acceptability of health facilities. In 2015 and 2016, 91% of deliveries occurred in a facility setting in Malawi [11]. Hence, given the general availability and acceptability of health facilities, knowing what is offered at clinic-level facilities and health centers can be an important driver of service use. However, it would be important to couple this with quality improvement for PNC services so that women have assurance that they will receive quality preventative PNC in the lower-level facilities. One of the primary roles of clinic-level facilities is to provide preventative health services [27].

There is some evidence from a review study of demand-side interventions for maternal care that community-based mobilizations where trained facilitators led various forms of discussion groups to enhance knowledge and awareness of health problems, resulted in increased utilization of facility-based maternal care [28]. This review study only considered antenatal visits, facility-based delivery and delivery with skilled birth attendants as utilization outcomes [28]. Nonetheless, it showed potential that community-based mobilization interventions can increase use of facility-based maternal services [28].

Between 5 km and 10 km, having a hospital providing PNC, compared to not having one, was positively associated with maternal PNC. This indicates that at this distance, women delivering at homes preferred the hospital for maternal PNC visits. As mentioned previously, if clinics and health centers closer by (within 5 km) provide adequate PNC to communities, high patient load and burden of care could be shifted away from hospitals both near (within 5 km) and far (between 5 km and 10 km).

Strengthening the capacity of lower-level facilities is also beneficial for home PNC visits. This is because facilities will be able to provide more support and training to affiliated community attendants for home outreach and referrals. An evaluation of context-specific interventions designed to improve PNC in Africa found that community health workers can effectively operate as a bridge between women and the formal health sector [24]. Facility-initiated interventions such as training, supervision and other incentive structures were found to strengthen the professional connection between the health facilities and the community health workers and also increase their motivations as well [24]. Community health workers with stronger links to the health facilities are able to identify referral cases during home visits and encourage more women to seek facility-based care [24].

### Implications for PNC after facility delivery

For women delivering at health facilities, a general intervention strategy should involve a streamlined referral system in which all women delivering at the facility are either visited by a health provider while in the facility or encouraged to visit the postnatal ward within the same facility site before going home for the first time after delivery. For women who cannot follow this suggestion, health providers should refer them to health facilities providing PNC near their residences and consider a home visit strategy. As with the proposed intervention strategies for women delivering at home, supporting clinic-level facilities as well as health centers to provide high quality PNC will help women delivering at health facilities to follow up on their providers’ referrals since only health centers were found positively associated with PNC visits. Along with the referrals, the literature suggests that providers should also be mindful about their attitude in giving a thorough explanation of the purpose and the benefits of care to women in general [29, 30] and when providing maternal health services [31, 32]. In Malawi, a commonly cited reason for not wanting to receive care at the health facilities is providers’ lack of explanation and poor attitude [29–32]. Although asking health providers to be more “mindful” about their demeanor seems like a trivial task, it should be done considering the challenges providers in Malawi face [25, 33]. The Malawian health system struggles with severe understaffing [25, 33] and inappropriate skill mix of health providers, especially in the delivery ward [33]. As a result, health providers often report having physical, psychological and emotional stress [33]. Adding another task, albeit simple, may not be effective with already over-burdened staff [25, 33] if it is not complemented with appropriate organizational and sector-wide reforms boosting health worker motivations to provide quality care [24, 26, 34, 35]. As discussed before, key determinants of health worker motivation include organizational support, accountability, feedback, supervision and incentive structures [24, 26, 34, 35].

Taken together, the proposed strategies have the potential not only to be useful for encouraging timely PNC within the first day but also making it easier for subsequent postnatal visits which are recommended three more times: on day 3, between the first and second week and at 6 weeks [36]. Importantly, these strategies are also consistent with the Malawi health sector strategic plan for 2017 to 2022 which was published in April of 2017 [27]. This 5-year strategic plan issued by the Malawian Ministry of Health outlines that the Malawian government intends to “increase equitable access to and quality of health care services” in the provision of the “Essential Health Package” [27]. The “Essential Health Package” encompasses a wide range of important health services including treatment of postpartum hemorrhage [27]. However, preventative PNC was not clearly delineated in the “Essential Health Package”. Nevertheless, it is still considered a key component of the essential delivery-related services [7]. A recommendation would be to have both maternal and newborn PNC clearly listed as essential services in the package. Other relevant objectives of the Malawi health sector strategic plan II are improving the quality of training and performance for health workers and promoting healthy behaviors through community education [27].

### Availability of health facilities providing PNC

Close to two-thirds of rural women had a health facility providing PNC within 5 km of where they lived with some regional variation. In the larger 10 km distance band, almost all rural women had health facilities providing PNC. In addition, facilities located beyond 10 km, regardless of type, were not significant in predicting maternal or newborn PNC. Together, these findings suggest that investing in the construction of new clinics or health centers merely to increase availability around rural communities may be a redundant effort, having little to no effect on encouraging higher utilization of PNC. Instead, resources could be directed towards raising community awareness about the importance of timely PNC, supporting quality improvement initiatives for lower-level facilities, ensuring convenient means of transportation and lowering costs of getting to the facilities and receiving care. Among rural Malawian women, only about 4% possessed either motorcycles, scooters, cars or trucks in their households for transportation [11]. About 42% listed bicycles as means of transportation [11]. In addition, over half of the women in this study responded that cost of treatment (of any sickness) was a perceived barrier to seeking care.

### Possible explanations for negative health facility effects

There were three statistically significant negative effects of health facilities for women who delivered at health facilities. Although the exact reasons are unknown, this could perhaps be due to several factors: (1) the selection effect of clinic-level facilities being placed in areas where health outcomes are generally poorer and utilization of health services including PNC are low. Hence, their presence is associated with lower PNC use; (2) women having little to no awareness that PNC services are offered, especially in clinic-level facilities; (3) women having low confidence that clinic-level facilities can provide quality PNC; (4) women not being able to follow through with recommended PNC after discharge for their newborns; or (5) women not being convinced that seeking additional PNC is necessary after discharge.

### Limitations

There are a few limitations in the study. First, women who may have been at very high risk and died are not represented in the sample. This could potentially lead to a misrepresentation of the coverage of PNC use. Second, due to missing information, some facilities were not matched in the process of merging facility locations with the provider interviews to determine whether facilities provided PNC service. However, the number of unmatched facilities was small at around 3%. Third, the design effect of stratification was not accounted for in the analyses. Women’s sampling weights and clustering were applied to individual cases however. For comparison, sensitivity analyses were conducted taking into account the full design effect (stratification, clustering and women’s sampling weights) with logistic regression and the results were nearly identical to the main results presented in this study (see Additional file 3). The magnitude of the effects were very similar and the signs and the significance of the effects were exactly the same. Fourth, GPS coordinates of the household clusters have been displaced at a random angle and a random distance from their original locations. Although using buffers with reasonable distances to link facilities and household clusters is expected to somewhat account for the displacement [17], it introduces “noise” in the analysis nonetheless. However, even in the presence of such “noise”, knowing the exact locations and characteristics of all operational health facilities (based on 2013 data) in Malawi allowed the unique opportunity to investigate the study’s research questions.

## Conclusions

The main findings of the study offer important insights for future policy considerations in the context of rural Malawi and comparable regions in sub-Saharan Africa at large. Clinic-level facilities and health centers that currently do not provide PNC should be supported to provide quality PNC to women and newborns. Quality improvement strategies can be considered for lower-level facilities that already provide PNC. Women who deliver at health facilities should receive their first PNC visit (for both the mother and the newborn) before they leave the facility as a standard of practice. Health providers should also mindfully explain the importance of timely PNC and refer women to facilities providing PNC near their residences for further visits. Home visits can also be promoted to reach both women who delivered in facilities as well as the smaller number of women who did not. Lastly, allocating resources to the construction of new facilities does not seem to be a good strategy for increasing utilization of PNC. Instead, more effective strategies are: (1) training providers to be able to perform quality PNC at all facilities; (2) establishing a working system of support, accountability, feedback, supervision and incentives at the organizational and district levels to mitigate issues of staff frustrations and lack of motivation; and (3) increasing community awareness about the importance of seeking timely PNC and about the utility of lower-level facilities for receiving preventative PNC.

## Supplementary information

**Additional file 1.** The effects of different types and proximities of health facilities on maternal/newborn PNC within 1 day among rural women who gave birth at health facilities in Malawi, Malawi DHS 2015–16.

**Additional file 2.** Stata log output for the covariate effects.

**Additional file 3.** Stata log output for sensitivity analyses with the full design effect.

## Data Availability

The datasets analyzed during the current study are available upon request on the DHS Program website. We encourage interested researchers to request permission to download these datasets directly from the DHS Program. The conditions of use for DHS datasets do not allow us to share study datasets with other researchers without DHS Program’s written consent. Please see the following website for details on the terms of use: https://dhsprogram.com/data/terms-of-use.cfm.

## Abbreviations

GEE: Generalized estimating equations
MDHS: Malawi Demographic and Health Survey
MSPA: Malawi Service Provision Assessment
PNC: Postnatal care
SDG: Sustainable Development Goal
WHO: World Health Organization
