# Supplementary material for: Availability of health facilities and utilization of maternal and newborn postnatal care in rural Malawi
Source: BMC Pregnancy Childbirth. 2019 Dec 17;19:503. doi: 10.1186/s12884-019-2534-x (PMC6918704; doi:10.1186/s12884-019-2534-x)
Supplement: Supplementary file 1 — Additional file 1. The effects of different types and proximities of health facilities on maternal/newborn PNC within 1 day among rural women who gave birth at health facilities in Malawi, Malawi DHS 2015–16. [file 12884_2019_2534_MOESM1_ESM.pdf]

Additional file 1

Appendix Table 1. The effects of different types and proximities of health facilities on maternal/newborn PNC within 1 day among rural women who gave birth at health facilities in Malawi, MDHS 2015-16

	Maternal		Newborn	
	PNC within 1 day		PNC within 1 day	
	DE	[95% CI]	DE	[95% CI]
**Type and Proximity of Health Facilities**				
Within 5 km of household cluster				
Clinic-level				
No facility (ref)	-	-	-	-
Facility	0.014	[-0.004, 0.033]	0.003	[-0.013, 0.018]
Health center				
No facility (ref)	-	-	-	-
Facility	-0.003	[-0.011, 0.005]	-0.002	[-0.009, 0.006]
Hospital				
No facility (ref)	-	-	-	-
Facility	-0.02*	[-0.034, -0.004]	-0.007	[-0.018, 0.005]
Between 5 km and 10 km of household cluster				
Clinic-level				
No facility (ref)	-	-	-	-
Facility	0.004	[-0.007, 0.015]	0.006	[-0.004, 0.015]
Health center				
No facility (ref)	-	-	-	-
Facility	0.000	[-0.009, 0.010]	0.000	[-0.008, 0.008]
Hospital				
No facility (ref)	-	-	-	-
Facility	-0.002	[-0.011, 0.007]	-0.004	[-0.012, 0.003]
Between 10 km and 15 km of household cluster				
Clinic-level				

No facility (ref)	-	-	-	-
Facility	-0.009	[-0.019, 0.001]	-0.004	[-0.012, 0.004]
Health center				
No facility (ref)	-	-	-	-
Facility	-0.006	[-0.015, 0.003]	-0.000	[-0.010, 0.009]
Hospital				
No facility (ref)	-	-	-	-
Facility	-0.008	[-0.017, 0.001]	-0.009*	[-0.017, -0.001]

Note.

*p<0.05

Total number of women who received maternal PNC is 10,231; Total number of newborns who received PNC is 10,173;
Number of observations for maternal outcomes is 10,083; Number of observations for newborn outcomes is 10,029

The outcomes were Maternal PNC within 1 day or Newborn PNC within 1 day in two separate GEE models

The main predictors (separate binary indicators) in the GEE models were whether or not there was: a clinic-level facility providing PNC within 5 km; a health center providing PNC within 5 km; a hospital providing PNC within 5 km; a clinic-level facility providing PNC between 5 km and 10 km; a health center providing PNC between 5 km and 10 km; a hospital providing PNC between 5 km and 10 km; a clinic-level facility providing PNC between 10 km and 15 km; a health center providing PNC between 10 km and 15 km; a hospital providing PNC between 10 km and 15 km

Covariates included in the GEE models were season in which women gave birth, ownership of TV or radio, whether cost of treatment is a perceived problem, women's age, women's education, women's employment, household wealth, number of births, newborn size, newborn sex, religion, region, cesarean section and whether or not the mother or the newborn (depending on the outcome) was checked before discharge from facility
