# Supplementary material for: Availability of health facilities and utilization of maternal and newborn postnatal care in rural Malawi
Source: BMC Pregnancy Childbirth. 2019 Dec 17;19:503. doi: 10.1186/s12884-019-2534-x (PMC6918704; doi:10.1186/s12884-019-2534-x)
Supplement: Supplementary file 2 — Additional file 2. Stata log output for the covariate effects. [file 12884_2019_2534_MOESM2_ESM.pdf]

List of variables:

/***Set up***/
DHSCLUST = clustering variable
Weight = individual sampling weight
del = place of delivery (0 for home delivery and 1 for facility delivery)
del2 = place of delivery (1 for home delivery and 0 for facility delivery)
v021 = primary sampling unit
v022 = stratification used
residence = urban/rural (1 for rural and 0 for urban)

/***Outcomes***/

binarympnc1 = maternal postnatal care within 1 day
binarympnc7 = maternal postnatal care within 7 days
binarypnc1 = newborn postnatal care within 1 day
binarypnc7 = newborn postnatal care within 7 days

/***Facility Indicators***/

clinic5_pnc = clinic-level facilities providing postnatal care within 5 km of household cluster
hc5_pnc = health centers providing postnatal care within 5 km of household cluster
hosp5_pnc = hospitals providing postnatal care within 5 km of household cluster
clinic10_pnc = clinic-level facilities providing postnatal care between 5 km and 10 km of household cluster
hc10_pnc = health centers providing postnatal care between 5 km and 10 km of household cluster
hosp10_pnc = hospitals providing postnatal care between 5 km and 10 km of household cluster
clinic15_pnc = clinic-level facilities providing postnatal care between 10 km and 15 km of household cluster
hc15_pnc = health centers providing postnatal care between 10 km and 15 km of household cluster
hosp15_pnc = hospitals providing postnatal care between 10 km and 15 km of household cluster

/***Covariates***/

religion = women's religion
v024 = region of the country where women live
seasons = seasons in which women gave birth
tvradio = ownership of TV or radio
moneyfortreat = cost of treatment being a perceived problem
age = women's age
education = women's education
employment = women's employment
wealthrural = rural-specific household wealth quintile
birthnum = women's number of births
nsize = newborn size
nsex = newborn sex
mhealthcheckb4 = maternal health checked before facility discharge (for facility births)
newborncheckb4 = newborn health checked before facility discharge (for facility births)
csect = receipt of cesarean section

```
. /*********************************
> GEE analyses: Full models
> *********************************/
.
. /******
> Home
> Delivery
> Only
> *******/
.
. ***Analysis commands
. xtgee binarympnc1 clinic5_pnc hc5_pnc hosp5_pnc ///
> clinic10_pnc hc10_pnc hosp10_pnc ///
> clinic15_pnc hc15_pnc hosp15_pnc ///
> i.religion i.v024 i.seasons i.tvradio i.moneyfortreat i.age i.education i.employment
i.wealthrural ///
> i.birthnum ib3.nsize i.nsex [pweight=weight] if residence == 1 & del == 0,
family(binomial) link(logit) corr(exchange
> able)

Iteration 1: tolerance = .00140315
Iteration 2: tolerance = .00007196
Iteration 3: tolerance = 3.687e-06
Iteration 4: tolerance = 1.883e-07

GEE population-averaged model                   Number of obs     =        665
Group variable:                    DHSCLUST      Number of groups  =        323
Link:                                 logit      Obs per group:
Family:                            binomial                   min =          1
Correlation:                   exchangeable                   avg =        2.2
                                                              max =         16
                                                Wald chi2(33)     =      80.69
Scale parameter:                          1     Prob > chi2       =     0.0000

                                (Std. Err. adjusted for clustering on DHSCLUST)
------------------------------------------------------------------------------
                   |               Robust
       binarympnc1 |      Coef.   Std. Err.      z    P>|z|     [95% Conf. Interval]
-------------------+----------------------------------------------------------
       clinic5_pnc |    .470705   .7125274     0.66   0.509     -.925823    1.867233
           hc5_pnc |   .8026485   .3099137     2.59   0.010     .1952288    1.410068
         hosp5_pnc |   .2819961   .4174005     0.68   0.499    -.5360938    1.100086
      clinic10_pnc |  -.3242573   .4094083    -0.79   0.428    -1.126683    .4781682
          hc10_pnc |    .392781   .3450009     1.14   0.255    -.2834084     1.06897
        hosp10_pnc |   .6332002   .3250175     1.95   0.051    -.0038225    1.270223
      clinic15_pnc |  -.6287679   .3753036    -1.68   0.094    -1.364349    .1068136
          hc15_pnc |   -.118608   .4239631    -0.28   0.780    -.9495605    .7123445
        hosp15_pnc |   .2732141   .3137124     0.87   0.384    -.3416509     .888079
                   |
          religion |
   other christian  |   .4941456   .5300516     0.93   0.351    -.5447364    1.533028
muslim/no religion  |   .8285765   .6046182     1.37   0.171    -.3564533    2.013606
                   |
              v024 |
    central region  |    -.55826   .4864936    -1.15   0.251     -1.51177    .3952499
```

```
  southern region  |  -.6414323   .4718849    -1.36   0.174     -1.56631    .2834452
                   |
           seasons |
 winter-dry season |  -1.332065   .4525548    -2.94   0.003    -2.219056   -.4450736
    hot-dry season |  -.3132541   .4597731    -0.68   0.496    -1.214393    .5878847
                   |
         1.tvradio |   .7959497    .301701     2.64   0.008     .2046266    1.387273
    1.moneyfortreat |  -.1840089   .3102261    -0.59   0.553    -.7920409     .424023
                   |
               age |
             25-34 |   .2444646   .4346326     0.56   0.574    -.6073996    1.096329
             35-49 |   .1984492   .6546376     0.30   0.762    -1.084617    1.481515
                   |
         education |
           primary |   .7201757   .4817787     1.49   0.135    -.2240933    1.664445
         secondary |   .0292842   1.066234     0.03   0.978    -2.060497    2.119065
                   |
      1.employment |   .8254441   .3354727     2.46   0.014     .1679298    1.482958
                   |
       wealthrural |
                 2 |  -1.526517   .5020796    -3.04   0.002    -2.510575    -.542459
                 3 |  -.1963428   .3349082    -0.59   0.558    -.8527508    .4600653
                 4 |  -.8906297   .4562048    -1.95   0.051    -1.784775    .0035152
                 5 |  -1.688937   .6698198    -2.52   0.012     -3.00176   -.3761145
                   |
          birthnum |
               2-3 |  -.8895553   .6184754    -1.44   0.150    -2.101745    .3226342
                4+ |  -.5621694   .6920565    -0.81   0.417    -1.918575    .7942364
                   |
             nsize |
                 1 |  -1.121641   .5508621    -2.04   0.042    -2.201311   -.0419714
                 2 |   -.421961   .3735788    -1.13   0.259    -1.154162    .3102401
                 4 |   -1.22689    .441614    -2.78   0.005    -2.092438   -.3613426
                 5 |  -1.030602   .9222433    -1.12   0.264    -2.838166    .7769618
                   |
              nsex |
            female |   .3729756   .3610229     1.03   0.302    -.3346163    1.080567
             _cons |  -1.916846   .7593233    -2.52   0.012    -3.405092   -.4285996
-------------------------------------------------------------------------------------

. margins, dydx(*)

Average marginal effects                        Number of obs     =        665
Model VCE    : Robust

Expression   : Pr(binarympnc1 != 0), predict()
dy/dx w.r.t. : clinic5_pnc hc5_pnc hosp5_pnc clinic10_pnc hc10_pnc hosp10_pnc clinic15_pnc
hc15_pnc hosp15_pnc
               2.religion 3.religion 2.v024 3.v024 2.seasons 3.seasons 1.tvradio
1.moneyfortreat 2.age 3.age
               2.education 3.education 1.employment 2.wealthrural 3.wealthrural
4.wealthrural 5.wealthrural
               2.birthnum 3.birthnum 1.nsize 2.nsize 4.nsize 5.nsize 1.nsex

-------------------------------------------------------------------------------------
```

	dy/dx	Delta-method Std. Err.	z	P>|z|	[95% Conf.	Interval]
clinic5_pnc	.0463535	.0701009	0.66	0.508	-.0910418	.1837487
hc5_pnc	.0790422	.0298772	2.65	0.008	.0204839	.1376005
hosp5_pnc	.02777	.0409978	0.68	0.498	-.0525841	.1081242
clinic10_pnc	-.0319318	.0398618	-0.80	0.423	-.1100594	.0461958
hc10_pnc	.0386798	.0340779	1.14	0.256	-.0281117	.1054713
hosp10_pnc	.0623555	.0320228	1.95	0.052	-.000408	.1251189
clinic15_pnc	-.061919	.0368261	-1.68	0.093	-.1340968	.0102588
hc15_pnc	-.0116801	.0417035	-0.28	0.779	-.0934174	.0700572
hosp15_pnc	.0269052	.0308744	0.87	0.384	-.0336075	.0874179
religion						
other christian	.0424677	.0407886	1.04	0.298	-.0374765	.1224118
muslim/no religion	.078621	.0548479	1.43	0.152	-.028879	.1861209
v024						
central region	-.0631866	.059939	-1.05	0.292	-.1806649	.0542918
southern region	-.0711233	.0578177	-1.23	0.219	-.1844438	.0421973
seasons						
winter-dry season	-.1121726	.0334712	-3.35	0.001	-.1777749	-.0465703
hot-dry season	-.0352254	.0499601	-0.71	0.481	-.1331455	.0626946
1.tvradio	.0854463	.034421	2.48	0.013	.0179823	.1529102
1.moneyfortreat	-.0183546	.031387	-0.58	0.559	-.0798719	.0431627
age						
25-34	.0235988	.0407565	0.58	0.563	-.0562825	.1034801
35-49	.0189112	.0623361	0.30	0.762	-.1032654	.1410878
education						
primary	.0812092	.0602208	1.35	0.177	-.0368215	.1992398
secondary	.0027447	.1007305	0.03	0.978	-.1946834	.2001729
1.employment	.0732765	.0269904	2.71	0.007	.0203762	.1261768
wealthrural						
2	-.1384912	.0438232	-3.16	0.002	-.224383	-.0525993
3	-.0251305	.0428283	-0.59	0.557	-.1090725	.0588115
4	-.0958224	.0479024	-2.00	0.045	-.1897094	-.0019355
5	-.1466126	.0477798	-3.07	0.002	-.2402592	-.0529659
birthnum						
2-3	-.0949707	.0757243	-1.25	0.210	-.2433877	.0534462
4+	-.065181	.0856991	-0.76	0.447	-.2331483	.1027862
nsize						
1	-.1030533	.0401706	-2.57	0.010	-.1817863	-.0243204
2	-.0470461	.0394563	-1.19	0.233	-.1243791	.0302869
4	-.1093896	.0333361	-3.28	0.001	-.1747271	-.0440521
5	-.0971686	.0668124	-1.45	0.146	-.2281185	.0337813
nsex						

```
           female |   .0365905   .0355701     1.03   0.304    -.0331256    .1063065
------------------------------------------------------------------------------------
Note: dy/dx for factor levels is the discrete change from the base level.

.
. xtgee binarympnc7 clinic5_pnc hc5_pnc hosp5_pnc ///
> clinic10_pnc hc10_pnc hosp10_pnc ///
> clinic15_pnc hc15_pnc hosp15_pnc ///
> i.religion i.v024 i.seasons i.tvradio i.moneyfortreat i.age i.education i.employment
i.wealthrural ///
> i.birthnum ib3.nsize i.nsex [pweight=weight] if residence == 1 & del == 0,
family(binomial) link(logit) corr(exchange
> able)

Iteration 1: tolerance = .02825241
Iteration 2: tolerance = .00101973
Iteration 3: tolerance = .0000304
Iteration 4: tolerance = 7.726e-07

GEE population-averaged model                   Number of obs     =        665
Group variable:                   DHSCLUST      Number of groups  =        323
Link:                                logit      Obs per group:
Family:                           binomial                    min =          1
Correlation:                  exchangeable                    avg =        2.2
                                                              max =         16
                                                Wald chi2(33)     =      64.42
Scale parameter:                         1      Prob > chi2       =     0.0009

                               (Std. Err. adjusted for clustering on DHSCLUST)
------------------------------------------------------------------------------------
                    |               Robust
        binarympnc7 |      Coef.   Std. Err.      z    P>|z|     [95% Conf. Interval]
--------------------+---------------------------------------------------------------
        clinic5_pnc |    1.00931   .5667593     1.78   0.075    -.1015178    2.120138
            hc5_pnc |   .5973067   .2608474     2.29   0.022     .0860553    1.108558
          hosp5_pnc |  -.0202632   .3515284    -0.06   0.954    -.7092462    .6687198
       clinic10_pnc |  -.0267117   .3020294    -0.09   0.930    -.6186784     .565255
           hc10_pnc |   .2263657   .2656361     0.85   0.394    -.2942715    .7470029
         hosp10_pnc |   .5949827   .2624962     2.27   0.023     .0804996    1.109466
       clinic15_pnc |  -.6014747    .320047    -1.88   0.060    -1.228755    .0258058
           hc15_pnc |  -.2869005   .3500907    -0.82   0.412    -.9730657    .3992646
         hosp15_pnc |   .2040824   .2774849     0.74   0.462     -.339778    .7479429
                    |
           religion |
   other christian  |   .3825093   .4474062     0.85   0.393    -.4943907    1.259409
muslim/no religion  |   .5234823   .5112457     1.02   0.306    -.4785409    1.525506
                    |
               v024 |
    central region  |  -.1953452   .3933448    -0.50   0.619    -.9662869    .5755965
   southern region  |  -.3563546   .3924408    -0.91   0.364    -1.125524    .4128152
                    |
            seasons |
 winter-dry season  |  -.8471269   .3700681    -2.29   0.022    -1.572447   -.1218067
    hot-dry season  |  -.2109849   .4248897    -0.50   0.619    -1.043753    .6217836
                    |
```

```
       1.tvradio |   .5008427   .2895213     1.73   0.084    -.0666086    1.068294
  1.moneyfortreat |  -.0125223     .29192    -0.04   0.966    -.5846749    .5596303
                 |
             age |
          25-34  |  -.2652418   .4468683    -0.59   0.553    -1.141088     .610604
          35-49  |  -.1933088   .6489012    -0.30   0.766    -1.465132    1.078514
                 |
       education |
        primary  |   .9173724   .4688732     1.96   0.050    -.0016022    1.836347
      secondary  |  -.1992835   1.081994    -0.18   0.854    -2.319952    1.921385
                 |
    1.employment |   .6734123   .2860806     2.35   0.019     .1127046     1.23412
                 |
     wealthrural |
              2  |  -1.122068   .4925209    -2.28   0.023    -2.087391   -.1567443
              3  |  -.2063112   .3153584    -0.65   0.513    -.8244022    .4117799
              4  |  -.4460411   .4754475    -0.94   0.348    -1.377901    .4858188
              5  |  -1.392511   .6375361    -2.18   0.029    -2.642059   -.1429632
                 |
        birthnum |
            2-3  |  -.8550065   .5083055    -1.68   0.093    -1.851267     .141254
             4+  |  -.5012485   .6221933    -0.81   0.420    -1.720725    .7182279
                 |
           nsize |
              1  |   .0087965   .5496981     0.02   0.987    -1.068592    1.086185
              2  |  -.1244619   .3548941    -0.35   0.726    -.8200415    .5711177
              4  |  -.5859666   .4075507    -1.44   0.150    -1.384751     .212818
              5  |   -.804134   .8030922    -1.00   0.317    -2.378166    .7698978
                 |
            nsex |
         female  |   .4603227   .3466905     1.33   0.184    -.2191782    1.139824
          _cons  |  -1.637093   .7022231    -2.33   0.020    -3.013425   -.2607612
------------------------------------------------------------------------------------

. margins, dydx(*)

Average marginal effects                          Number of obs     =        665
Model VCE    : Robust

Expression   : Pr(binarympnc7 != 0), predict()
dy/dx w.r.t. : clinic5_pnc hc5_pnc hosp5_pnc clinic10_pnc hc10_pnc hosp10_pnc clinic15_pnc
hc15_pnc hosp15_pnc
               2.religion 3.religion 2.v024 3.v024 2.seasons 3.seasons 1.tvradio
1.moneyfortreat 2.age 3.age
               2.education 3.education 1.employment 2.wealthrural 3.wealthrural
4.wealthrural 5.wealthrural
               2.birthnum 3.birthnum 1.nsize 2.nsize 4.nsize 5.nsize 1.nsex

------------------------------------------------------------------------------------
                 |            Delta-method
                 |      dy/dx   Std. Err.      z    P>|z|     [95% Conf. Interval]
-----------------+------------------------------------------------------------------
     clinic5_pnc |   .1238675   .0691345     1.79   0.073    -.0116336    .2593686
         hc5_pnc |   .0733044   .0312343     2.35   0.019     .0120862    .1345226
       hosp5_pnc |  -.0024868   .0431736    -0.06   0.954    -.0871055    .0821319
```

```
      clinic10_pnc |  -.0032782   .0370634    -0.09   0.930     -.075921    .0693647
          hc10_pnc |   .0277807   .0329104     0.84   0.399    -.0367224    .0922838
        hosp10_pnc |   .0730192   .0313325     2.33   0.020     .0116087    .1344297
      clinic15_pnc |  -.0738159   .0389578    -1.89   0.058    -.1501718    .0025399
          hc15_pnc |  -.0352098    .042893    -0.82   0.412    -.1192786    .0488589
        hosp15_pnc |    .025046   .0340059     0.74   0.461    -.0416043    .0916963
                   |
          religion |
  other christian  |   .0428055   .0460461     0.93   0.353    -.0474432    .1330542
muslim/no religion |   .0610335   .0573495     1.06   0.287    -.0513695    .1734365
                   |
              v024 |
   central region  |  -.0258924   .0535342    -0.48   0.629    -.1308175    .0790326
  southern region  |   -.045293   .0523167    -0.87   0.387    -.1478319    .0572459
                   |
           seasons |
 winter-dry season |  -.0950454   .0395805    -2.40   0.016    -.1726217   -.0174691
   hot-dry season  |  -.0282237   .0555823    -0.51   0.612    -.1371631    .0807156
                   |
         1.tvradio |   .0647045   .0386997     1.67   0.095    -.0111456    .1405545
   1.moneyfortreat |  -.0015382   .0358966    -0.04   0.966    -.0718942    .0688179
                   |
               age |
            25-34  |  -.0330957   .0572277    -0.58   0.563      -.14526    .0790686
            35-49  |  -.0245868   .0826045    -0.30   0.766    -.1864887    .1373151
                   |
         education |
          primary  |   .1325103   .0745811     1.78   0.076    -.0136659    .2786866
        secondary  |   -.021331   .1092472    -0.20   0.845    -.2354515    .1927895
                   |
      1.employment |   .0760247   .0292971     2.59   0.009     .0186034    .1334459
                   |
       wealthrural |
                2  |  -.1289874   .0533877    -2.42   0.016    -.2336254   -.0243494
                3  |  -.0302212   .0461369    -0.66   0.512    -.1206479    .0602055
                4  |  -.0615384   .0633975    -0.97   0.332    -.1857953    .0627184
                5  |  -.1483542   .0562482    -2.64   0.008    -.2585985   -.0381098
                   |
          birthnum |
              2-3  |  -.1118726   .0755113    -1.48   0.138    -.2598719    .0361268
               4+  |  -.0716714   .0932664    -0.77   0.442    -.2544701    .1111273
                   |
             nsize |
                1  |   .0011801   .0738603     0.02   0.987    -.1435835    .1459436
                2  |  -.0161292   .0452948    -0.36   0.722    -.1049053     .072647
                4  |  -.0668912   .0430646    -1.55   0.120    -.1512961    .0175138
                5  |  -.0862153   .0708071    -1.22   0.223    -.2249946     .052564
                   |
              nsex |
           female  |   .0562366   .0421036     1.34   0.182    -.0262849    .1387581
--------------------------------------------------------------------------------------
Note: dy/dx for factor levels is the discrete change from the base level.

.
. xtgee binarypnc1 clinic5_pnc hc5_pnc hosp5_pnc ///
```

```
> clinic10_pnc hc10_pnc hosp10_pnc ///
> clinic15_pnc hc15_pnc hosp15_pnc ///
> i.religion i.v024 i.seasons i.tvradio i.moneyfortreat i.age i.education i.employment
i.wealthrural ///
> i.birthnum ib3.nsize i.nsex [pweight=weight] if residence == 1 & del == 0,
family(binomial) link(logit) corr(exchange
> able)

Iteration 1: tolerance = .00339711
Iteration 2: tolerance = .00033524
Iteration 3: tolerance = .00003309
Iteration 4: tolerance = 3.287e-06
Iteration 5: tolerance = 3.264e-07

GEE population-averaged model                   Number of obs      =       664
Group variable:                   DHSCLUST      Number of groups   =       323
Link:                                logit      Obs per group:
Family:                           binomial                    min =          1
Correlation:                  exchangeable                    avg =        2.2
                                                              max =         16
                                                Wald chi2(33)      =     52.42
Scale parameter:                         1      Prob > chi2        =    0.0172

                              (Std. Err. adjusted for clustering on DHSCLUST)
------------------------------------------------------------------------------------
                   |               Robust
       binarypnc1 |      Coef.   Std. Err.      z    P>|z|     [95% Conf. Interval]
--------------------+---------------------------------------------------------------
       clinic5_pnc |  -.3299759   .6804758    -0.48   0.628    -1.663684    1.003732
           hc5_pnc |   .6330333    .309911     2.04   0.041      .025619    1.240448
         hosp5_pnc |   1.281857   .5954893     2.15   0.031     .1147196    2.448995
      clinic10_pnc |   .2811966   .3035996     0.93   0.354    -.3138476    .8762408
          hc10_pnc |   .2585695   .3384186     0.76   0.445    -.4047187    .9218577
        hosp10_pnc |    .279462   .2854076     0.98   0.327    -.2799266    .8388507
      clinic15_pnc |  -.0797706   .3282192    -0.24   0.808    -.7230683    .5635271
          hc15_pnc |    .463424   .4176652     1.11   0.267    -.3551847    1.282033
        hosp15_pnc |  -.0004361   .2815351    -0.00   0.999    -.5522348    .5513626
                   |
          religion |
   other christian  |   .3412232   .4220518     0.81   0.419    -.4859831     1.16843
muslim/no religion  |   .6752065   .5341529     1.26   0.206     -.371714    1.722127
                   |
              v024 |
    central region  |  -.8252024   .4463231    -1.85   0.064     -1.69998    .0495747
   southern region  |  -1.459214   .5446553    -2.68   0.007    -2.526719   -.3917089
                   |
           seasons |
 winter-dry season  |  -.5327801   .3652164    -1.46   0.145    -1.248591    .1830308
   hot-dry season   |  -.0198342   .3606663    -0.05   0.956    -.7267272    .6870587
                   |
         1.tvradio |    .321764   .2791411     1.15   0.249    -.2253426    .8688706
   1.moneyfortreat |   .1988449   .2973275     0.67   0.504    -.3839063    .7815961
                   |
               age |
            25-34  |    .971951   .4051647     2.40   0.016     .1778428    1.766059
```

```
           35-49 |   .6606348   .5999292     1.10   0.271    -.5152047    1.836474
                 |
       education |
         primary |  -.1658148    .361766    -0.46   0.647    -.8748632    .5432335
       secondary |  -1.356685   1.185169    -1.14   0.252    -3.679573    .9662041
                 |
    1.employment |   .5769555   .2986374     1.93   0.053    -.0083631    1.162274
                 |
     wealthrural |
               2 |   .8108986   .4294754     1.89   0.059    -.0308578    1.652655
               3 |   1.004273   .4619151     2.17   0.030      .098936     1.90961
               4 |   .6968589   .4894406     1.42   0.155     -.262427    1.656145
               5 |   .8058767   .4944287     1.63   0.103    -.1631858    1.774939
                 |
        birthnum |
             2-3 |  -.8980414   .5230306    -1.72   0.086    -1.923162    .1270797
              4+ |  -1.340815   .6805854    -1.97   0.049    -2.674737   -.0068917
                 |
           nsize |
               1 |   .7032468   .4569255     1.54   0.124    -.1923108    1.598804
               2 |   .2783255   .3096015     0.90   0.369    -.3284824    .8851333
               4 |  -.4559749   .4097816    -1.11   0.266    -1.259132    .3471823
               5 |   -1.14677   .7867252    -1.46   0.145    -2.688723    .3951828
                 |
            nsex |
          female |   .0029544   .3214227     0.01   0.993    -.6270225    .6329314
           _cons |  -3.086854   .7794132    -3.96   0.000    -4.614476   -1.559232
------------------------------------------------------------------------------------

. margins, dydx(*)

Average marginal effects                        Number of obs     =        664
Model VCE    : Robust

Expression   : Pr(binarypnc1 != 0), predict()
dy/dx w.r.t. : clinic5_pnc hc5_pnc hosp5_pnc clinic10_pnc hc10_pnc hosp10_pnc clinic15_pnc
hc15_pnc hosp15_pnc
               2.religion 3.religion 2.v024 3.v024 2.seasons 3.seasons 1.tvradio
1.moneyfortreat 2.age 3.age
               2.education 3.education 1.employment 2.wealthrural 3.wealthrural
4.wealthrural 5.wealthrural
               2.birthnum 3.birthnum 1.nsize 2.nsize 4.nsize 5.nsize 1.nsex

------------------------------------------------------------------------------------
                 |            Delta-method
                 |      dy/dx   Std. Err.      z    P>|z|     [95% Conf. Interval]
-----------------+------------------------------------------------------------------
     clinic5_pnc |  -.0353045   .0731159    -0.48   0.629    -.1786091        .108
         hc5_pnc |    .067729   .0329923     2.05   0.040     .0030653    .1323927
       hosp5_pnc |   .1371475   .0639688     2.14   0.032     .0117709    .2625241
    clinic10_pnc |   .0300856   .0327805     0.92   0.359     -.034163    .0943342
        hc10_pnc |   .0276647   .0362777     0.76   0.446    -.0434382    .0987676
      hosp10_pnc |      .0299   .0301317     0.99   0.321     -.029157     .088957
    clinic15_pnc |  -.0085348   .0351254    -0.24   0.808    -.0773792    .0603097
        hc15_pnc |   .0495823   .0449202     1.10   0.270    -.0384597    .1376243
```

```
        hosp15_pnc |  -.0000467   .0301222    -0.00   0.999    -.0590851    .0589918
                   |
          religion |
   other christian |   .0328173   .0378534     0.87   0.386     -.041374    .1070086
 muslim/no religion |   .0719507   .0578304     1.24   0.213    -.0413948    .1852962
                   |
              v024 |
    central region |  -.1192778    .072196    -1.65   0.099    -.2607794    .0222238
   southern region |  -.1807753   .0769364    -2.35   0.019    -.3315678   -.0299828
                   |
           seasons |
  winter-dry season |  -.0527982   .0345377    -1.53   0.126    -.1204909    .0148946
    hot-dry season |  -.0022862   .0414547    -0.06   0.956    -.0835358    .0789635
                   |
         1.tvradio |   .0353297   .0316582     1.12   0.264    -.0267191    .0973785
   1.moneyfortreat |   .0210317   .0313283     0.67   0.502    -.0403707     .082434
                   |
               age |
             25-34 |   .0980925   .0377533     2.60   0.009     .0240973    .1720877
             35-49 |   .0602945   .0558421     1.08   0.280    -.0491541    .1697431
                   |
         education |
           primary |  -.0172492   .0364992    -0.47   0.637    -.0887862    .0542879
         secondary |  -.0976417   .0522117    -1.87   0.061    -.1999747    .0046914
                   |
      1.employment |   .0571247   .0275996     2.07   0.038     .0030305    .1112189
                   |
       wealthrural |
                 2 |   .0768889   .0417739     1.84   0.066    -.0049863    .1587642
                 3 |   .1015186   .0469477     2.16   0.031     .0095028    .1935344
                 4 |   .0635574   .0459539     1.38   0.167    -.0265105    .1536253
                 5 |   .0762834   .0502049     1.52   0.129    -.0221165    .1746832
                   |
          birthnum |
               2-3 |  -.1288339   .0846712    -1.52   0.128    -.2947864    .0371186
                4+ |  -.1742369    .100202    -1.74   0.082    -.3706293    .0221554
                   |
             nsize |
                 1 |   .0909331   .0662216     1.37   0.170    -.0388588    .2207251
                 2 |   .0319564   .0363939     0.88   0.380    -.0393742    .1032871
                 4 |  -.0416719    .034184    -1.22   0.223    -.1086713    .0253275
                 5 |  -.0836829   .0401763    -2.08   0.037     -.162427   -.0049388
                   |
              nsex |
            female |   .0003161   .0343889     0.01   0.993    -.0670849     .067717
------------------------------------------------------------------------------------
Note: dy/dx for factor levels is the discrete change from the base level.

.
. xtgee binarypnc7 clinic5_pnc hc5_pnc hosp5_pnc ///
> clinic10_pnc hc10_pnc hosp10_pnc ///
> clinic15_pnc hc15_pnc hosp15_pnc ///
> i.religion i.v024 i.seasons i.tvradio i.moneyfortreat i.age i.education i.employment
i.wealthrural ///
> i.birthnum ib3.nsize i.nsex [pweight=weight] if residence == 1 & del == 0,
```

```
family(binomial) link(logit) corr(exchange
> able)

Iteration 1: tolerance = .02097978
Iteration 2: tolerance = .00321883
Iteration 3: tolerance = .00051196
Iteration 4: tolerance = .00007985
Iteration 5: tolerance = .00001242
Iteration 6: tolerance = 1.932e-06
Iteration 7: tolerance = 3.004e-07

GEE population-averaged model                   Number of obs     =        664
Group variable:                   DHSCLUST      Number of groups  =        323
Link:                                logit      Obs per group:
Family:                           binomial                   min =          1
Correlation:                  exchangeable                   avg =        2.2
                                                             max =         16
                                                Wald chi2(33)     =      31.48
Scale parameter:                         1      Prob > chi2       =     0.5428

                                (Std. Err. adjusted for clustering on DHSCLUST)
-------------------------------------------------------------------------------------
                   |               Robust
       binarypnc7 |      Coef.   Std. Err.      z    P>|z|     [95% Conf. Interval]
-------------------+-----------------------------------------------------------------
       clinic5_pnc |    .232669    .508231     0.46   0.647    -.7634455    1.228783
          hc5_pnc |   .3502889   .2356009     1.49   0.137    -.1114804    .8120581
        hosp5_pnc |   .6490876   .4827631     1.34   0.179    -.2971106    1.595286
      clinic10_pnc |   .2030623   .2296658     0.88   0.377    -.2470744     .653199
         hc10_pnc |   .2278533   .2237012     1.02   0.308     -.210593    .6662997
       hosp10_pnc |   .3677732    .233187     1.58   0.115    -.0892649    .8248112
      clinic15_pnc |   .0316646   .2468305     0.13   0.898    -.4521144    .5154435
         hc15_pnc |   -.113329   .2914793    -0.39   0.697     -.684618    .4579599
       hosp15_pnc |   .0471521   .2275592     0.21   0.836    -.3988557    .4931599
                   |
          religion |
   other christian  |   .0547708   .3181011     0.17   0.863    -.5686959    .6782374
muslim/no religion  |  -.0534644   .4191666    -0.13   0.899    -.8750158    .7680871
                   |
              v024 |
    central region  |  -.2863002   .3424688    -0.84   0.403    -.9575268    .3849263
   southern region  |  -.5566088   .3511414    -1.59   0.113    -1.244833    .1316156
                   |
           seasons |
  winter-dry season |  -.2155004   .3129236    -0.69   0.491    -.8288194    .3978187
     hot-dry season |  -.2865772   .2899591    -0.99   0.323    -.8548867    .2817322
                   |
         1.tvradio |   .0582351   .2361008     0.25   0.805    -.4045138    .5209841
    1.moneyfortreat |   .2234051   .2219423     1.01   0.314    -.2115937     .658404
                   |
               age |
             25-34  |   .3628272   .3537478     1.03   0.305    -.3305057     1.05616
             35-49  |   .2912182   .4216819     0.69   0.490    -.5352632      1.1177
                   |
         education |
```

```
         primary |   .0550993   .3402926     0.16   0.871     -.611862    .7220606
       secondary |  -1.436815   .9113864    -1.58   0.115    -3.223099    .3494697
                 |
    1.employment |    .242116   .2611684     0.93   0.354    -.2697646    .7539965
                 |
     wealthrural |
              2  |   .6947144   .3141041     2.21   0.027     .0790816    1.310347
              3  |   .6825567   .3458666     1.97   0.048     .0046707    1.360443
              4  |    .644895   .3486778     1.85   0.064    -.0385009    1.328291
              5  |   .3853126    .410521     0.94   0.348    -.4192938    1.189919
                 |
        birthnum |
            2-3  |  -.3979562   .4197863    -0.95   0.343    -1.220722    .4248097
             4+  |  -.7196862   .5204889    -1.38   0.167    -1.739826    .3004532
                 |
           nsize |
              1  |   .6718485   .4165592     1.61   0.107    -.1445925    1.488289
              2  |   .0948613   .2506291     0.38   0.705    -.3963627    .5860853
              4  |   .0854867   .3094175     0.28   0.782    -.5209605     .691934
              5  |   .0874346   .5739195     0.15   0.879    -1.037427    1.212296
                 |
            nsex |
         female  |   .1471317   .2561545     0.57   0.566    -.3549219    .6491853
           _cons |  -1.751448   .5621673    -3.12   0.002    -2.853276   -.6496205
------------------------------------------------------------------------------------

. margins, dydx(*)

Average marginal effects                        Number of obs     =        664
Model VCE    : Robust

Expression   : Pr(binarypnc7 != 0), predict()
dy/dx w.r.t. : clinic5_pnc hc5_pnc hosp5_pnc clinic10_pnc hc10_pnc hosp10_pnc clinic15_pnc
hc15_pnc hosp15_pnc
               2.religion 3.religion 2.v024 3.v024 2.seasons 3.seasons 1.tvradio
1.moneyfortreat 2.age 3.age
               2.education 3.education 1.employment 2.wealthrural 3.wealthrural
4.wealthrural 5.wealthrural
               2.birthnum 3.birthnum 1.nsize 2.nsize 4.nsize 5.nsize 1.nsex

------------------------------------------------------------------------------------
                 |            Delta-method
                 |      dy/dx   Std. Err.      z    P>|z|     [95% Conf. Interval]
-----------------+------------------------------------------------------------------
     clinic5_pnc |   .0419702    .091551     0.46   0.647    -.1374665    .2214069
         hc5_pnc |   .0631872   .0423877     1.49   0.136    -.0198911    .1462655
       hosp5_pnc |   .1170863   .0866478     1.35   0.177    -.0527403    .2869128
    clinic10_pnc |   .0366296   .0415376     0.88   0.378    -.0447825    .1180417
        hc10_pnc |   .0411015    .040687     1.01   0.312    -.0386436    .1208467
      hosp10_pnc |   .0663411   .0416452     1.59   0.111     -.015282    .1479642
    clinic15_pnc |   .0057118   .0445303     0.13   0.898     -.081566    .0929896
        hc15_pnc |   -.020443   .0524452    -0.39   0.697    -.1232336    .0823476
      hosp15_pnc |   .0085056   .0409828     0.21   0.836    -.0718192    .0888304
                 |
        religion |
```

```
   other christian  |   .0098707   .0568038     0.17   0.862    -.1014627    .1212042
muslim/no religion  |   -.009406   .0737523    -0.13   0.899    -.1539577    .1351458
                    |
               v024 |
    central region  |  -.0565169   .0697171    -0.81   0.418    -.1931599    .0801261
   southern region  |  -.1041441   .0695006    -1.50   0.134    -.2403628    .0320746
                    |
            seasons |
 winter-dry season  |  -.0389777    .055891    -0.70   0.486     -.148522    .0705667
    hot-dry season  |  -.0510192   .0499068    -1.02   0.307    -.1488348    .0467964
                    |
          1.tvradio |   .0105505   .0430196     0.25   0.806    -.0737663    .0948674
    1.moneyfortreat |   .0398575   .0394541     1.01   0.312    -.0374711    .1171861
                    |
                age |
             25-34  |   .0640403   .0611159     1.05   0.295    -.0557446    .1838252
             35-49  |   .0505855   .0731854     0.69   0.489    -.0928553    .1940263
                    |
          education |
           primary  |   .0100748   .0626735     0.16   0.872    -.1127631    .1329127
         secondary  |  -.1776585    .069005    -2.57   0.010    -.3129059   -.0424111
                    |
       1.employment |   .0426836   .0446857     0.96   0.339    -.0448987     .130266
                    |
        wealthrural |
                 2  |   .1211664    .055411     2.19   0.029     .0125629      .22977
                 3  |   .1187122   .0606635     1.96   0.050    -.0001861    .2376105
                 4  |   .1111784     .06073     1.83   0.067    -.0078502     .230207
                 5  |   .0622487   .0684669     0.91   0.363     -.071944    .1964413
                    |
           birthnum |
               2-3  |  -.0806557   .0873229    -0.92   0.356    -.2518055    .0904941
                4+  |  -.1376385    .103295    -1.33   0.183     -.340093    .0648161
                    |
              nsize |
                 1  |   .1333436   .0872649     1.53   0.127    -.0376924    .3043796
                 2  |   .0168114   .0445729     0.38   0.706    -.0705498    .1041727
                 4  |   .0151176   .0551965     0.27   0.784    -.0930655    .1233007
                 5  |    .015469   .1029773     0.15   0.881    -.1863629    .2173008
                    |
               nsex |
            female  |   .0265077   .0461498     0.57   0.566    -.0639442    .1169595
------------------------------------------------------------------------------------
Note: dy/dx for factor levels is the discrete change from the base level.

.
. /******
> Facility
> Delivery
> Only
> *******/
.
. ***Analysis commands
. xtgee specialmpnc7 clinic5_pnc hc5_pnc hosp5_pnc ///
> clinic10_pnc hc10_pnc hosp10_pnc ///
```

```
> clinic15_pnc hc15_pnc hosp15_pnc ///
> i.religion i.mhealthcheckb4 i.v024 i.csect i.seasons i.tvradio i.moneyfortreat i.age
i.education i.employment i.wealt
> hrural ///
> i.birthnum ib3.nsize i.nsex [pweight=weight] if residence == 1 & del == 1,
family(binomial) link(logit) corr(exchange
> able)

Iteration 1: tolerance = .13177446
Iteration 2: tolerance = .01338172
Iteration 3: tolerance = .00072368
Iteration 4: tolerance = .00005913
Iteration 5: tolerance = 3.940e-06
Iteration 6: tolerance = 2.416e-07

GEE population-averaged model                   Number of obs     =     10,083
Group variable:                   DHSCLUST      Number of groups  =        677
Link:                                logit      Obs per group:
Family:                           binomial                    min =          3
Correlation:                  exchangeable                    avg =       14.8
                                                              max =         24
                                                Wald chi2(35)     =     235.38
Scale parameter:                         1      Prob > chi2       =     0.0000

                               (Std. Err. adjusted for clustering on DHSCLUST)
------------------------------------------------------------------------------------
                    |               Robust
       specialmpnc7 |      Coef.   Std. Err.      z    P>|z|     [95% Conf. Interval]
--------------------+---------------------------------------------------------------
         clinic5_pnc |  -.2018192   .2482814    -0.81   0.416    -.6884418    .2848034
             hc5_pnc |   .2813825   .1245716     2.26   0.024     .0372266    .5255384
           hosp5_pnc |   .3063221   .1789242     1.71   0.087    -.0443629    .6570071
        clinic10_pnc |   .0135674   .1475475     0.09   0.927    -.2756203    .3027551
            hc10_pnc |   .3925898   .1354864     2.90   0.004     .1270413    .6581383
          hosp10_pnc |   -.370336   .1374954    -2.69   0.007     -.639822   -.1008501
        clinic15_pnc |  -.0338633    .145728    -0.23   0.816     -.319485    .2517584
            hc15_pnc |   .0646442   .1545474     0.42   0.676    -.2382632    .3675515
          hosp15_pnc |  -.0155924   .1308651    -0.12   0.905    -.2720833    .2408985
                    |
            religion |
     other christian  |  -.1220516   .0941086    -1.30   0.195    -.3065011    .0623979
muslim/no religion   |  -.1305404   .1362952    -0.96   0.338    -.3976742    .1365933
                    |
    1.mhealthcheckb4 |   .8894639   .0766105    11.61   0.000     .7393101    1.039618
                    |
                v024 |
      central region  |  -.6150741   .1618606    -3.80   0.000    -.9323151   -.2978332
     southern region  |  -.3254431   .1626418    -2.00   0.045    -.6442151   -.0066711
                    |
             1.csect |   .1664578   .1314079     1.27   0.205     -.091097    .4240126
                    |
             seasons |
   winter-dry season  |   .0013552    .070734     0.02   0.985    -.1372809    .1399912
      hot-dry season  |   .0517279    .091918     0.56   0.574    -.1284281    .2318839
                    |
```

```
         1.tvradio |  -.0053402   .0835728    -0.06   0.949    -.1691399    .1584594
   1.moneyfortreat |   .0646943    .087644     0.74   0.460    -.1070849    .2364734
                   |
               age |
            25-34  |   .1062255   .0969174     1.10   0.273    -.0837291      .29618
            35-49  |   .2019327   .1236915     1.63   0.103    -.0404982    .4443636
                   |
         education |
          primary  |   .0776962   .0899057     0.86   0.387    -.0985157     .253908
        secondary  |   .3828745    .155203     2.47   0.014     .0786823    .6870667
                   |
      1.employment |   .2704895   .0841596     3.21   0.001     .1055397    .4354394
                   |
       wealthrural |
                2  |   .0669983    .102315     0.65   0.513    -.1335353    .2675319
                3  |   .0911871   .0965416     0.94   0.345    -.0980311    .2804052
                4  |   .1314842   .1147275     1.15   0.252    -.0933776    .3563461
                5  |   .2050108   .1366364     1.50   0.134    -.0627917    .4728133
                   |
          birthnum |
              2-3  |   .1983242   .1134783     1.75   0.081    -.0240893    .4207376
               4+  |   .1090067    .133595     0.82   0.415    -.1528346    .3708481
                   |
             nsize |
                1  |   .2033355   .1236607     1.64   0.100     -.039035     .445706
                2  |   .0866715    .097192     0.89   0.373    -.1038214    .2771644
                4  |   .0132485   .1131023     0.12   0.907    -.2084279    .2349248
                5  |  -.3980751   .2137418    -1.86   0.063    -.8170014    .0208512
                   |
              nsex |
           female  |   .0298801   .0683604     0.44   0.662    -.1041038     .163864
            _cons  |  -2.896363   .2448884   -11.83   0.000    -3.376336   -2.416391
------------------------------------------------------------------------------------

. margins, dydx(*)

Average marginal effects                        Number of obs     =     10,083
Model VCE    : Robust

Expression   : Pr(specialmpnc7 != 0), predict()
dy/dx w.r.t. : clinic5_pnc hc5_pnc hosp5_pnc clinic10_pnc hc10_pnc hosp10_pnc clinic15_pnc
hc15_pnc hosp15_pnc
               2.religion 3.religion 1.mhealthcheckb4 2.v024 3.v024 1.csect 2.seasons
3.seasons 1.tvradio
               1.moneyfortreat 2.age 3.age 2.education 3.education 1.employment
2.wealthrural 3.wealthrural
               4.wealthrural 5.wealthrural 2.birthnum 3.birthnum 1.nsize 2.nsize 4.nsize
5.nsize 1.nsex

------------------------------------------------------------------------------------
                   |            Delta-method
                   |      dy/dx   Std. Err.      z    P>|z|     [95% Conf. Interval]
-------------------+----------------------------------------------------------------
       clinic5_pnc |  -.0226583   .0279112    -0.81   0.417    -.0773633    .0320467
           hc5_pnc |   .0315909   .0139608     2.26   0.024     .0042283    .0589535
```

hosp5_pnc	.0343909	.020228	1.70	0.089	-.0052554	.0740371
clinic10_pnc	.0015232	.0165633	0.09	0.927	-.0309402	.0339866
hc10_pnc	.0440762	.015478	2.85	0.004	.0137399	.0744124
hosp10_pnc	-.0415777	.0155871	-2.67	0.008	-.0721279	-.0110275
clinic15_pnc	-.0038018	.0163753	-0.23	0.816	-.0358969	.0282932
hc15_pnc	.0072576	.0173704	0.42	0.676	-.0267877	.0413029
hosp15_pnc	-.0017506	.0146845	-0.12	0.905	-.0305317	.0270306
religion						
other christian	-.0140881	.0111344	-1.27	0.206	-.0359111	.0077348
muslim/no religion	-.0150267	.01559	-0.96	0.335	-.0455826	.0155292
1.mhealthcheckb4	.1000912	.0089712	11.16	0.000	.0825079	.1176745
v024						
central region	-.0736597	.0204574	-3.60	0.000	-.1137555	-.033564
southern region	-.0425934	.0220439	-1.93	0.053	-.0857986	.0006118
1.csect	.0195982	.0161343	1.21	0.224	-.0120245	.0512209
seasons						
winter-dry season	.0001512	.0078899	0.02	0.985	-.0153128	.0156151
hot-dry season	.0058664	.010461	0.56	0.575	-.0146368	.0263696
1.tvradio	-.0005993	.009377	-0.06	0.949	-.0189779	.0177792
1.moneyfortreat	.0072422	.0098441	0.74	0.462	-.0120518	.0265362
age						
25-34	.0116715	.0105368	1.11	0.268	-.0089804	.0323233
35-49	.0229082	.0141157	1.62	0.105	-.004758	.0505743
education						
primary	.0087477	.0102212	0.86	0.392	-.0112854	.0287808
secondary	.0475832	.0213735	2.23	0.026	.0056919	.0894745
1.employment	.0293535	.0087689	3.35	0.001	.0121667	.0465402
wealthrural						
2	.0072291	.0111065	0.65	0.515	-.0145392	.0289973
3	.0099201	.0105132	0.94	0.345	-.0106854	.0305255
4	.0144998	.0127293	1.14	0.255	-.0104491	.0394487
5	.0231716	.0157453	1.47	0.141	-.0076887	.0540319
birthnum						
2-3	.0220105	.0122895	1.79	0.073	-.0020765	.0460974
4+	.0117382	.0143047	0.82	0.412	-.0162986	.0397749
nsize						
1	.0239621	.0151463	1.58	0.114	-.005724	.0536482
2	.0098308	.011179	0.88	0.379	-.0120796	.0317413
4	.0014665	.0125501	0.12	0.907	-.0231312	.0260642
5	-.0382971	.0182128	-2.10	0.035	-.0739935	-.0026007
nsex						
female	.003355	.0077029	0.44	0.663	-.0117423	.0184524

```
------------------------------------------------------------------------------------
Note: dy/dx for factor levels is the discrete change from the base level.

.
. xtgee specialpnc7 clinic5_pnc hc5_pnc hosp5_pnc ///
> clinic10_pnc hc10_pnc hosp10_pnc ///
> clinic15_pnc hc15_pnc hosp15_pnc ///
> i.religion i.newborncheckb4 i.v024 i.csect i.seasons i.tvradio i.moneyfortreat i.age
i.education i.employment i.wealt
> hrural ///
> i.birthnum ib3.nsize i.nsex [pweight=weight] if residence == 1 & del == 1,
family(binomial) link(logit) corr(exchange
> able)

Iteration 1: tolerance = .13836571
Iteration 2: tolerance = .0093803
Iteration 3: tolerance = .00042035
Iteration 4: tolerance = .0000254
Iteration 5: tolerance = 1.554e-06
Iteration 6: tolerance = 9.696e-08

GEE population-averaged model                 Number of obs      =     10,029
Group variable:                   DHSCLUST      Number of groups   =        677
Link:                                logit      Obs per group:
Family:                           binomial                    min =          3
Correlation:                  exchangeable                    avg =       14.7
                                                              max =         24
                                                Wald chi2(35)      =     182.43
Scale parameter:                         1      Prob > chi2        =     0.0000

                                       (Std. Err. adjusted for clustering on DHSCLUST)
------------------------------------------------------------------------------------
                    |               Robust
        specialpnc7 |      Coef.   Std. Err.      z    P>|z|     [95% Conf. Interval]
--------------------+---------------------------------------------------------------
        clinic5_pnc |  -.7629425   .2222593    -3.43   0.001    -1.198563   -.3273224
            hc5_pnc |   .0529879   .0933395     0.57   0.570    -.1299541    .2359299
          hosp5_pnc |   .1163474   .1289903     0.90   0.367    -.1364689    .3691637
       clinic10_pnc |  -.2677441   .1247598    -2.15   0.032    -.5122688   -.0232195
           hc10_pnc |   .3944186   .1034522     3.81   0.000      .191656    .5971812
         hosp10_pnc |  -.0555773   .0957849    -0.58   0.562    -.2433123    .1321577
       clinic15_pnc |   .1688052   .1166041     1.45   0.148    -.0597347    .3973451
           hc15_pnc |   .1730598   .1220307     1.42   0.156     -.066116    .4122356
         hosp15_pnc |  -.0746097   .0925828    -0.81   0.420    -.2560687    .1068494
                    |
           religion |
    other christian |  -.0289484     .07962    -0.36   0.716    -.1850007     .127104
 muslim/no religion |  -.0263034    .129541    -0.20   0.839    -.2801991    .2275923
                    |
   1.newborncheckb4 |   .4668698   .0694398     6.72   0.000     .3307703    .6029694
                    |
               v024 |
     central region |  -.7723978   .1268102    -6.09   0.000    -1.020941   -.5238543
    southern region |  -.6534478   .1307384    -5.00   0.000    -.9096903   -.3972052
                    |
```

```
           1.csect |  -.3543213   .1314997    -2.69   0.007     -.612056   -.0965867
                   |
           seasons |
 winter-dry season  |   .0323141   .0606268     0.53   0.594    -.0865122    .1511404
    hot-dry season  |  -.0449021   .0790919    -0.57   0.570    -.1999194    .1101152
                   |
         1.tvradio |  -.0533907   .0613147    -0.87   0.384    -.1735653    .0667839
    1.moneyfortreat |  -.0036556   .0651307    -0.06   0.955    -.1313094    .1239983
                   |
               age |
             25-34  |  -.0885696   .0870673    -1.02   0.309    -.2592183    .0820791
             35-49  |  -.1900193   .1141527    -1.66   0.096    -.4137544    .0337158
                   |
         education |
           primary  |   .1204106   .0741346     1.62   0.104    -.0248906    .2657118
         secondary  |   .2016687   .1296364     1.56   0.120    -.0524139    .4557513
                   |
      1.employment |   .2046659   .0742683     2.76   0.006     .0591027     .350229
                   |
        wealthrural |
                 2  |   .0571714   .0959108     0.60   0.551    -.1308104    .2451531
                 3  |   .1662652   .0966517     1.72   0.085    -.0231686     .355699
                 4  |   .2105188   .0961416     2.19   0.029     .0220848    .3989528
                 5  |   .2640491   .1021233     2.59   0.010     .0638912     .464207
                   |
          birthnum |
               2-3  |    .191254   .0941017     2.03   0.042     .0068181    .3756899
                4+  |   .2551589   .1110702     2.30   0.022     .0374654    .4728524
                   |
             nsize |
                 1  |  -.0786368   .1092832    -0.72   0.472    -.2928279    .1355543
                 2  |   .1058294   .0671539     1.58   0.115    -.0257899    .2374487
                 4  |   -.092449   .0927593    -1.00   0.319     -.274254    .0893559
                 5  |  -.2600292   .1636699    -1.59   0.112    -.5808163    .0607579
                   |
              nsex |
            female  |  -.0576239   .0502403    -1.15   0.251    -.1560931    .0408452
             _cons  |   -1.57195   .2010945    -7.82   0.000    -1.966088   -1.177812
------------------------------------------------------------------------------------

. margins, dydx(*)

Average marginal effects                        Number of obs     =     10,029
Model VCE    : Robust

Expression   : Pr(specialpnc7 != 0), predict()
dy/dx w.r.t. : clinic5_pnc hc5_pnc hosp5_pnc clinic10_pnc hc10_pnc hosp10_pnc clinic15_pnc
hc15_pnc hosp15_pnc
               2.religion 3.religion 1.newborncheckb4 2.v024 3.v024 1.csect 2.seasons
3.seasons 1.tvradio
               1.moneyfortreat 2.age 3.age 2.education 3.education 1.employment
2.wealthrural 3.wealthrural
               4.wealthrural 5.wealthrural 2.birthnum 3.birthnum 1.nsize 2.nsize 4.nsize
5.nsize 1.nsex
```

	dy/dx	Delta-method Std. Err.	z	P>|z|	[95% Conf.	Interval]
clinic5_pnc	-.13358	.0389629	-3.43	0.001	-.2099459	-.0572141
hc5_pnc	.0092774	.0163105	0.57	0.569	-.0226905	.0412453
hosp5_pnc	.0203707	.0225748	0.90	0.367	-.0238751	.0646165
clinic10_pnc	-.0468781	.0217493	-2.16	0.031	-.0895059	-.0042502
hc10_pnc	.0690569	.0181411	3.81	0.000	.033501	.1046128
hosp10_pnc	-.0097308	.0168011	-0.58	0.562	-.0426604	.0231988
clinic15_pnc	.0295553	.0204348	1.45	0.148	-.0104962	.0696069
hc15_pnc	.0303002	.021387	1.42	0.157	-.0116175	.072218
hosp15_pnc	-.0130631	.0162396	-0.80	0.421	-.0448921	.018766
religion						
other christian	-.0050911	.0140698	-0.36	0.717	-.0326674	.0224853
muslim/no religion	-.0046288	.0227598	-0.20	0.839	-.0492373	.0399796
1.newborncheckb4	.078868	.011404	6.92	0.000	.0565167	.1012194
v024						
central region	-.1507043	.0256906	-5.87	0.000	-.2010569	-.1003516
southern region	-.130667	.0271298	-4.82	0.000	-.1838404	-.0774937
1.csect	-.0572786	.0196147	-2.92	0.003	-.0957227	-.0188345
seasons						
winter-dry season	.0056969	.0106824	0.53	0.594	-.0152402	.0266339
hot-dry season	-.0077696	.0136549	-0.57	0.569	-.0345327	.0189935
1.tvradio	-.0093188	.0106705	-0.87	0.382	-.0302325	.0115949
1.moneyfortreat	-.0006401	.011405	-0.06	0.955	-.0229934	.0217132
age						
25-34	-.0157263	.0155092	-1.01	0.311	-.0461238	.0146713
35-49	-.0329263	.0196485	-1.68	0.094	-.0714367	.0055841
education						
primary	.0213594	.0133482	1.60	0.110	-.0048027	.0475215
secondary	.0364539	.0244533	1.49	0.136	-.0114736	.0843815
1.employment	.0351888	.0125059	2.81	0.005	.0106776	.0596999
wealthrural						
2	.0095358	.0160481	0.59	0.552	-.021918	.0409896
3	.0285107	.0165861	1.72	0.086	-.0039974	.0610189
4	.0364952	.0166591	2.19	0.028	.0038441	.0691463
5	.0463708	.0179773	2.58	0.010	.0111359	.0816058
birthnum						
2-3	.0322408	.0155041	2.08	0.038	.0018533	.0626283
4+	.0437046	.0187039	2.34	0.019	.0070457	.0803636
nsize						
1	-.0135319	.0185949	-0.73	0.467	-.0499773	.0229134

```
                2  |   .0190358   .0121423     1.57   0.117    -.0047626    .0428343
                4  |  -.0158541   .0157256    -1.01   0.313    -.0466758    .0149676
                5  |  -.0427139   .0256007    -1.67   0.095    -.0928903    .0074625
                   |
              nsex |
           female  |  -.0100886   .0087938    -1.15   0.251    -.0273242    .0071469
--------------------------------------------------------------------------------------
Note: dy/dx for factor levels is the discrete change from the base level.

.
. ***Appendix analysis
. xtgee binarympnc1 clinic5_pnc hc5_pnc hosp5_pnc ///
> clinic10_pnc hc10_pnc hosp10_pnc ///
> clinic15_pnc hc15_pnc hosp15_pnc ///
> i.religion i.mhealthcheckb4 i.v024 i.csect i.seasons i.tvradio i.moneyfortreat i.age
i.education i.employment i.wealt
> hrural ///
> i.birthnum ib3.nsize i.nsex [pweight=weight] if residence == 1 & del == 1,
family(binomial) link(logit) corr(exchange
> able)

Iteration 1: tolerance = .05492604
Iteration 2: tolerance = .00628765
Iteration 3: tolerance = .00071345
Iteration 4: tolerance = .00009752
Iteration 5: tolerance = .00001345
Iteration 6: tolerance = 1.808e-06
Iteration 7: tolerance = 2.442e-07

GEE population-averaged model                   Number of obs     =     10,083
Group variable:                   DHSCLUST      Number of groups  =        677
Link:                                logit      Obs per group:
Family:                           binomial                    min =          3
Correlation:                  exchangeable                    avg =       14.8
                                                              max =         24
                                                Wald chi2(35)     =     121.69
Scale parameter:                         1      Prob > chi2       =     0.0000

                                   (Std. Err. adjusted for clustering on DHSCLUST)
--------------------------------------------------------------------------------------
                   |               Robust
       binarympnc1 |      Coef.   Std. Err.      z    P>|z|     [95% Conf. Interval]
-------------------+------------------------------------------------------------------
       clinic5_pnc |   .7213267   .4648088     1.55   0.121    -.1896818    1.632335
           hc5_pnc |  -.1551871   .2079307    -0.75   0.455    -.5627237    .2523495
         hosp5_pnc |  -.9690941   .3623933    -2.67   0.007    -1.679372   -.2588164
      clinic10_pnc |   .1935191   .2879419     0.67   0.502    -.3708367    .7578749
          hc10_pnc |   .0083564   .2433989     0.03   0.973    -.4686966    .4854095
        hosp10_pnc |  -.1127109   .2294112    -0.49   0.623    -.5623486    .3369267
      clinic15_pnc |  -.4595652   .2368834    -1.94   0.052     -.923848    .0047176
          hc15_pnc |  -.3028738   .2393296    -1.27   0.206    -.7719511    .1662036
        hosp15_pnc |  -.3926164   .2204031    -1.78   0.075    -.8245986    .0393658
                   |
          religion |
   other christian |   .1183318   .2110712     0.56   0.575    -.2953601    .5320238
```

```
muslim/no religion  |  -.8845428   .3741159    -2.36   0.018    -1.617796   -.1512892
                    |
   1.mhealthcheckb4 |   1.045036   .1865284     5.60   0.000     .6794467    1.410624
                    |
               v024 |
     central region |   -1.30015   .3058855    -4.25   0.000    -1.899675   -.7006254
    southern region |  -.1568247     .28898    -0.54   0.587    -.7232151    .4095656
                    |
            1.csect |   .1357371   .3444462     0.39   0.694    -.5393651    .8108393
                    |
            seasons |
  winter-dry season |  -.4689713   .1816033    -2.58   0.010    -.8249073   -.1130353
     hot-dry season |  -.3066991   .2243257    -1.37   0.172    -.7463693    .1329712
                    |
          1.tvradio |   .2937194   .1834508     1.60   0.109    -.0658376    .6532763
     1.moneyfortreat |   .2809621   .1875293     1.50   0.134    -.0865885    .6485128
                    |
                age |
              25-34 |  -.0799232   .2136347    -0.37   0.708    -.4986395     .338793
              35-49 |  -.1107844   .3143305    -0.35   0.725    -.7268608    .5052921
                    |
          education |
            primary |  -.0832832    .192382    -0.43   0.665     -.460345    .2937786
          secondary |   .0059672   .5433205     0.01   0.991    -1.058921    1.070856
                    |
       1.employment |  -.1410728    .168207    -0.84   0.402    -.4707525    .1886069
                    |
        wealthrural |
                  2 |   .2439843   .2394932     1.02   0.308    -.2254137    .7133824
                  3 |   .0747846   .2509899     0.30   0.766    -.4171466    .5667159
                  4 |  -.2102195   .2827288    -0.74   0.457    -.7643577    .3439187
                  5 |  -.0479474   .3093492    -0.15   0.877    -.6542606    .5583658
                    |
           birthnum |
                2-3 |  -.0039298   .2223776    -0.02   0.986     -.439782    .4319223
                 4+ |  -.1056672   .2890567    -0.37   0.715    -.6722079    .4608735
                    |
              nsize |
                  1 |   -.236357   .3290085    -0.72   0.473    -.8812019    .4084878
                  2 |  -.2745631   .2016245    -1.36   0.173    -.6697398    .1206137
                  4 |  -.2820862   .2832901    -1.00   0.319    -.8373245    .2731521
                  5 |  -.0988556   .3926725    -0.25   0.801    -.8684795    .6707683
                    |
               nsex |
             female |   .0927126   .1543464     0.60   0.548    -.2098009    .3952261
              _cons |  -3.254268   .5589815    -5.82   0.000    -4.349852   -2.158685
------------------------------------------------------------------------------------

. margins, dydx(*)

Average marginal effects                        Number of obs     =     10,083
Model VCE    : Robust

Expression   : Pr(binarympnc1 != 0), predict()
dy/dx w.r.t. : clinic5_pnc hc5_pnc hosp5_pnc clinic10_pnc hc10_pnc hosp10_pnc clinic15_pnc
```

hc15_pnc hosp15_pnc
2.religion 3.religion 1.mhealthcheckb4 2.v024 3.v024 1.csect 2.seasons
3.seasons 1.tvradio
1.moneyfortreat 2.age 3.age 2.education 3.education 1.employment
2.wealthrural 3.wealthrural
4.wealthrural 5.wealthrural 2.birthnum 3.birthnum 1.nsize 2.nsize 4.nsize
5.nsize 1.nsex

		Delta-method				
	dy/dx	Std. Err.	z	P>|z|	[95% Conf.	Interval]
clinic5_pnc	.0143076	.0094904	1.51	0.132	-.0042934	.0329085
hc5_pnc	-.0030781	.0041321	-0.74	0.456	-.011177	.0050207
hosp5_pnc	-.019222	.007577	-2.54	0.011	-.0340726	-.0043715
clinic10_pnc	.0038385	.0057711	0.67	0.506	-.0074728	.0151497
hc10_pnc	.0001658	.0048301	0.03	0.973	-.0093011	.0096326
hosp10_pnc	-.0022356	.0045921	-0.49	0.626	-.011236	.0067647
clinic15_pnc	-.0091155	.0049898	-1.83	0.068	-.0188954	.0006644
hc15_pnc	-.0060075	.0047532	-1.26	0.206	-.0153236	.0033086
hosp15_pnc	-.0077876	.0044498	-1.75	0.080	-.016509	.0009338
religion						
other christian	.0025614	.0043975	0.58	0.560	-.0060576	.0111803
muslim/no religion	-.0123316	.0052788	-2.34	0.019	-.0226778	-.0019854
1.mhealthcheckb4	.0200537	.0039375	5.09	0.000	.0123364	.027771
v024						
central region	-.023727	.0076962	-3.08	0.002	-.0388113	-.0086427
southern region	-.0045818	.0087207	-0.53	0.599	-.021674	.0125104
1.csect	.0028409	.0076263	0.37	0.710	-.0121063	.0177882
seasons						
winter-dry season	-.0090584	.0034906	-2.60	0.009	-.0158999	-.0022168
hot-dry season	-.0063557	.0044843	-1.42	0.156	-.0151448	.0024334
1.tvradio	.0059821	.0039272	1.52	0.128	-.0017151	.0136792
1.moneyfortreat	.0055003	.0037023	1.49	0.137	-.0017561	.0127566
age						
25-34	-.0016054	.0043093	-0.37	0.709	-.0100514	.0068407
35-49	-.0021944	.0061354	-0.36	0.721	-.0142196	.0098307
education						
primary	-.0016194	.0036944	-0.44	0.661	-.0088602	.0056214
secondary	.0001208	.0110277	0.01	0.991	-.0214931	.0217348
1.employment	-.0028557	.0034523	-0.83	0.408	-.0096221	.0039106
wealthrural						
2	.0053119	.0051224	1.04	0.300	-.0047278	.0153517
3	.0015048	.0050297	0.30	0.765	-.0083533	.0113629
4	-.0037151	.0050463	-0.74	0.462	-.0136056	.0061755

```
              5  |  -.0009119   .0058832    -0.16   0.877    -.0124427    .0106189
                 |
        birthnum |
            2-3  |  -.0000809   .0045811    -0.02   0.986    -.0090596    .0088979
             4+  |  -.0020766   .0057545    -0.36   0.718    -.0133552     .009202
                 |
           nsize |
              1  |  -.0047123   .0060533    -0.78   0.436    -.0165767     .007152
              2  |  -.0053823   .0038152    -1.41   0.158    -.0128599    .0020952
              4  |  -.0055115    .005039    -1.09   0.274    -.0153877    .0043647
              5  |  -.0020957   .0080219    -0.26   0.794    -.0178182    .0136269
                 |
            nsex |
         female  |     .00184   .0030505     0.60   0.546    -.0041389     .007819
------------------------------------------------------------------------------------
Note: dy/dx for factor levels is the discrete change from the base level.

.
. xtgee binarypnc1 clinic5_pnc hc5_pnc hosp5_pnc ///
> clinic10_pnc hc10_pnc hosp10_pnc ///
> clinic15_pnc hc15_pnc hosp15_pnc ///
> i.religion i.newborncheckb4 i.v024 i.csect i.seasons i.tvradio i.moneyfortreat i.age
i.education i.employment i.wealt
> hrural ///
> i.birthnum ib3.nsize i.nsex [pweight=weight] if residence == 1 & del == 1,
family(binomial) link(logit) corr(exchange
> able)

Iteration 1: tolerance = .07039326
Iteration 2: tolerance = .00476555
Iteration 3: tolerance = .00041952
Iteration 4: tolerance = .00003763
Iteration 5: tolerance = 3.219e-06
Iteration 6: tolerance = 2.738e-07

GEE population-averaged model                   Number of obs     =     10,029
Group variable:                   DHSCLUST      Number of groups  =        677
Link:                                logit      Obs per group:
Family:                           binomial                    min =          3
Correlation:                  exchangeable                    avg =       14.7
                                                              max =         24
                                                Wald chi2(35)     =      74.44
Scale parameter:                         1      Prob > chi2       =     0.0001

                                  (Std. Err. adjusted for clustering on DHSCLUST)
------------------------------------------------------------------------------------
                    |               Robust
         binarypnc1 |      Coef.   Std. Err.      z    P>|z|     [95% Conf. Interval]
--------------------+---------------------------------------------------------------
        clinic5_pnc |   .1592292   .4533041     0.35   0.725    -.7292306    1.047689
            hc5_pnc |  -.0878108   .2072615    -0.42   0.672    -.4940359    .3184142
          hosp5_pnc |  -.3815608   .3348106    -1.14   0.254    -1.037778    .2746558
       clinic10_pnc |   .3140642   .2638555     1.19   0.234    -.2030831    .8312115
           hc10_pnc |   .0107584   .2246796     0.05   0.962    -.4296056    .4511224
         hosp10_pnc |  -.2508252   .2200244    -1.14   0.254    -.6820651    .1804148
```

```
      clinic15_pnc |  -.2159594   .2358621    -0.92   0.360    -.6782407    .2463218
          hc15_pnc |  -.0103317   .2760479    -0.04   0.970    -.5513757    .5307122
        hosp15_pnc |  -.5034406   .2162782    -2.33   0.020    -.9273381   -.0795432
                   |
          religion |
  other christian  |  -.0079197   .2414954    -0.03   0.974    -.4812419    .4654026
muslim/no religion |  -.8244875   .3700309    -2.23   0.026    -1.549735   -.0992403
                   |
  1.newborncheckb4 |   .8187613   .2180956     3.75   0.000     .3913018    1.246221
                   |
              v024 |
   central region  |  -.1662744     .34166    -0.49   0.626    -.8359157    .5033668
  southern region  |   .6942752   .3253216     2.13   0.033     .0566566    1.331894
                   |
           1.csect |  -.8705064   .5477849    -1.59   0.112    -1.944145    .2031322
                   |
           seasons |
winter-dry season  |  -.0378576    .168134    -0.23   0.822    -.3673942     .291679
   hot-dry season  |  -.3442228   .2252901    -1.53   0.127    -.7857833    .0973376
                   |
         1.tvradio |   .5112954   .1654452     3.09   0.002     .1870288     .835562
  1.moneyfortreat  |    .222512   .1906698     1.17   0.243     -.151194     .596218
                   |
               age |
            25-34  |  -.0571585   .2497972    -0.23   0.819    -.5467521    .4324351
            35-49  |   .0138417   .3213793     0.04   0.966    -.6160502    .6437336
                   |
         education |
          primary  |  -.2265254   .2171458    -1.04   0.297    -.6521233    .1990725
        secondary  |  -.4866257   .4533709    -1.07   0.283    -1.375216     .401965
                   |
      1.employment |   .2196585   .1918304     1.15   0.252    -.1563222    .5956392
                   |
       wealthrural |
                2  |   .1244027   .2300126     0.54   0.589    -.3264137     .575219
                3  |  -.0551581   .2331566    -0.24   0.813    -.5121367    .4018205
                4  |   .0858332   .2563297     0.33   0.738    -.4165638    .5882303
                5  |  -.5613781   .2962781    -1.89   0.058    -1.142072    .0193162
                   |
          birthnum |
              2-3  |  -.0420841   .2375211    -0.18   0.859    -.5076168    .4234487
               4+  |  -.2449537   .2972758    -0.82   0.410    -.8276035    .3376962
                   |
             nsize |
                1  |  -.1144312   .3137006    -0.36   0.715     -.729273    .5004107
                2  |   .0998381   .2140163     0.47   0.641    -.3196262    .5193024
                4  |   .2353946   .2447188     0.96   0.336    -.2442454    .7150345
                5  |  -.1651065   .3894495    -0.42   0.672    -.9284135    .5982006
                   |
              nsex |
           female  |  -.0734919   .1552193    -0.47   0.636    -.3777161    .2307323
             _cons |  -4.708283   .6046381    -7.79   0.000    -5.893352   -3.523214
------------------------------------------------------------------------------------

. margins, dydx(*)
```

```
Average marginal effects                        Number of obs     =     10,029
Model VCE    : Robust

Expression   : Pr(binarypnc1 != 0), predict()
dy/dx w.r.t. : clinic5_pnc hc5_pnc hosp5_pnc clinic10_pnc hc10_pnc hosp10_pnc clinic15_pnc
hc15_pnc hosp15_pnc
               2.religion 3.religion 1.newborncheckb4 2.v024 3.v024 1.csect 2.seasons
3.seasons 1.tvradio
               1.moneyfortreat 2.age 3.age 2.education 3.education 1.employment
2.wealthrural 3.wealthrural
               4.wealthrural 5.wealthrural 2.birthnum 3.birthnum 1.nsize 2.nsize 4.nsize
5.nsize 1.nsex

------------------------------------------------------------------------------------
                    |            Delta-method
                    |      dy/dx   Std. Err.      z    P>|z|     [95% Conf. Interval]
--------------------+---------------------------------------------------------------
        clinic5_pnc |   .0027992    .007973     0.35   0.726    -.0128276    .0184259
            hc5_pnc |  -.0015437   .0036471    -0.42   0.672    -.0086918    .0056045
          hosp5_pnc |  -.0067076   .0059724    -1.12   0.261    -.0184133    .0049981
       clinic10_pnc |   .0055211    .004743     1.16   0.244    -.0037751    .0148173
           hc10_pnc |   .0001891   .0039509     0.05   0.962    -.0075546    .0079328
         hosp10_pnc |  -.0044094   .0039768    -1.11   0.268    -.0122038    .0033851
       clinic15_pnc |  -.0037964   .0042074    -0.90   0.367    -.0120429      .00445
           hc15_pnc |  -.0001816   .0048517    -0.04   0.970    -.0096907    .0093275
         hosp15_pnc |  -.0088502   .0039717    -2.23   0.026    -.0166345   -.0010659
                    |
           religion |
    other christian |  -.0001562    .004776    -0.03   0.974    -.0095169    .0092045
muslim/no religion  |  -.0113348   .0053333    -2.13   0.034    -.0217878   -.0008818
                    |
    1.newborncheckb4 |   .0122978   .0030758     4.00   0.000     .0062693    .0183263
                    |
               v024 |
     central region |  -.0020388   .0043343    -0.47   0.638    -.0105338    .0064563
    southern region |   .0130318   .0054997     2.37   0.018     .0022526    .0238109
                    |
            1.csect |  -.0107475   .0046005    -2.34   0.019    -.0197643   -.0017307
                    |
            seasons |
  winter-dry season |  -.0006989   .0030942    -0.23   0.821    -.0067634    .0053655
     hot-dry season |  -.0055251   .0033612    -1.64   0.100    -.0121129    .0010627
                    |
          1.tvradio |   .0094735   .0034967     2.71   0.007     .0026201    .0163269
    1.moneyfortreat |     .00383   .0032849     1.17   0.244    -.0026082    .0102683
                    |
                age |
              25-34 |  -.0009972   .0043838    -0.23   0.820    -.0095893    .0075949
              35-49 |   .0002496      .0058     0.04   0.966    -.0111181    .0116174
                    |
          education |
            primary |  -.0037905    .003419    -1.11   0.268    -.0104917    .0029107
          secondary |   -.007248   .0054712    -1.32   0.185    -.0179713    .0034753
                    |
```

1.employment	.0037014	.0031482	1.18	0.240	-.002469	.0098717
wealthrural						
2	.0024107	.0044498	0.54	0.588	-.0063107	.0111322
3	-.0009822	.0041561	-0.24	0.813	-.009128	.0071636
4	.0016331	.0048891	0.33	0.738	-.0079493	.0112155
5	-.0079551	.004196	-1.90	0.058	-.0161791	.000269
birthnum						
2-3	-.0008083	.0045917	-0.18	0.860	-.0098078	.0081913
4+	-.004287	.0053209	-0.81	0.420	-.0147158	.0061418
nsize						
1	-.0018354	.0048715	-0.38	0.706	-.0113833	.0077125
2	.0017711	.0038497	0.46	0.645	-.0057741	.0093163
4	.0044565	.004936	0.90	0.367	-.0052179	.0141308
5	-.0025868	.0057046	-0.45	0.650	-.0137677	.008594
nsex						
female	-.001291	.0027339	-0.47	0.637	-.0066493	.0040673

Note: dy/dx for factor levels is the discrete change from the base level.

.
end of do-file
