# Supplementary material for: Availability of health facilities and utilization of maternal and newborn postnatal care in rural Malawi
Source: BMC Pregnancy Childbirth. 2019 Dec 17;19:503. doi: 10.1186/s12884-019-2534-x (PMC6918704; doi:10.1186/s12884-019-2534-x)
Supplement: Supplementary file 3 — Additional file 3. Stata log output for sensitivity analyses with the full design effect. [file 12884_2019_2534_MOESM3_ESM.pdf]

List of variables:

/***Set up***/
DHSCLUST = clustering variable
Weight = individual sampling weight
del = place of delivery (0 for home delivery and 1 for facility delivery)
del2 = place of delivery (1 for home delivery and 0 for facility delivery)
v021 = primary sampling unit
v022 = stratification used
residence = urban/rural (1 for rural and 0 for urban)

/***Outcomes***/

binarympnc1 = maternal postnatal care within 1 day
binarympnc7 = maternal postnatal care within 7 days
binarypnc1 = newborn postnatal care within 1 day
binarypnc7 = newborn postnatal care within 7 days

/***Facility Indicators***/

clinic5_pnc = clinic-level facilities providing postnatal care within 5 km of household cluster
hc5_pnc = health centers providing postnatal care within 5 km of household cluster
hosp5_pnc = hospitals providing postnatal care within 5 km of household cluster
clinic10_pnc = clinic-level facilities providing postnatal care between 5 km and 10 km of household cluster
hc10_pnc = health centers providing postnatal care between 5 km and 10 km of household cluster
hosp10_pnc = hospitals providing postnatal care between 5 km and 10 km of household cluster
clinic15_pnc = clinic-level facilities providing postnatal care between 10 km and 15 km of household cluster
hc15_pnc = health centers providing postnatal care between 10 km and 15 km of household cluster
hosp15_pnc = hospitals providing postnatal care between 10 km and 15 km of household cluster

/***Covariates***/

religion = women's religion
v024 = region of the country where women live
seasons = seasons in which women gave birth
tvradio = ownership of TV or radio
moneyfortreat = cost of treatment being a perceived problem
age = women's age
education = women's education
employment = women's employment
wealthrural = rural-specific household wealth quintile
birthnum = women's number of births
nsize = newborn size
nsex = newborn sex
mhealthcheckb4 = maternal health checked before facility discharge (for facility births)
newborncheckb4 = newborn health checked before facility discharge (for facility births)
csect = receipt of cesarean section

```
. /*******************************************
> sensitivity analysis:
> running
>
> svy:logit
>
> for comparison with xtgee
> *******************************************/
.
. /******
> Home
> Delivery
> Only
> *******/
.
. ****Survey weights
. gen weight = v005/1000000
(1 missing value generated)

. svyset [pweight=weight], psu(v021) strata(v022)

      pweight: weight
          VCE: linearized
  Single unit: missing
     Strata 1: v022
         SU 1: v021
        FPC 1: <zero>

.
. ***Analysis commands
. svy, subpop(del2 if residence == 1):logit binarympnc1 clinic5_pnc hc5_pnc hosp5_pnc ///
> clinic10_pnc hc10_pnc hosp10_pnc ///
> clinic15_pnc hc15_pnc hosp15_pnc ///
> i.religion i.v024 i.seasons i.tvradio i.moneyfortreat i.age i.education i.employment
i.wealthrural ///
> i.birthnum i.nsize i.nsex
(running logit on estimation sample)

Survey: Logistic regression

Number of strata   =        28                 Number of obs     =     10,931
Number of PSUs     =       677                 Population size   = 11,371.089
                                               Subpop. no. obs   =        665
                                               Subpop. size      = 771.762269
                                               Design df         =        649
                                               F(  33,    617)   =       2.47
                                               Prob > F          =     0.0000

--------------------------------------------------------------------------------------
                     |             Linearized
         binarympnc1 |      Coef.   Std. Err.      t    P>|t|     [95% Conf. Interval]
---------------------+----------------------------------------------------------------
         clinic5_pnc |   .4689543   .7146189     0.66   0.512      -.93429    1.872199
             hc5_pnc |   .8037216      .3017     2.66   0.008     .2112956    1.396148
           hosp5_pnc |   .2826816   .4181037     0.68   0.499    -.5383176    1.103681
```

```
      clinic10_pnc |  -.3227672   .4059522    -0.80   0.427    -1.119905    .4743711
          hc10_pnc |    .392799    .346284     1.13   0.257    -.2871733    1.072771
        hosp10_pnc |    .633105   .3239283     1.95   0.051     -.002969    1.269179
      clinic15_pnc |  -.6292756   .3794965    -1.66   0.098    -1.374465    .1159135
          hc15_pnc |  -.1185838   .4212724    -0.28   0.778    -.9458053    .7086377
        hosp15_pnc |   .2730744   .3114736     0.88   0.381    -.3385432     .884692
                   |
          religion |
  other christian  |   .4941307   .5273263     0.94   0.349    -.5413409    1.529602
muslim/no religion  |   .8281798   .6082934     1.36   0.174    -.3662809     2.02264
                   |
              v024 |
    central region  |  -.5605679     .49082    -1.14   0.254    -1.524355     .403219
  southern region  |  -.6426761    .472115    -1.36   0.174    -1.569733    .2843813
                   |
           seasons |
 winter-dry season  |  -1.333253   .4478779    -2.98   0.003    -2.212717    -.453788
    hot-dry season  |  -.3137807   .4660865    -0.67   0.501       -1.229    .6014388
                   |
         1.tvradio |   .7963471    .296584     2.69   0.007      .213967    1.378727
   1.moneyfortreat |  -.1847489   .3113118    -0.59   0.553    -.7960489    .4265511
                   |
               age |
            25-34  |   .2439738   .4325908     0.56   0.573    -.6054727     1.09342
            35-49  |   .1996337    .660266     0.30   0.762    -1.096882    1.496149
                   |
         education |
          primary  |   .7224109   .4830036     1.50   0.135    -.2260276    1.670849
        secondary  |   .0301387   1.064968     0.03   0.977     -2.06106    2.121337
                   |
      1.employment |   .8265339   .3362284     2.46   0.014     .1663071    1.486761
                   |
       wealthrural |
                2  |  -1.527109   .4902645    -3.11   0.002    -2.489805   -.5644125
                3  |  -.1969838   .3300693    -0.60   0.551    -.8451165    .4511488
                4  |  -.8908902   .4495559    -1.98   0.048     -1.77365   -.0081306
                5  |  -1.690299    .665144    -2.54   0.011    -2.996393   -.3842046
                   |
          birthnum |
              2-3  |  -.8887321   .6028721    -1.47   0.141    -2.072547    .2950831
               4+  |  -.5611786   .6960806    -0.81   0.420    -1.928021    .8056632
                   |
             nsize |
                2  |   .6988296   .5989976     1.17   0.244    -.4773776    1.875037
                3  |   1.121127   .5238846     2.14   0.033     .0924132     2.14984
                4  |  -.1057045   .6301303    -0.17   0.867    -1.343045    1.131636
                5  |   .0920459   .9426445     0.10   0.922    -1.758955    1.943047
                   |
              nsex |
           female  |   .3744744   .3664653     1.02   0.307    -.3451264    1.094075
             _cons |   -3.03974    .847856    -3.59   0.000    -4.704612   -1.374868
------------------------------------------------------------------------------------
Note: 28 strata omitted because they contain no subpopulation members.

. margins, dydx(*)
```

```
Average marginal effects                        Number of obs     =     10,782
Model VCE    : Linearized

Expression   : Pr(binarympnc1), predict()
dy/dx w.r.t. : clinic5_pnc hc5_pnc hosp5_pnc clinic10_pnc hc10_pnc hosp10_pnc clinic15_pnc
hc15_pnc hosp15_pnc
               2.religion 3.religion 2.v024 3.v024 2.seasons 3.seasons 1.tvradio
1.moneyfortreat 2.age 3.age
               2.education 3.education 1.employment 2.wealthrural 3.wealthrural
4.wealthrural 5.wealthrural
               2.birthnum 3.birthnum 2.nsize 3.nsize 4.nsize 5.nsize 1.nsex
```

| | dy/dx | Delta-method Std. Err. | t | P>\|t\| | [95% Conf. | Interval] |
|---|---|---|---|---|---|---|
| clinic5_pnc | .0479641 | .0733025 | 0.65 | 0.513 | -.0959746 | .1919029 |
| hc5_pnc | .0822038 | .032445 | 2.53 | 0.012 | .0184939 | .1459136 |
| hosp5_pnc | .0289124 | .0424643 | 0.68 | 0.496 | -.0544716 | .1122964 |
| clinic10_pnc | -.0330123 | .0418082 | -0.79 | 0.430 | -.115108 | .0490835 |
| hc10_pnc | .0401751 | .0351596 | 1.14 | 0.254 | -.0288653 | .1092154 |
| hosp10_pnc | .0647533 | .033299 | 1.94 | 0.052 | -.0006336 | .1301401 |
| clinic15_pnc | -.0643616 | .0396477 | -1.62 | 0.105 | -.1422149 | .0134916 |
| hc15_pnc | -.0121286 | .0429584 | -0.28 | 0.778 | -.0964828 | .0722255 |
| hosp15_pnc | .0279298 | .0318152 | 0.88 | 0.380 | -.0345434 | .0904029 |
| religion | | | | | | |
| other christian | .0447762 | .0437058 | 1.02 | 0.306 | -.0410456 | .1305979 |
| muslim/no religion | .0825148 | .0588964 | 1.40 | 0.162 | -.0331357 | .1981654 |
| v024 | | | | | | |
| central region | -.0651657 | .0610947 | -1.07 | 0.287 | -.1851328 | .0548013 |
| southern region | -.0732573 | .0588858 | -1.24 | 0.214 | -.1888869 | .0423724 |
| seasons | | | | | | |
| winter-dry season | -.1213696 | .0394508 | -3.08 | 0.002 | -.1988362 | -.0439029 |
| hot-dry season | -.0375258 | .0540258 | -0.69 | 0.488 | -.1436122 | .0685607 |
| 1.tvradio | .0853553 | .0332589 | 2.57 | 0.010 | .0200472 | .1506634 |
| 1.moneyfortreat | -.0190336 | .0323757 | -0.59 | 0.557 | -.0826073 | .0445401 |
| age | | | | | | |
| 25-34 | .024656 | .0429316 | 0.57 | 0.566 | -.0596457 | .1089577 |
| 35-49 | .0199349 | .0666485 | 0.30 | 0.765 | -.1109378 | .1508076 |
| education | | | | | | |
| primary | .0821587 | .0605479 | 1.36 | 0.175 | -.0367348 | .2010522 |
| secondary | .0028505 | .101516 | 0.03 | 0.978 | -.1964889 | .2021899 |
| 1.employment | .0776218 | .0289017 | 2.69 | 0.007 | .0208698 | .1343739 |
| wealthrural | | | | | | |
| 2 | -.1583194 | .0496133 | -3.19 | 0.001 | -.2557414 | -.0608974 |
| 3 | -.0277231 | .0464445 | -0.60 | 0.551 | -.1189226 | .0634765 |

```
              4  |  -.1078314   .0536285    -2.01   0.045    -.2131377   -.0025251
              5  |   -.168184   .0557598    -3.02   0.003    -.2776755   -.0586925
                 |
        birthnum |
            2-3  |  -.0961441   .0733938    -1.31   0.191    -.2402621    .0479739
             4+  |  -.0657786   .0854176    -0.77   0.442    -.2335068    .1019497
                 |
           nsize |
              2  |   .0568726   .0460358     1.24   0.217    -.0335245    .1472696
              3  |   .1042727   .0392052     2.66   0.008     .0272885     .181257
              4  |  -.0065223   .0391764    -0.17   0.868    -.0834502    .0704056
              5  |     .00609   .0635255     0.10   0.924    -.1186502    .1308302
                 |
            nsex |
         female  |   .0382554   .0374969     1.02   0.308    -.0353745    .1118854
------------------------------------------------------------------------------------
Note: dy/dx for factor levels is the discrete change from the base level.

.
. svy, subpop(del2 if residence == 1):logit binarympnc7 clinic5_pnc hc5_pnc hosp5_pnc ///
> clinic10_pnc hc10_pnc hosp10_pnc ///
> clinic15_pnc hc15_pnc hosp15_pnc ///
> i.religion i.v024 i.seasons i.tvradio i.moneyfortreat i.age i.education i.employment
i.wealthrural ///
> i.birthnum i.nsize i.nsex
(running logit on estimation sample)

Survey: Logistic regression

Number of strata   =        28                 Number of obs     =     10,931
Number of PSUs     =       677                 Population size   = 11,371.089
                                               Subpop. no. obs   =        665
                                               Subpop. size      = 771.762269
                                               Design df         =        649
                                               F(  33,     617)  =       1.83
                                               Prob > F          =     0.0036

------------------------------------------------------------------------------------
                 |             Linearized
     binarympnc7 |      Coef.   Std. Err.      t    P>|t|     [95% Conf. Interval]
-----------------+------------------------------------------------------------------
     clinic5_pnc |   1.010565   .5679035     1.78   0.076    -.1045847    2.125715
         hc5_pnc |   .5799468   .2602093     2.23   0.026     .0689931    1.090901
       hosp5_pnc |  -.0438926   .3557276    -0.12   0.902    -.7424086    .6546233
    clinic10_pnc |  -.0400707   .3032325    -0.13   0.895    -.6355058    .5553645
        hc10_pnc |   .2300665   .2702491     0.85   0.395    -.3006017    .7607347
      hosp10_pnc |   .5833298   .2672668     2.18   0.029     .0585178    1.108142
    clinic15_pnc |  -.6066854   .3237301    -1.87   0.061     -1.24237    .0289994
        hc15_pnc |   -.291712   .3539768    -0.82   0.410      -.98679    .4033661
      hosp15_pnc |   .2083065    .276439     0.75   0.451    -.3345163    .7511293
                 |
        religion |
 other christian  |   .3857305   .4442921     0.87   0.386     -.486693    1.258154
muslim/no religion  |   .5223289   .5140244     1.02   0.310    -.4870228    1.531681
                 |
```

```
                v024 |
      central region |  -.1612813   .4006657    -0.40   0.687    -.9480389    .6254764
     southern region |    -.33149   .3953327    -0.84   0.402    -1.107775    .4447955
                     |
             seasons |
   winter-dry season |  -.8350818   .3662734    -2.28   0.023    -1.554306   -.1158579
      hot-dry season |  -.2030324   .4256096    -0.48   0.633     -1.03877    .6327057
                     |
           1.tvradio |   .4994311   .2880322     1.73   0.083    -.0661565    1.065019
    1.moneyfortreat |  -.0078145   .2906806    -0.03   0.979    -.5786025    .5629735
                     |
                 age |
               25-34 |  -.2695913   .4467409    -0.60   0.546    -1.146823    .6076408
               35-49 |  -.2140526   .6557444    -0.33   0.744    -1.501689    1.073584
                     |
           education |
             primary |   .8956033   .4773486     1.88   0.061    -.0417308    1.832937
           secondary |  -.2039027   1.077233    -0.19   0.850    -2.319186     1.91138
                     |
        1.employment |   .6599705   .2832651     2.33   0.020     .1037438    1.216197
                     |
         wealthrural |
                   2 |  -1.108439   .4947965    -2.24   0.025    -2.080034   -.1368433
                   3 |  -.2081132   .3123494    -0.67   0.505    -.8214507    .4052242
                   4 |  -.4441996   .4768812    -0.93   0.352    -1.380616    .4922166
                   5 |  -1.374367   .6318316    -2.18   0.030    -2.615048   -.1336859
                     |
            birthnum |
                 2-3 |  -.8608219   .4877562    -1.76   0.078    -1.818593    .0969489
                  4+ |  -.5000216   .6184606    -0.81   0.419    -1.714447    .7144037
                     |
               nsize |
                   2 |  -.1259452   .6424106    -0.20   0.845    -1.387399    1.135509
                   3 |  -.0054432   .5561964    -0.01   0.992    -1.097605    1.086718
                   4 |  -.5844485    .601901    -0.97   0.332    -1.766357    .5974599
                   5 |  -.8158454   .8690238    -0.94   0.348    -2.522283    .8905923
                     |
                nsex |
              female |   .4490196   .3488796     1.29   0.199    -.2360494    1.134089
               _cons |  -1.615258   .7297289    -2.21   0.027    -3.048173   -.1823435
--------------------------------------------------------------------------------------
Note: 28 strata omitted because they contain no subpopulation members.

. margins, dydx(*)

Average marginal effects                        Number of obs     =     10,782
Model VCE    : Linearized

Expression   : Pr(binarympnc7), predict()
dy/dx w.r.t. : clinic5_pnc hc5_pnc hosp5_pnc clinic10_pnc hc10_pnc hosp10_pnc clinic15_pnc
hc15_pnc hosp15_pnc
               2.religion 3.religion 2.v024 3.v024 2.seasons 3.seasons 1.tvradio
1.moneyfortreat 2.age 3.age
               2.education 3.education 1.employment 2.wealthrural 3.wealthrural
4.wealthrural 5.wealthrural
```

2.birthnum 3.birthnum 2.nsize 3.nsize 4.nsize 5.nsize 1.nsex

| | dy/dx | Delta-method Std. Err. | t | P>\|t\| | [95% Conf. | Interval] |
|---|---|---|---|---|---|---|
| clinic5_pnc | .1282709 | .0724998 | 1.77 | 0.077 | -.0140915 | .2706333 |
| hc5_pnc | .0736126 | .033853 | 2.17 | 0.030 | .007138 | .1400871 |
| hosp5_pnc | -.0055713 | .0452215 | -0.12 | 0.902 | -.0943694 | .0832268 |
| clinic10_pnc | -.0050862 | .0385311 | -0.13 | 0.895 | -.0807468 | .0705744 |
| hc10_pnc | .0292023 | .0342889 | 0.85 | 0.395 | -.0381282 | .0965328 |
| hosp10_pnc | .074042 | .0339088 | 2.18 | 0.029 | .0074578 | .1406261 |
| clinic15_pnc | -.0770065 | .0418068 | -1.84 | 0.066 | -.1590995 | .0050865 |
| hc15_pnc | -.037027 | .0446509 | -0.83 | 0.407 | -.1247047 | .0506508 |
| hosp15_pnc | .0264403 | .0351017 | 0.75 | 0.452 | -.0424863 | .0953669 |
| religion | | | | | | |
| other christian | .0451919 | .0483457 | 0.93 | 0.350 | -.0497409 | .1401247 |
| muslim/no religion | .0634917 | .0607699 | 1.04 | 0.297 | -.0558377 | .1828211 |
| v024 | | | | | | |
| central region | -.0219297 | .0555521 | -0.39 | 0.693 | -.1310132 | .0871538 |
| southern region | -.0432455 | .0538751 | -0.80 | 0.422 | -.1490361 | .0625451 |
| seasons | | | | | | |
| winter-dry season | -.099645 | .0428263 | -2.33 | 0.020 | -.1837397 | -.0155502 |
| hot-dry season | -.0284497 | .0584503 | -0.49 | 0.627 | -.1432243 | .0863249 |
| 1.tvradio | .0652734 | .0386027 | 1.69 | 0.091 | -.0105279 | .1410746 |
| 1.moneyfortreat | -.0009922 | .0369204 | -0.03 | 0.979 | -.07349 | .0715056 |
| age | | | | | | |
| 25-34 | -.0346672 | .0586702 | -0.59 | 0.555 | -.1498736 | .0805391 |
| 35-49 | -.0279146 | .085002 | -0.33 | 0.743 | -.1948268 | .1389976 |
| education | | | | | | |
| primary | .1298332 | .0751711 | 1.73 | 0.085 | -.0177747 | .2774412 |
| secondary | -.0221665 | .11065 | -0.20 | 0.841 | -.2394418 | .1951087 |
| 1.employment | .0784521 | .0311495 | 2.52 | 0.012 | .0172862 | .1396181 |
| wealthrural | | | | | | |
| 2 | -.1417127 | .0603833 | -2.35 | 0.019 | -.2602829 | -.0231424 |
| 3 | -.0327464 | .0491345 | -0.67 | 0.505 | -.1292281 | .0637353 |
| 4 | -.06647 | .0696855 | -0.95 | 0.341 | -.2033063 | .0703663 |
| 5 | -.1642446 | .0647513 | -2.54 | 0.011 | -.2913919 | -.0370972 |
| birthnum | | | | | | |
| 2-3 | -.1143512 | .0721603 | -1.58 | 0.114 | -.2560471 | .0273446 |
| 4+ | -.0724184 | .0921112 | -0.79 | 0.432 | -.2532903 | .1084535 |
| nsize | | | | | | |
| 2 | -.0166103 | .0859209 | -0.19 | 0.847 | -.1853269 | .1521063 |
| 3 | -.0007393 | .0756204 | -0.01 | 0.992 | -.1492295 | .1477509 |
| 4 | -.0684348 | .0762685 | -0.90 | 0.370 | -.2181976 | .081328 |

```
                   5 |  -.0896721   .0904308    -0.99   0.322    -.2672444    .0879003
                     |
                nsex |
              female |   .0569735   .0439342     1.30   0.195    -.0292968    .1432438
--------------------------------------------------------------------------------------
Note: dy/dx for factor levels is the discrete change from the base level.

.
. svy, subpop(del2 if residence == 1):logit binarypnc1 clinic5_pnc hc5_pnc hosp5_pnc ///
> clinic10_pnc hc10_pnc hosp10_pnc ///
> clinic15_pnc hc15_pnc hosp15_pnc ///
> i.religion i.v024 i.seasons i.tvradio i.moneyfortreat i.age i.education i.employment
i.wealthrural ///
> i.birthnum i.nsize i.nsex
(running logit on estimation sample)

Survey: Logistic regression

Number of strata   =        28                Number of obs     =     10,930
Number of PSUs     =       677                Population size   = 11,369.917
                                              Subpop. no. obs   =        664
                                              Subpop. size      = 770.591103
                                              Design df         =        649
                                              F(  33,    617)   =       1.58
                                              Prob > F          =     0.0221

--------------------------------------------------------------------------------------
                     |             Linearized
          binarypnc1 |      Coef.   Std. Err.      t    P>|t|     [95% Conf. Interval]
---------------------+----------------------------------------------------------------
         clinic5_pnc |  -.3307765   .6829086    -0.48   0.628    -1.671754    1.010201
             hc5_pnc |   .6283333   .3056533     2.06   0.040     .0281446    1.228522
           hosp5_pnc |   1.274421   .5728891     2.22   0.026     .1494806    2.399361
        clinic10_pnc |   .2810775   .2988207     0.94   0.347    -.3056947    .8678496
            hc10_pnc |   .2579889   .3348703     0.77   0.441    -.3995712     .915549
          hosp10_pnc |    .278517   .2838075     0.98   0.327    -.2787748    .8358088
        clinic15_pnc |  -.0780185    .328598    -0.24   0.812    -.7232621     .567225
            hc15_pnc |    .464243   .4128062     1.12   0.261    -.3463539     1.27484
          hosp15_pnc |  -.0023731   .2796953    -0.01   0.993    -.5515901    .5468438
                     |
            religion |
     other christian |   .3394777   .4191723     0.81   0.418    -.4836199    1.162575
  muslim/no religion |    .671941   .5342402     1.26   0.209    -.3771068    1.720989
                     |
                v024 |
      central region |  -.8275759   .4482294    -1.85   0.065    -1.707731    .0525789
     southern region |  -1.458329   .5393457    -2.70   0.007    -2.517402   -.3992554
                     |
             seasons |
   winter-dry season |  -.5308535   .3678137    -1.44   0.149    -1.253102    .1913951
      hot-dry season |  -.0220792   .3594674    -0.06   0.951    -.7279387    .6837803
                     |
           1.tvradio |   .3235816   .2803122     1.15   0.249    -.2268467    .8740099
     1.moneyfortreat |   .1965356   .3001651     0.65   0.513    -.3928763    .7859475
                     |
```

```
                 age |
               25-34 |    .974943   .3964857     2.46   0.014     .1963934    1.753493
               35-49 |   .6620833    .595945     1.11   0.267    -.5081298    1.832297
                     |
           education |
             primary |  -.1667018   .3474668    -0.48   0.632    -.8489967    .5155931
           secondary |  -1.352266   1.160585    -1.17   0.244    -3.631222    .9266899
                     |
        1.employment |   .5760594   .2965268     1.94   0.052    -.0062084    1.158327
                     |
         wealthrural |
                   2 |   .8107578   .4317403     1.88   0.061    -.0370187    1.658534
                   3 |   1.003543   .4585756     2.19   0.029      .103072    1.904014
                   4 |      .6923   .4927385     1.41   0.160    -.2752541    1.659854
                   5 |    .799241    .492614     1.62   0.105    -.1680686    1.766551
                     |
            birthnum |
                 2-3 |  -.8993462   .5121601    -1.76   0.080    -1.905037    .1063447
                  4+ |  -1.343681   .6579481    -2.04   0.042    -2.635645   -.0517166
                     |
               nsize |
                   2 |  -.4238177   .4752349    -0.89   0.373    -1.357001     .509366
                   3 |  -.7011984   .4589023    -1.53   0.127    -1.602311    .1999141
                   4 |  -1.156791   .5776012    -2.00   0.046    -2.290984   -.0225985
                   5 |  -1.839907   .8451465    -2.18   0.030    -3.499459   -.1803555
                     |
                nsex |
              female |   .0028952   .3248287     0.01   0.993    -.6349468    .6407372
               _cons |  -2.375061   .8358587    -2.84   0.005    -4.016374   -.7337466
--------------------------------------------------------------------------------------
Note: 28 strata omitted because they contain no subpopulation members.

. margins, dydx(*)

Average marginal effects                        Number of obs     =     10,781
Model VCE    : Linearized

Expression   : Pr(binarypnc1), predict()
dy/dx w.r.t. : clinic5_pnc hc5_pnc hosp5_pnc clinic10_pnc hc10_pnc hosp10_pnc clinic15_pnc
hc15_pnc hosp15_pnc
               2.religion 3.religion 2.v024 3.v024 2.seasons 3.seasons 1.tvradio
1.moneyfortreat 2.age 3.age
               2.education 3.education 1.employment 2.wealthrural 3.wealthrural
4.wealthrural 5.wealthrural
               2.birthnum 3.birthnum 2.nsize 3.nsize 4.nsize 5.nsize 1.nsex

--------------------------------------------------------------------------------------
                     |            Delta-method
                     |      dy/dx   Std. Err.      t    P>|t|     [95% Conf. Interval]
---------------------+----------------------------------------------------------------
         clinic5_pnc |  -.0397815   .0823283    -0.48   0.629    -.2014435    .1218804
             hc5_pnc |   .0755679   .0379101     1.99   0.047     .0011267    .1500091
           hosp5_pnc |   .1532709   .0677848     2.26   0.024      .020167    .2863749
        clinic10_pnc |   .0338044   .0359256     0.94   0.347    -.0367401    .1043489
            hc10_pnc |   .0310276   .0399768     0.78   0.438    -.0474719    .1095271
```

```
         hosp10_pnc |   .0334964   .0339459     0.99   0.324    -.0331605    .1001534
        clinic15_pnc |  -.0093831    .039679    -0.24   0.813    -.0872978    .0685317
            hc15_pnc |   .0558332   .0492919     1.13   0.258    -.0409577    .1526241
          hosp15_pnc |  -.0002854   .0336425    -0.01   0.993    -.0663466    .0657758
                     |
            religion |
     other christian  |   .0373268   .0433931     0.86   0.390    -.0478811    .1225346
  muslim/no religion  |   .0809398   .0651453     1.24   0.215    -.0469811    .2088608
                     |
                v024 |
      central region  |  -.1315806   .0764672    -1.72   0.086    -.2817336    .0185724
     southern region  |  -.2022867   .0810867    -2.49   0.013    -.3615106   -.0430628
                     |
             seasons |
   winter-dry season  |  -.0607054   .0398364    -1.52   0.128    -.1389292    .0175185
      hot-dry season  |   -.002887   .0468676    -0.06   0.951    -.0949174    .0891433
                     |
           1.tvradio |   .0396234   .0349761     1.13   0.258    -.0290566    .1083034
     1.moneyfortreat |   .0234543   .0353501     0.66   0.507    -.0459601    .0928687
                     |
                 age |
               25-34  |   .1137246   .0444886     2.56   0.011     .0263656    .2010835
               35-49  |   .0707946   .0671124     1.05   0.292     -.060989    .2025782
                     |
           education |
             primary  |  -.0200914   .0406646    -0.49   0.621    -.0999415    .0597587
           secondary  |  -.1168236   .0647534    -1.80   0.072    -.2439751    .0103279
                     |
        1.employment |   .0651955    .031985     2.04   0.042     .0023889    .1280021
                     |
         wealthrural |
                   2  |   .0849796   .0452978     1.88   0.061    -.0039682    .1739275
                   3  |   .1115359   .0519835     2.15   0.032     .0094597     .213612
                   4  |   .0699088   .0505561     1.38   0.167    -.0293645    .1691821
                   5  |   .0834728   .0540009     1.55   0.123    -.0225647    .1895103
                     |
            birthnum |
                 2-3  |  -.1321057   .0830073    -1.59   0.112     -.295101    .0308895
                  4+  |  -.1791818   .0962826    -1.86   0.063    -.3682448    .0098812
                     |
               nsize |
                   2  |  -.0635262   .0749911    -0.85   0.397    -.2107806    .0837282
                   3  |  -.0986173   .0719012    -1.37   0.171    -.2398044    .0425698
                   4  |  -.1453921   .0784517    -1.85   0.064    -.2994419    .0086577
                   5  |  -.1934661   .0786953    -2.46   0.014    -.3479942    -.038938
                     |
                nsex |
              female  |   .0003482   .0390675     0.01   0.993    -.0763658    .0770622
--------------------------------------------------------------------------------------
Note: dy/dx for factor levels is the discrete change from the base level.

.
. svy, subpop(del2 if residence == 1):logit binarypnc7 clinic5_pnc hc5_pnc hosp5_pnc ///
> clinic10_pnc hc10_pnc hosp10_pnc ///
> clinic15_pnc hc15_pnc hosp15_pnc ///
```

```
> i.religion i.v024 i.seasons i.tvradio i.moneyfortreat i.age i.education i.employment
i.wealthrural ///
> i.birthnum i.nsize i.nsex
(running logit on estimation sample)

Survey: Logistic regression

Number of strata   =        28                  Number of obs     =     10,930
Number of PSUs     =       677                  Population size   = 11,369.917
                                                Subpop. no. obs   =        664
                                                Subpop. size      = 770.591103
                                                Design df         =        649
                                                F(  33,    617)   =       1.00
                                                Prob > F          =     0.4688

--------------------------------------------------------------------------------
                 |             Linearized
     binarypnc7 |      Coef.   Std. Err.      t    P>|t|     [95% Conf. Interval]
-----------------+--------------------------------------------------------------
     clinic5_pnc |   .2440603   .5101461     0.48   0.633    -.7576759    1.245796
         hc5_pnc |   .3724995   .2353896     1.58   0.114    -.0897177    .8347167
       hosp5_pnc |   .6846773   .4711668     1.45   0.147    -.2405181    1.609873
    clinic10_pnc |   .1950144   .2300091     0.85   0.397    -.2566374    .6466662
        hc10_pnc |   .2251459   .2219502     1.01   0.311    -.2106812    .6609731
      hosp10_pnc |   .3799208   .2321174     1.64   0.102     -.075871    .8357125
    clinic15_pnc |   .0184957     .24624     0.08   0.940    -.4650275    .5020188
        hc15_pnc |  -.1153795   .2870314    -0.40   0.688    -.6790018    .4482429
      hosp15_pnc |   .0610503    .221403     0.28   0.783    -.3737023    .4958029
                 |
        religion |
 other christian  |   .0815087   .3226888     0.25   0.801    -.5521315    .7151489
muslim/no religion  |  -.0291353    .423732    -0.07   0.945    -.8611864    .8029158
                 |
            v024 |
  central region  |  -.2804139   .3482708    -0.81   0.421    -.9642875    .4034597
 southern region  |  -.5468548   .3541319    -1.54   0.123    -1.242237    .1485279
                 |
         seasons |
winter-dry season  |  -.2276981   .3168302    -0.72   0.473    -.8498342     .394438
  hot-dry season  |  -.2747952   .2860364    -0.96   0.337    -.8364636    .2868733
                 |
       1.tvradio |   .0388962   .2398108     0.16   0.871    -.4320026     .509795
 1.moneyfortreat |   .2288167   .2253334     1.02   0.310    -.2136538    .6712872
                 |
             age |
          25-34  |   .3605479   .3498309     1.03   0.303    -.3263892    1.047485
          35-49  |   .3117922   .4228843     0.74   0.461    -.5185944    1.142179
                 |
       education |
        primary  |    .061771   .3345601     0.18   0.854    -.5951799    .7187219
      secondary  |  -1.459007   .9081137    -1.61   0.109    -3.242202     .324189
                 |
    1.employment |   .2581898   .2629074     0.98   0.326    -.2580621    .7744417
                 |
     wealthrural |
```

```
                 2  |   .6896269   .3163338     2.18   0.030     .0684656    1.310788
                 3  |   .6873397   .3431183     2.00   0.046     .0135838    1.361096
                 4  |   .6772464   .3492505     1.94   0.053    -.0085508    1.363044
                 5  |   .4112994   .4111239     1.00   0.317    -.3959941    1.218593
                    |
           birthnum |
               2-3  |   -.382392   .4204984    -0.91   0.363    -1.208094    .4433096
                4+  |  -.7211078   .5108291    -1.41   0.159    -1.724185    .2819693
                    |
              nsize |
                 2  |   -.558089   .4145423    -1.35   0.179    -1.372095     .255917
                 3  |  -.6566116   .4207847    -1.56   0.119    -1.482875    .1696523
                 4  |  -.5672522   .4403974    -1.29   0.198    -1.432028    .2975235
                 5  |   -.575567   .6108505    -0.94   0.346    -1.775049     .623915
                    |
               nsex |
            female  |    .134599   .2596515     0.52   0.604    -.3752595    .6444574
              _cons |  -1.157522   .6551976    -1.77   0.078    -2.444085    .1290411
------------------------------------------------------------------------------------
Note: 28 strata omitted because they contain no subpopulation members.

. margins, dydx(*)

Average marginal effects                        Number of obs     =     10,781
Model VCE    : Linearized

Expression   : Pr(binarypnc7), predict()
dy/dx w.r.t. : clinic5_pnc hc5_pnc hosp5_pnc clinic10_pnc hc10_pnc hosp10_pnc clinic15_pnc
hc15_pnc hosp15_pnc
               2.religion 3.religion 2.v024 3.v024 2.seasons 3.seasons 1.tvradio
1.moneyfortreat 2.age 3.age
               2.education 3.education 1.employment 2.wealthrural 3.wealthrural
4.wealthrural 5.wealthrural
               2.birthnum 3.birthnum 2.nsize 3.nsize 4.nsize 5.nsize 1.nsex

------------------------------------------------------------------------------------
                    |            Delta-method
                    |      dy/dx   Std. Err.      t    P>|t|     [95% Conf. Interval]
--------------------+---------------------------------------------------------------
        clinic5_pnc |   .0453458   .0947029     0.48   0.632    -.1406152    .2313069
            hc5_pnc |   .0692095   .0446416     1.55   0.122    -.0184499     .156869
          hosp5_pnc |   .1272115   .0867431     1.47   0.143    -.0431196    .2975425
       clinic10_pnc |   .0362332   .0427696     0.85   0.397    -.0477502    .1202167
           hc10_pnc |   .0418316   .0414485     1.01   0.313    -.0395578     .123221
         hosp10_pnc |   .0705884    .043302     1.63   0.104    -.0144405    .1556173
       clinic15_pnc |   .0034365   .0457299     0.08   0.940      -.08636    .0932329
           hc15_pnc |  -.0214372   .0532107    -0.40   0.687    -.1259231    .0830486
         hosp15_pnc |    .011343   .0409965     0.28   0.782    -.0691588    .0918448
                    |
           religion |
   other christian  |   .0150881   .0590507     0.26   0.798    -.1008653    .1310416
muslim/no religion  |  -.0052729   .0766621    -0.07   0.945    -.1558086    .1452627
                    |
               v024 |
    central region  |  -.0564905   .0717685    -0.79   0.431    -.1974171    .0844361
```

```
   southern region  |  -.1050912   .0712816    -1.47   0.141    -.2450616    .0348791
                    |
            seasons |
  winter-dry season |   -.042557   .0583988    -0.73   0.466    -.1572304    .0721163
     hot-dry season |  -.0508736    .051538    -0.99   0.324     -.152075    .0503279
                    |
          1.tvradio |   .0072412   .0447568     0.16   0.872    -.0806444    .0951267
     1.moneyfortreat |   .0422259   .0413739     1.02   0.308     -.039017    .1234688
                    |
                age |
              25-34 |   .0658161   .0631116     1.04   0.297    -.0581114    .1897436
              35-49 |    .056373   .0772666     0.73   0.466    -.0953497    .2080957
                    |
          education |
            primary |   .0118427   .0646502     0.18   0.855     -.115106    .1387914
          secondary |  -.1925346   .0753518    -2.56   0.011    -.3404973   -.0445718
                    |
       1.employment |   .0470784   .0468576     1.00   0.315    -.0449323    .1390891
                    |
        wealthrural |
                  2 |   .1214012   .0565197     2.15   0.032     .0104176    .2323849
                  3 |   .1209385   .0611421     1.98   0.048     .0008784    .2409986
                  4 |   .1189006   .0615573     1.93   0.054    -.001975     .2397762
                  5 |   .0678605   .0697002     0.97   0.331    -.0690046    .2047256
                    |
           birthnum |
                2-3 |   -.077358   .0874767    -0.88   0.377    -.2491294    .0944134
                 4+ |  -.1373767   .1010995    -1.36   0.175    -.3358983    .0611448
                    |
              nsize |
                  2 |  -.1142897   .0886015    -1.29   0.198      -.28827    .0596906
                  3 |  -.1322783   .0895722    -1.48   0.140    -.3081646     .043608
                  4 |  -.1159939   .0924745    -1.25   0.210    -.2975793    .0655915
                  5 |  -.1175348   .1207227    -0.97   0.331    -.3545891    .1195195
                    |
               nsex |
             female |    .025013   .0482086     0.52   0.604    -.0696507    .1196768
------------------------------------------------------------------------------------
Note: dy/dx for factor levels is the discrete change from the base level.

.
. /******
> Facility
> Delivery
> Only
> *******/
.
. ****Survey weights
. gen weight = v005/1000000
(1 missing value generated)

. svyset [pweight=weight], psu(v021) strata(v022)

      pweight: weight
          VCE: linearized
```

```
  Single unit: missing
     Strata 1: v022
         SU 1: v021
        FPC 1: <zero>


. ***Analysis commands
. svy, subpop(del if residence == 1):logit specialmpnc7 clinic5_pnc hc5_pnc hosp5_pnc ///
> clinic10_pnc hc10_pnc hosp10_pnc ///
> clinic15_pnc hc15_pnc hosp15_pnc ///
> i.religion i.mhealthcheckb4 i.v024 i.csect i.seasons i.tvradio i.moneyfortreat i.age
i.education i.employment i.wealt
> hrural ///
> i.birthnum i.nsize i.nsex
(running logit on estimation sample)

Survey: Logistic regression

Number of strata   =        28                  Number of obs     =     10,774
Number of PSUs     =       677                  Population size   = 11,193.203
                                                Subpop. no. obs   =     10,083
                                                Subpop. size      = 10,394.781
                                                Design df         =        649
                                                F(  35,    615)   =       8.38
                                                Prob > F          =     0.0000

------------------------------------------------------------------------------------
                    |             Linearized
       specialmpnc7 |      Coef.   Std. Err.      t    P>|t|     [95% Conf. Interval]
--------------------+---------------------------------------------------------------
        clinic5_pnc |  -.1713843   .2521968    -0.68   0.497    -.6666045    .3238358
            hc5_pnc |   .2877873   .1240143     2.32   0.021     .0442695     .531305
          hosp5_pnc |   .2992524   .1804548     1.66   0.098    -.0550932    .6535981
       clinic10_pnc |  -.0002366   .1540629    -0.00   0.999    -.3027585    .3022854
           hc10_pnc |   .4077845   .1317325     3.10   0.002     .1491112    .6664579
         hosp10_pnc |  -.3143191   .1357553    -2.32   0.021    -.5808917   -.0477466
       clinic15_pnc |   -.003234   .1436078    -0.02   0.982     -.285226    .2787581
           hc15_pnc |   .0831673   .1562004     0.53   0.595    -.2235518    .3898864
         hosp15_pnc |  -.0333368   .1334811    -0.25   0.803    -.2954436    .2287701
                    |
           religion |
   other christian  |  -.1521579   .0986293    -1.54   0.123    -.3458289    .0415131
muslim/no religion  |  -.3150643   .1525993    -2.06   0.039    -.6147123   -.0154164
                    |
    1.mhealthcheckb4 |   1.167813   .0812546    14.37   0.000     1.008259    1.327367
                    |
               v024 |
    central region  |  -.6765987   .1658213    -4.08   0.000     -1.00221   -.3509877
   southern region  |  -.3003476   .1605883    -1.87   0.062     -.615683    .0149878
                    |
            1.csect |   .0679381   .1324842     0.51   0.608    -.1922113    .3280875
                    |
            seasons |
 winter-dry season  |  -.0302841   .0764426    -0.40   0.692    -.1803887    .1198206
    hot-dry season  |   .0690617   .0956194     0.72   0.470     -.118699    .2568224
```

```
                    |
          1.tvradio |  -.0321294   .0855245    -0.38   0.707    -.2000676    .1358088
    1.moneyfortreat |   .0725906   .0872824     0.83   0.406    -.0987994    .2439806
                    |
                age |
             25-34  |   .0662468   .1023572     0.65   0.518    -.1347445    .2672382
             35-49  |   .1706502   .1317131     1.30   0.196    -.0879851    .4292854
                    |
          education |
           primary  |   .1048189   .0941061     1.11   0.266    -.0799701     .289608
         secondary  |   .3719539   .1709274     2.18   0.030     .0363165    .7075913
                    |
       1.employment |    .411003    .094257     4.36   0.000     .2259174    .5960885
                    |
        wealthrural |
                 2  |   .1384609   .1052391     1.32   0.189    -.0681894    .3451112
                 3  |   .0587061   .1054992     0.56   0.578    -.1484548    .2658671
                 4  |   .1368959   .1210954     1.13   0.259    -.1008901     .374682
                 5  |   .1857292    .139833     1.33   0.185    -.0888505     .460309
                    |
           birthnum |
               2-3  |   .2244772   .1102921     2.04   0.042     .0079049    .4410496
                4+  |   .1145269   .1387802     0.83   0.410    -.1579854    .3870392
                    |
              nsize |
                 2  |  -.0370872   .1468683    -0.25   0.801    -.3254815    .2513072
                 3  |  -.1402247   .1309325    -1.07   0.285    -.3973271    .1168777
                 4  |  -.0707794   .1557214    -0.45   0.650     -.376558    .2349993
                 5  |  -.6398211    .223486    -2.86   0.004    -1.078664   -.2009781
                    |
               nsex |
            female  |   .0184342   .0700058     0.26   0.792    -.1190311    .1558994
              _cons |  -2.968161   .2947459   -10.07   0.000    -3.546931    -2.38939
------------------------------------------------------------------------------------
Note: 28 strata omitted because they contain no subpopulation members.

. margins, dydx(*)

Average marginal effects                        Number of obs     =     10,083
Model VCE    : Linearized

Expression   : Pr(specialmpnc7), predict()
dy/dx w.r.t. : clinic5_pnc hc5_pnc hosp5_pnc clinic10_pnc hc10_pnc hosp10_pnc clinic15_pnc
hc15_pnc hosp15_pnc
               2.religion 3.religion 1.mhealthcheckb4 2.v024 3.v024 1.csect 2.seasons
3.seasons 1.tvradio
               1.moneyfortreat 2.age 3.age 2.education 3.education 1.employment
2.wealthrural 3.wealthrural
               4.wealthrural 5.wealthrural 2.birthnum 3.birthnum 2.nsize 3.nsize 4.nsize
5.nsize 1.nsex

------------------------------------------------------------------------------------
                    |            Delta-method
                    |      dy/dx   Std. Err.      t    P>|t|     [95% Conf. Interval]
--------------------+---------------------------------------------------------------
```

| | | | | | | |
|---|---|---|---|---|---|---|
| clinic5_pnc | -.0192471 | .028343 | -0.68 | 0.497 | -.0749021 | .0364078 |
| hc5_pnc | .0323196 | .0138848 | 2.33 | 0.020 | .0050551 | .0595842 |
| hosp5_pnc | .0336072 | .0204014 | 1.65 | 0.100 | -.0064535 | .073668 |
| clinic10_pnc | -.0000266 | .0173019 | -0.00 | 0.999 | -.034001 | .0339479 |
| hc10_pnc | .0457958 | .0150406 | 3.04 | 0.002 | .0162618 | .0753298 |
| hosp10_pnc | -.0352993 | .015333 | -2.30 | 0.022 | -.0654076 | -.0051909 |
| clinic15_pnc | -.0003632 | .0161292 | -0.02 | 0.982 | -.0320348 | .0313084 |
| hc15_pnc | .00934 | .0175674 | 0.53 | 0.595 | -.0251558 | .0438358 |
| hosp15_pnc | -.0037438 | .014966 | -0.25 | 0.803 | -.0331314 | .0256437 |
| | | | | | | |
| religion | | | | | | |
| other christian | -.0179044 | .0119663 | -1.50 | 0.135 | -.0414017 | .0055928 |
| muslim/no religion | -.0352706 | .0168611 | -2.09 | 0.037 | -.0683795 | -.0021617 |
| | | | | | | |
| 1.mhealthcheckb4 | .130677 | .009676 | 13.51 | 0.000 | .111677 | .149677 |
| | | | | | | |
| v024 | | | | | | |
| central region | -.0799752 | .0207653 | -3.85 | 0.000 | -.1207504 | -.0391999 |
| southern region | -.0396011 | .0219927 | -1.80 | 0.072 | -.0827866 | .0035844 |
| | | | | | | |
| 1.csect | .0077714 | .0153973 | 0.50 | 0.614 | -.022463 | .0380059 |
| | | | | | | |
| seasons | | | | | | |
| winter-dry season | -.0033614 | .0084798 | -0.40 | 0.692 | -.0200125 | .0132897 |
| hot-dry season | .0079066 | .0109906 | 0.72 | 0.472 | -.0136749 | .0294881 |
| | | | | | | |
| 1.tvradio | -.0036004 | .0095702 | -0.38 | 0.707 | -.0223926 | .0151918 |
| 1.moneyfortreat | .0081266 | .0098056 | 0.83 | 0.408 | -.011128 | .0273811 |
| | | | | | | |
| age | | | | | | |
| 25-34 | .0073065 | .0112183 | 0.65 | 0.515 | -.0147221 | .0293352 |
| 35-49 | .0194492 | .015073 | 1.29 | 0.197 | -.0101486 | .0490469 |
| | | | | | | |
| education | | | | | | |
| primary | .0118626 | .0107865 | 1.10 | 0.272 | -.0093179 | .0330432 |
| secondary | .0456694 | .0230566 | 1.98 | 0.048 | .0003948 | .0909441 |
| | | | | | | |
| 1.employment | .0438846 | .0093979 | 4.67 | 0.000 | .0254306 | .0623386 |
| | | | | | | |
| wealthrural | | | | | | |
| 2 | .0152595 | .0117217 | 1.30 | 0.193 | -.0077575 | .0382765 |
| 3 | .0063066 | .0113466 | 0.56 | 0.579 | -.0159739 | .0285871 |
| 4 | .0150795 | .0134122 | 1.12 | 0.261 | -.0112571 | .0414161 |
| 5 | .020778 | .0159108 | 1.31 | 0.192 | -.0104649 | .0520208 |
| | | | | | | |
| birthnum | | | | | | |
| 2-3 | .0249585 | .0119408 | 2.09 | 0.037 | .0015113 | .0484057 |
| 4+ | .0122931 | .0148323 | 0.83 | 0.408 | -.016832 | .0414182 |
| | | | | | | |
| nsize | | | | | | |
| 2 | -.0044108 | .017532 | -0.25 | 0.801 | -.038837 | .0300155 |
| 3 | -.0161621 | .0155438 | -1.04 | 0.299 | -.0466844 | .0143602 |
| 4 | -.0083325 | .0183818 | -0.45 | 0.650 | -.0444275 | .0277625 |
| 5 | -.062973 | .0193413 | -3.26 | 0.001 | -.1009521 | -.024994 |

```
                nsex |
              female |   .0020704    .007877     0.26   0.793     -.013397    .0175378
------------------------------------------------------------------------------------
Note: dy/dx for factor levels is the discrete change from the base level.

.
. svy, subpop(del if residence == 1):logit specialpnc7 clinic5_pnc hc5_pnc hosp5_pnc ///
> clinic10_pnc hc10_pnc hosp10_pnc ///
> clinic15_pnc hc15_pnc hosp15_pnc ///
> i.religion i.newborncheckb4 i.v024 i.csect i.seasons i.tvradio i.moneyfortreat i.age
i.education i.employment i.wealt
> hrural ///
> i.birthnum i.nsize i.nsex
(running logit on estimation sample)

Survey: Logistic regression

Number of strata   =        28                  Number of obs     =     10,720
Number of PSUs     =       677                  Population size   = 11,143.466
                                                Subpop. no. obs   =     10,029
                                                Subpop. size      = 10,345.044
                                                Design df         =        649
                                                F(  35,    615)   =       6.52
                                                Prob > F          =     0.0000

------------------------------------------------------------------------------------
                    |             Linearized
        specialpnc7 |      Coef.   Std. Err.      t    P>|t|     [95% Conf. Interval]
--------------------+---------------------------------------------------------------
        clinic5_pnc |  -.7972276   .2244289    -3.55   0.000    -1.237922   -.3565332
            hc5_pnc |   .0694486   .0909467     0.76   0.445    -.1091367    .2480338
          hosp5_pnc |   .1815618   .1307986     1.39   0.166    -.0752778    .4384013
       clinic10_pnc |  -.2507205   .1271165    -1.97   0.049    -.5003297   -.0011113
           hc10_pnc |   .3851476   .0996665     3.86   0.000     .1894399    .5808553
         hosp10_pnc |  -.0651567   .0937966    -0.69   0.488    -.2493381    .1190248
       clinic15_pnc |   .1727465   .1162374     1.49   0.138    -.0555004    .4009933
           hc15_pnc |   .1280178   .1199678     1.07   0.286    -.1075542    .3635898
         hosp15_pnc |  -.0715044   .0911826    -0.78   0.433     -.250553    .1075441
                    |
           religion |
    other christian |  -.0870707   .0807371    -1.08   0.281    -.2456081    .0714667
muslim/no religion  |  -.1483652   .1354283    -1.10   0.274    -.4142959    .1175654
                    |
   1.newborncheckb4 |   .5879751   .0758647     7.75   0.000     .4390052     .736945
                    |
               v024 |
     central region |  -.8264429   .1231042    -6.71   0.000    -1.068174   -.5847122
    southern region |  -.6545487   .1256078    -5.21   0.000    -.9011954   -.4079019
                    |
            1.csect |  -.3920524     .13414    -2.92   0.004    -.6554532   -.1286515
                    |
            seasons |
 winter-dry season  |  -.0018992   .0630699    -0.03   0.976    -.1257449    .1219466
    hot-dry season  |  -.0497317   .0831904    -0.60   0.550    -.2130866    .1136232
                    |
```

```
          1.tvradio |  -.0781802   .0633228    -1.23   0.217    -.2025225     .046162
    1.moneyfortreat |    .010146   .0661289     0.15   0.878    -.1197064    .1399984
                    |
                age |
              25-34  |  -.1252781   .0883017    -1.42   0.156    -.2986695    .0481133
              35-49  |  -.2341825   .1185032    -1.98   0.049    -.4668786   -.0014865
                    |
          education |
            primary  |   .1121422   .0782773     1.43   0.152    -.0415651    .2658495
          secondary  |   .2118852   .1439779     1.47   0.142    -.0708335     .494604
                    |
       1.employment |   .4055731   .0725054     5.59   0.000     .2631996    .5479466
                    |
        wealthrural |
                  2  |   .1050628   .1004956     1.05   0.296    -.0922729    .3023985
                  3  |   .1804227   .1022513     1.76   0.078    -.0203606     .381206
                  4  |    .220971   .1023244     2.16   0.031     .0200442    .4218978
                  5  |   .3045234   .1084705     2.81   0.005     .0915279    .5175189
                    |
          birthnum |
                2-3  |   .2114188   .0934564     2.26   0.024     .0279053    .3949323
                 4+  |    .296561    .113938     2.60   0.009     .0728294    .5202927
                    |
              nsize |
                  2  |   .1699364   .1137867     1.49   0.136    -.0534981     .393371
                  3  |    .062934   .1091068     0.58   0.564     -.151311     .277179
                  4  |   .0153457   .1290438     0.12   0.905     -.238048    .2687394
                  5  |  -.2054025   .1651701    -1.24   0.214    -.5297349    .1189299
                    |
               nsex |
             female  |  -.0788647   .0519179    -1.52   0.129     -.180812    .0230826
              _cons  |  -1.757281   .2089057    -8.41   0.000    -2.167494   -1.347068
------------------------------------------------------------------------------------
Note: 28 strata omitted because they contain no subpopulation members.

. margins, dydx(*)

Average marginal effects                        Number of obs     =     10,029
Model VCE    : Linearized

Expression   : Pr(specialpnc7), predict()
dy/dx w.r.t. : clinic5_pnc hc5_pnc hosp5_pnc clinic10_pnc hc10_pnc hosp10_pnc clinic15_pnc
hc15_pnc hosp15_pnc
               2.religion 3.religion 1.newborncheckb4 2.v024 3.v024 1.csect 2.seasons
3.seasons 1.tvradio
               1.moneyfortreat 2.age 3.age 2.education 3.education 1.employment
2.wealthrural 3.wealthrural
               4.wealthrural 5.wealthrural 2.birthnum 3.birthnum 2.nsize 3.nsize 4.nsize
5.nsize 1.nsex

------------------------------------------------------------------------------------
                    |            Delta-method
                    |      dy/dx   Std. Err.      t    P>|t|     [95% Conf. Interval]
--------------------+---------------------------------------------------------------
        clinic5_pnc |  -.1382724   .0389977    -3.55   0.000    -.2148493   -.0616955
```

| | | | | | | |
|---|---|---|---|---|---|---|
| hc5_pnc | .0120453 | .0157414 | 0.77 | 0.444 | -.0188649 | .0429555 |
| hosp5_pnc | .0314904 | .0227019 | 1.39 | 0.166 | -.0130877 | .0760684 |
| clinic10_pnc | -.0434853 | .0219244 | -1.98 | 0.048 | -.0865367 | -.000434 |
| hc10_pnc | .0668006 | .0173388 | 3.85 | 0.000 | .0327538 | .1008474 |
| hosp10_pnc | -.0113009 | .016294 | -0.69 | 0.488 | -.0432963 | .0206945 |
| clinic15_pnc | .0299614 | .0201963 | 1.48 | 0.138 | -.0096966 | .0696194 |
| hc15_pnc | .0222036 | .0208396 | 1.07 | 0.287 | -.0187176 | .0631248 |
| hosp15_pnc | -.0124018 | .0158295 | -0.78 | 0.434 | -.0434851 | .0186814 |
| religion | | | | | | |
| other christian | -.0153791 | .0144532 | -1.06 | 0.288 | -.0437598 | .0130016 |
| muslim/no religion | -.0258356 | .0233601 | -1.11 | 0.269 | -.0717061 | .020035 |
| 1.newborncheckb4 | .0975287 | .0120471 | 8.10 | 0.000 | .0738726 | .1211847 |
| v024 | | | | | | |
| central region | -.1590754 | .0248363 | -6.40 | 0.000 | -.2078446 | -.1103062 |
| southern region | -.1303944 | .0262167 | -4.97 | 0.000 | -.1818742 | -.0789147 |
| 1.csect | -.0623722 | .0195298 | -3.19 | 0.001 | -.1007214 | -.024023 |
| seasons | | | | | | |
| winter-dry season | -.0003308 | .0109875 | -0.03 | 0.976 | -.0219062 | .0212445 |
| hot-dry season | -.0085666 | .0142961 | -0.60 | 0.549 | -.0366389 | .0195057 |
| 1.tvradio | -.0134992 | .0108907 | -1.24 | 0.216 | -.0348845 | .0078861 |
| 1.moneyfortreat | .0017591 | .011466 | 0.15 | 0.878 | -.020756 | .0242741 |
| age | | | | | | |
| 25-34 | -.0220782 | .015651 | -1.41 | 0.159 | -.0528109 | .0086544 |
| 35-49 | -.0402362 | .020195 | -1.99 | 0.047 | -.0798916 | -.0005809 |
| education | | | | | | |
| primary | .0196743 | .0139295 | 1.41 | 0.158 | -.0076779 | .0470266 |
| secondary | .0380163 | .026941 | 1.41 | 0.159 | -.0148858 | .0909184 |
| 1.employment | .067877 | .0115029 | 5.90 | 0.000 | .0452897 | .0904644 |
| wealthrural | | | | | | |
| 2 | .0174049 | .016749 | 1.04 | 0.299 | -.0154839 | .0502938 |
| 3 | .0304523 | .0172714 | 1.76 | 0.078 | -.0034623 | .0643668 |
| 4 | .037664 | .0174288 | 2.16 | 0.031 | .0034404 | .0718876 |
| 5 | .0529382 | .0189081 | 2.80 | 0.005 | .0158097 | .0900667 |
| birthnum | | | | | | |
| 2-3 | .0350953 | .0151343 | 2.32 | 0.021 | .0053772 | .0648134 |
| 4+ | .0502606 | .0190068 | 2.64 | 0.008 | .0129384 | .0875827 |
| nsize | | | | | | |
| 2 | .0297394 | .0195669 | 1.52 | 0.129 | -.0086827 | .0681616 |
| 3 | .0107398 | .0184608 | 0.58 | 0.561 | -.0255104 | .0469899 |
| 4 | .0025887 | .0217606 | 0.12 | 0.905 | -.040141 | .0453184 |
| 5 | -.0327587 | .0257407 | -1.27 | 0.204 | -.0833039 | .0177864 |
| nsex | | | | | | |

```
           female  |   -.013678   .0090093    -1.52   0.129    -.0313688    .0040129
------------------------------------------------------------------------------------
Note: dy/dx for factor levels is the discrete change from the base level.

.
. ***Appendix analysis
. svy, subpop(del if residence == 1):logit binarympnc1 clinic5_pnc hc5_pnc hosp5_pnc ///
> clinic10_pnc hc10_pnc hosp10_pnc ///
> clinic15_pnc hc15_pnc hosp15_pnc ///
> i.religion i.mhealthcheckb4 i.v024 i.csect i.seasons i.tvradio i.moneyfortreat i.age
i.education i.employment i.wealt
> hrural ///
> i.birthnum i.nsize i.nsex
(running logit on estimation sample)

Survey: Logistic regression

Number of strata   =        28                Number of obs     =     10,774
Number of PSUs     =       677                Population size   = 11,193.203
                                              Subpop. no. obs   =     10,083
                                              Subpop. size      = 10,394.781
                                              Design df         =        649
                                              F(  35,    615)   =       3.62
                                              Prob > F          =     0.0000

------------------------------------------------------------------------------------
                   |             Linearized
       binarympnc1 |      Coef.   Std. Err.      t    P>|t|     [95% Conf. Interval]
-------------------+----------------------------------------------------------------
       clinic5_pnc |   .6902585   .4632379     1.49   0.137    -.2193675    1.599884
           hc5_pnc |  -.1427565   .2071828    -0.69   0.491    -.5495861     .264073
         hosp5_pnc |  -.8636086   .3499622    -2.47   0.014    -1.550804   -.1764138
      clinic10_pnc |   .1737064   .2927978     0.59   0.553    -.4012389    .7486517
          hc10_pnc |   .0075948   .2364449     0.03   0.974    -.4566945    .4718842
        hosp10_pnc |  -.1110662   .2197209    -0.51   0.613    -.5425158    .3203834
      clinic15_pnc |  -.4459529   .2342792    -1.90   0.057    -.9059897    .0140839
          hc15_pnc |  -.2770907   .2328956    -1.19   0.235    -.7344105    .1802292
        hosp15_pnc |  -.4099068   .2138935    -1.92   0.056    -.8299137    .0101001
                   |
          religion |
   other christian  |   .1252275   .2164145     0.58   0.563    -.2997296    .5501845
muslim/no religion  |  -.9873748   .3815415    -2.59   0.010     -1.73658   -.2381701
                   |
    1.mhealthcheckb4 |   1.156496   .1830431     6.32   0.000     .7970683    1.515925
                   |
              v024 |
    central region  |  -1.267796   .2990112    -4.24   0.000    -1.854942   -.6806496
   southern region  |  -.1300683   .2731065    -0.48   0.634    -.6663472    .4062106
                   |
           1.csect |   .1728796   .3299634     0.52   0.601     -.475045    .8208042
                   |
           seasons |
 winter-dry season  |  -.4740604   .1814635    -2.61   0.009    -.8303869   -.1177339
    hot-dry season  |  -.3086193   .2202082    -1.40   0.162    -.7410259    .1237872
                   |
```

```
        1.tvradio |   .3051564   .1831914     1.67   0.096     -.054563    .6648758
  1.moneyfortreat |    .297055   .1796392     1.65   0.099    -.0556892    .6497993
                  |
              age |
           25-34  |   -.087365   .2123842    -0.41   0.681    -.5044082    .3296782
           35-49  |  -.1220482   .3107401    -0.39   0.695    -.7322256    .4881292
                  |
        education |
         primary  |  -.1094304   .1891875    -0.58   0.563    -.4809238     .262063
       secondary  |  -.0496985   .5434327    -0.09   0.927    -1.116797      1.0174
                  |
     1.employment |  -.1322235   .1641315    -0.81   0.421    -.4545163    .1900693
                  |
      wealthrural |
               2  |   .2252523   .2407341     0.94   0.350    -.2474593     .697964
               3  |   .0686592   .2507396     0.27   0.784    -.4236995    .5610179
               4  |  -.2252098   .2868466    -0.79   0.433    -.7884693    .3380497
               5  |    -.02881   .3066573    -0.09   0.925    -.6309703    .5733503
                  |
         birthnum |
             2-3  |   .0317651   .2220079     0.14   0.886    -.4041754    .4677056
              4+  |  -.0569746   .2855045    -0.20   0.842    -.6175988    .5036495
                  |
            nsize |
               2  |  -.0449821   .3407779    -0.13   0.895    -.7141423    .6241782
               3  |   .2168155   .3231662     0.67   0.503     -.417762    .8513931
               4  |  -.0276027   .4102415    -0.07   0.946    -.8331636    .7779581
               5  |   .1195827   .4880024     0.25   0.806    -.8386715    1.077837
                  |
             nsex |
          female  |   .0747679   .1539286     0.49   0.627    -.2274903     .377026
           _cons  |  -3.612884   .6723342    -5.37   0.000    -4.933097   -2.292671
------------------------------------------------------------------------------------
Note: 28 strata omitted because they contain no subpopulation members.

. margins, dydx(*)

Average marginal effects                        Number of obs     =     10,083
Model VCE    : Linearized

Expression   : Pr(binarympnc1), predict()
dy/dx w.r.t. : clinic5_pnc hc5_pnc hosp5_pnc clinic10_pnc hc10_pnc hosp10_pnc clinic15_pnc
hc15_pnc hosp15_pnc
               2.religion 3.religion 1.mhealthcheckb4 2.v024 3.v024 1.csect 2.seasons
3.seasons 1.tvradio
               1.moneyfortreat 2.age 3.age 2.education 3.education 1.employment
2.wealthrural 3.wealthrural
               4.wealthrural 5.wealthrural 2.birthnum 3.birthnum 2.nsize 3.nsize 4.nsize
5.nsize 1.nsex

------------------------------------------------------------------------------------
                  |            Delta-method
                  |      dy/dx   Std. Err.      t    P>|t|     [95% Conf. Interval]
------------------+-----------------------------------------------------------------
      clinic5_pnc |   .0138646   .0095082     1.46   0.145    -.0048058    .0325351
```

| | | | | | | |
|---|---|---|---|---|---|---|
| hc5_pnc | -.0028674 | .0041612 | -0.69 | 0.491 | -.0110385 | .0053036 |
| hosp5_pnc | -.0173466 | .0072646 | -2.39 | 0.017 | -.0316116 | -.0030815 |
| clinic10_pnc | .0034891 | .0059298 | 0.59 | 0.556 | -.0081549 | .015133 |
| hc10_pnc | .0001526 | .0047511 | 0.03 | 0.974 | -.0091769 | .009482 |
| hosp10_pnc | -.0022309 | .00444 | -0.50 | 0.616 | -.0109494 | .0064876 |
| clinic15_pnc | -.0089575 | .0049599 | -1.81 | 0.071 | -.0186969 | .0007819 |
| hc15_pnc | -.0055657 | .0046706 | -1.19 | 0.234 | -.0147369 | .0036056 |
| hosp15_pnc | -.0082335 | .0043514 | -1.89 | 0.059 | -.0167779 | .000311 |
| religion | | | | | | |
| other christian | .0027593 | .0045821 | 0.60 | 0.547 | -.0062382 | .0117568 |
| muslim/no religion | -.0134087 | .0052703 | -2.54 | 0.011 | -.0237576 | -.0030598 |
| 1.mhealthcheckb4 | .0221677 | .0037591 | 5.90 | 0.000 | .0147863 | .0295492 |
| v024 | | | | | | |
| central region | -.0232597 | .0074043 | -3.14 | 0.002 | -.0377989 | -.0087204 |
| southern region | -.0038151 | .0082522 | -0.46 | 0.644 | -.0200194 | .0123892 |
| 1.csect | .0037169 | .0076257 | 0.49 | 0.626 | -.0112571 | .0186909 |
| seasons | | | | | | |
| winter-dry season | -.0092688 | .0034888 | -2.66 | 0.008 | -.0161194 | -.0024182 |
| hot-dry season | -.006481 | .0044427 | -1.46 | 0.145 | -.0152048 | .0022428 |
| 1.tvradio | .0062964 | .0039514 | 1.59 | 0.112 | -.0014627 | .0140556 |
| 1.moneyfortreat | .0058825 | .0035745 | 1.65 | 0.100 | -.0011364 | .0129014 |
| age | | | | | | |
| 25-34 | -.0017803 | .0043474 | -0.41 | 0.682 | -.010317 | .0067564 |
| 35-49 | -.0024486 | .0061335 | -0.40 | 0.690 | -.0144925 | .0095953 |
| education | | | | | | |
| primary | -.0021465 | .0036468 | -0.59 | 0.556 | -.0093075 | .0050146 |
| secondary | -.0010015 | .0107415 | -0.09 | 0.926 | -.0220938 | .0200908 |
| 1.employment | -.0027087 | .0034063 | -0.80 | 0.427 | -.0093974 | .00398 |
| wealthrural | | | | | | |
| 2 | .0049418 | .0051898 | 0.95 | 0.341 | -.0052489 | .0151326 |
| 3 | .0014009 | .0050998 | 0.27 | 0.784 | -.0086132 | .011415 |
| 4 | -.0040219 | .0051773 | -0.78 | 0.438 | -.0141882 | .0061444 |
| 5 | -.0005622 | .0059831 | -0.09 | 0.925 | -.0123108 | .0111864 |
| birthnum | | | | | | |
| 2-3 | .0006535 | .0045356 | 0.14 | 0.885 | -.0082528 | .0095598 |
| 4+ | -.0011257 | .0056756 | -0.20 | 0.843 | -.0122705 | .0100192 |
| nsize | | | | | | |
| 2 | -.0008052 | .0061746 | -0.13 | 0.896 | -.0129297 | .0113194 |
| 3 | .0043828 | .006072 | 0.72 | 0.471 | -.0075404 | .016306 |
| 4 | -.000498 | .0074128 | -0.07 | 0.946 | -.0150541 | .014058 |
| 5 | .0023097 | .0095528 | 0.24 | 0.809 | -.0164484 | .0210678 |
| nsex | | | | | | |

```
           female  |   .0015027   .0030808     0.49   0.626    -.0045468    .0075522
------------------------------------------------------------------------------------
Note: dy/dx for factor levels is the discrete change from the base level.

.
. svy, subpop(del if residence == 1):logit binarypnc1 clinic5_pnc hc5_pnc hosp5_pnc ///
> clinic10_pnc hc10_pnc hosp10_pnc ///
> clinic15_pnc hc15_pnc hosp15_pnc ///
> i.religion i.newborncheckb4 i.v024 i.csect i.seasons i.tvradio i.moneyfortreat i.age
i.education i.employment i.wealt
> hrural ///
> i.birthnum i.nsize i.nsex
(running logit on estimation sample)

Survey: Logistic regression

Number of strata   =        28                Number of obs     =     10,720
Number of PSUs     =       677                Population size   = 11,143.466
                                              Subpop. no. obs   =     10,029
                                              Subpop. size      = 10,345.044
                                              Design df         =        649
                                              F(  35,    615)   =       2.26
                                              Prob > F          =     0.0001

------------------------------------------------------------------------------------
                   |             Linearized
        binarypnc1 |      Coef.   Std. Err.      t    P>|t|     [95% Conf. Interval]
-------------------+----------------------------------------------------------------
       clinic5_pnc |   .1273111   .4504968     0.28   0.778    -.7572961    1.011918
           hc5_pnc |  -.0981596   .2094017    -0.47   0.639    -.5093462     .313027
         hosp5_pnc |  -.4227043   .3100487    -1.36   0.173    -1.031524    .1861154
      clinic10_pnc |   .3026479   .2660244     1.14   0.256    -.2197245    .8250203
          hc10_pnc |   .0001427   .2240476     0.00   0.999     -.439803    .4400885
        hosp10_pnc |  -.2390088   .2111028    -1.13   0.258    -.6535357    .1755181
      clinic15_pnc |  -.2210003   .2337769    -0.95   0.345    -.6800506    .2380501
          hc15_pnc |   .0043599   .2748253     0.02   0.987    -.5352942     .544014
        hosp15_pnc |  -.4983598   .2125228    -2.34   0.019    -.9156751   -.0810445
                   |
          religion |
   other christian  |  -.0224034    .240471    -0.09   0.926    -.4945985    .4497917
muslim/no religion  |  -.9726906   .3827261    -2.54   0.011    -1.724221   -.2211597
                   |
   1.newborncheckb4 |   .8862694   .2173108     4.08   0.000     .4595522    1.312987
                   |
              v024 |
    central region  |  -.1434647   .3397785    -0.42   0.673    -.8106626    .5237333
   southern region  |   .7502831    .317386     2.36   0.018     .1270558     1.37351
                   |
           1.csect |  -.8385553   .5223252    -1.61   0.109    -1.864207    .1870962
                   |
           seasons |
 winter-dry season  |  -.0510674   .1684633    -0.30   0.762    -.3818662    .2797315
    hot-dry season  |  -.3306809   .2212887    -1.49   0.136    -.7652092    .1038474
                   |
         1.tvradio |   .5376109   .1647877     3.26   0.001     .2140295    .8611923
```

```
    1.moneyfortreat |   .2377566   .1825438     1.30   0.193    -.1206912    .5962043
                    |
                age |
             25-34  |  -.0750642    .245106    -0.31   0.760    -.5563607    .4062323
             35-49  |  -.0073627   .3122698    -0.02   0.981    -.6205438    .6058185
                    |
          education |
           primary  |  -.2246152   .2168105    -1.04   0.301      -.65035    .2011195
         secondary  |  -.5194271   .4823611    -1.08   0.282    -1.466604    .4277497
                    |
       1.employment |   .2425661   .1870795     1.30   0.195    -.1247881    .6099203
                    |
        wealthrural |
                 2  |   .1009932   .2310206     0.44   0.662    -.3526449    .5546313
                 3  |  -.0798653   .2300369    -0.35   0.729    -.5315717    .3718411
                 4  |   .0595342   .2551008     0.23   0.816    -.4413883    .5604568
                 5  |  -.5595173   .2893864    -1.93   0.054    -1.127764    .0087293
                    |
           birthnum |
               2-3  |  -.0201103   .2350157    -0.09   0.932    -.4815932    .4413726
                4+  |  -.2064249   .2945802    -0.70   0.484    -.7848702    .3720203
                    |
              nsize |
                 2  |   .2131295   .3146183     0.68   0.498    -.4046632    .8309222
                 3  |   .0938037   .2929898     0.32   0.749    -.4815186     .669126
                 4  |   .3092759   .3369274     0.92   0.359    -.3523235    .9708753
                 5  |  -.0210719   .4610583    -0.05   0.964     -.926418    .8842742
                    |
               nsex |
            female  |  -.0918444   .1525318    -0.60   0.547    -.3913598     .207671
              _cons |  -4.881232   .6187807    -7.89   0.000    -6.096286   -3.666178
------------------------------------------------------------------------------------
Note: 28 strata omitted because they contain no subpopulation members.

. margins, dydx(*)

Average marginal effects                         Number of obs     =     10,029
Model VCE    : Linearized

Expression   : Pr(binarypnc1), predict()
dy/dx w.r.t. : clinic5_pnc hc5_pnc hosp5_pnc clinic10_pnc hc10_pnc hosp10_pnc clinic15_pnc
hc15_pnc hosp15_pnc
               2.religion 3.religion 1.newborncheckb4 2.v024 3.v024 1.csect 2.seasons
3.seasons 1.tvradio
               1.moneyfortreat 2.age 3.age 2.education 3.education 1.employment
2.wealthrural 3.wealthrural
               4.wealthrural 5.wealthrural 2.birthnum 3.birthnum 2.nsize 3.nsize 4.nsize
5.nsize 1.nsex

------------------------------------------------------------------------------------
                    |            Delta-method
                    |      dy/dx   Std. Err.      t    P>|t|     [95% Conf. Interval]
--------------------+---------------------------------------------------------------
        clinic5_pnc |   .0022669   .0080168     0.28   0.777    -.0134751     .018009
            hc5_pnc |  -.0017479   .0037304    -0.47   0.640    -.0090729    .0055772
```

| | | | | | | |
|---|---|---|---|---|---|---|
| hosp5_pnc | -.0075268 | .0055626 | -1.35 | 0.176 | -.0184496 | .0033961 |
| clinic10_pnc | .005389 | .0048174 | 1.12 | 0.264 | -.0040706 | .0148487 |
| hc10_pnc | 2.54e-06 | .0039895 | 0.00 | 0.999 | -.0078313 | .0078363 |
| hosp10_pnc | -.0042558 | .0038341 | -1.11 | 0.267 | -.0117846 | .0032729 |
| clinic15_pnc | -.0039352 | .0042139 | -0.93 | 0.351 | -.0122097 | .0043393 |
| hc15_pnc | .0000776 | .004894 | 0.02 | 0.987 | -.0095324 | .0096877 |
| hosp15_pnc | -.0088739 | .0038854 | -2.28 | 0.023 | -.0165034 | -.0012444 |
| religion | | | | | | |
| other christian | -.0004551 | .0049197 | -0.09 | 0.926 | -.0101155 | .0092052 |
| muslim/no religion | -.0130618 | .0053426 | -2.44 | 0.015 | -.0235527 | -.0025709 |
| 1.newborncheckb4 | .0132626 | .0029341 | 4.52 | 0.000 | .0075011 | .0190241 |
| v024 | | | | | | |
| central region | -.0017412 | .0042496 | -0.41 | 0.682 | -.0100859 | .0066035 |
| southern region | .0141958 | .0052039 | 2.73 | 0.007 | .0039772 | .0244143 |
| 1.csect | -.0106279 | .0045656 | -2.33 | 0.020 | -.019593 | -.0016628 |
| seasons | | | | | | |
| winter-dry season | -.0009511 | .0031207 | -0.30 | 0.761 | -.0070791 | .0051768 |
| hot-dry season | -.0054227 | .0033769 | -1.61 | 0.109 | -.0120536 | .0012083 |
| 1.tvradio | .0101063 | .0034884 | 2.90 | 0.004 | .0032564 | .0169561 |
| 1.moneyfortreat | .0041382 | .0031424 | 1.32 | 0.188 | -.0020324 | .0103088 |
| age | | | | | | |
| 25-34 | -.0013296 | .0043747 | -0.30 | 0.761 | -.0099199 | .0072608 |
| 35-49 | -.0001346 | .0057055 | -0.02 | 0.981 | -.0113381 | .0110689 |
| education | | | | | | |
| primary | -.0038125 | .0034626 | -1.10 | 0.271 | -.0106118 | .0029868 |
| secondary | -.0077317 | .0057359 | -1.35 | 0.178 | -.0189949 | .0035315 |
| 1.employment | .0041182 | .003072 | 1.34 | 0.181 | -.001914 | .0101505 |
| wealthrural | | | | | | |
| 2 | .0019902 | .0045483 | 0.44 | 0.662 | -.006941 | .0109213 |
| 3 | -.0014462 | .0041681 | -0.35 | 0.729 | -.0096307 | .0067383 |
| 4 | .0011505 | .0049412 | 0.23 | 0.816 | -.0085522 | .0108532 |
| 5 | -.0081693 | .0042134 | -1.94 | 0.053 | -.0164429 | .0001044 |
| birthnum | | | | | | |
| 2-3 | -.0003865 | .0045297 | -0.09 | 0.932 | -.0092811 | .0085081 |
| 4+ | -.0036422 | .0052859 | -0.69 | 0.491 | -.0140217 | .0067373 |
| nsize | | | | | | |
| 2 | .0036813 | .00523 | 0.70 | 0.482 | -.0065884 | .013951 |
| 3 | .00153 | .0046492 | 0.33 | 0.742 | -.0075993 | .0106592 |
| 4 | .0055974 | .0060523 | 0.92 | 0.355 | -.006287 | .0174819 |
| 5 | -.0003255 | .007099 | -0.05 | 0.963 | -.0142652 | .0136143 |
| nsex | | | | | | |
| female | -.0016337 | .0027312 | -0.60 | 0.550 | -.0069967 | .0037293 |

```
-------------------------------------------------------------------------------------
Note: dy/dx for factor levels is the discrete change from the base level.

.
end of do-file

-------------------------------------------------------------------------------------------
----------------------------
```
